# Supplementary material for: A reproducible experimental survey on biomedical sentence similarity: A string-based method sets the state of the art
Source: PLoS One. 2022 Nov 21;17(11):e0276539. doi: 10.1371/journal.pone.0276539 (PMC9678326; doi:10.1371/journal.pone.0276539)
Supplement: S1 Appendix — We provide a series of tables reporting the p-values for each pair of methods evaluated in this work as supplementary material. (PDF) [file pone.0276539.s001.pdf]

Appendix A
Statistical significance results

This appendix shows the table with the p-values for each pair of methods evaluated in table 8, which is used to study the statistical significance of the results, as detailed in the Discussion section.

Table A.1: This table shows the resulting p-values comparing all the methods in table 8, which allows us to study the statistical significance of the results, as detailed in the Discussion section. The methods are sorted by the average harmonic score obtained from the 12 artificially created datasets detailed in the main paper.

	M4	M17	M7	M12	M26	M3	M33	M27	M32	M22	M2	M20	M19	M1	M23	M21	M24	M29	M43	M25	M15	M10	M14	M37	M28
M4	-	0.121	0.098	0.000	0.003	0.000	0.002	0.000	0.000	0.000	0.000	0.000	0.000	0.000	0.000	0.000	0.000	0.000	0.000	0.001	0.000	0.000	0.000	0.000	0.000
M17	0.121	-	0.088	0.000	0.004	0.003	0.003	0.000	0.001	0.001	0.000	0.000	0.000	0.001	0.000	0.000	0.000	0.000	0.000	0.001	0.000	0.000	0.000	0.000	0.000
M7	0.098	0.088	-	0.041	0.018	0.011	0.008	0.000	0.002	0.002	0.001	0.001	0.000	0.001	0.000	0.000	0.000	0.000	0.000	0.001	0.000	0.000	0.000	0.000	0.000
M12	0.000	0.000	0.041	-	0.049	0.010	0.011	0.001	0.001	0.001	0.000	0.001	0.000	0.001	0.000	0.000	0.000	0.000	0.000	0.002	0.000	0.000	0.000	0.000	0.000
M26	0.003	0.004	0.018	0.049	-	0.063	0.032	0.007	0.022	0.023	0.001	0.006	0.006	0.004	0.005	0.003	0.000	0.000	0.001	0.003	0.000	0.001	0.000	0.000	0.000
M3	0.000	0.003	0.011	0.010	0.063	-	0.178	0.150	0.083	0.085	0.003	0.067	0.032	0.006	0.029	0.031	0.004	0.002	0.007	0.017	0.000	0.007	0.001	0.001	0.000
M33	0.002	0.003	0.008	0.011	0.032	0.178	-	0.214	0.150	0.139	0.120	0.072	0.049	0.067	0.040	0.031	0.009	0.007	0.008	0.012	0.002	0.007	0.001	0.000	0.001
M27	0.000	0.000	0.000	0.001	0.007	0.150	0.214	-	0.182	0.178	0.130	0.079	0.037	0.055	0.018	0.008	0.000	0.006	0.001	0.004	0.000	0.001	0.000	0.000	0.000
M32	0.000	0.001	0.002	0.001	0.022	0.083	0.150	0.182	-	0.248	0.194	0.169	0.088	0.080	0.050	0.057	0.017	0.004	0.014	0.031	0.000	0.013	0.002	0.002	0.000
M22	0.000	0.001	0.002	0.001	0.023	0.085	0.139	0.178	0.248	-	0.195	0.137	0.045	0.097	0.041	0.048	0.013	0.002	0.018	0.024	0.002	0.013	0.002	0.001	0.000
M2	0.000	0.000	0.001	0.000	0.001	0.003	0.120	0.130	0.194	0.195	-	0.214	0.131	0.078	0.095	0.079	0.024	0.007	0.024	0.034	0.000	0.016	0.002	0.003	0.001
M20	0.000	0.000	0.001	0.001	0.006	0.067	0.072	0.079	0.169	0.137	0.214	-	0.090	0.156	0.072	0.044	0.018	0.006	0.026	0.014	0.006	0.011	0.004	0.002	0.000
M19	0.000	0.000	0.000	0.000	0.006	0.032	0.049	0.037	0.088	0.045	0.131	0.090	-	0.225	0.095	0.083	0.030	0.027	0.034	0.031	0.009	0.016	0.004	0.003	0.002
M1	0.000	0.001	0.001	0.001	0.004	0.006	0.067	0.055	0.080	0.097	0.078	0.156	0.225	-	0.205	0.173	0.103	0.035	0.040	0.068	0.009	0.032	0.012	0.008	0.003
M23	0.000	0.000	0.000	0.000	0.005	0.029	0.040	0.018	0.050	0.041	0.095	0.072	0.095	0.205	-	0.147	0.111	0.067	0.041	0.052	0.017	0.016	0.007	0.007	0.005
M21	0.000	0.000	0.000	0.000	0.003	0.031	0.031	0.008	0.057	0.048	0.079	0.044	0.083	0.173	0.147	-	0.199	0.098	0.068	0.038	0.040	0.009	0.012	0.014	0.008
M24	0.000	0.000	0.000	0.000	0.000	0.004	0.009	0.000	0.017	0.013	0.024	0.018	0.030	0.103	0.111	0.199	-	0.107	0.056	0.069	0.034	0.023	0.003	0.005	0.008
M29	0.000	0.000	0.000	0.000	0.000	0.002	0.007	0.006	0.004	0.002	0.007	0.006	0.027	0.035	0.067	0.098	0.107	-	0.232	0.212	0.170	0.157	0.143	0.102	0.000
M43	0.000	0.000	0.000	0.000	0.001	0.007	0.008	0.001	0.014	0.018	0.024	0.026	0.034	0.040	0.041	0.068	0.056	0.232	-	0.225	0.198	0.108	0.090	0.045	0.096
M25	0.001	0.001	0.001	0.002	0.003	0.017	0.012	0.004	0.031	0.024	0.034	0.014	0.031	0.068	0.052	0.038	0.069	0.212	0.225	-	0.233	0.135	0.164	0.121	0.108
M15	0.000	0.000	0.000	0.000	0.000	0.000	0.002	0.000	0.000	0.002	0.000	0.006	0.009	0.009	0.017	0.040	0.034	0.170	0.198	0.233	-	0.194	0.168	0.141	0.091
M10	0.000	0.000	0.000	0.000	0.001	0.007	0.007	0.001	0.013	0.013	0.016	0.011	0.016	0.032	0.016	0.009	0.023	0.157	0.108	0.135	0.194	-	0.248	0.191	0.172
M14	0.000	0.000	0.000	0.000	0.000	0.001	0.001	0.000	0.002	0.002	0.002	0.004	0.004	0.012	0.007	0.012	0.003	0.143	0.090	0.164	0.168	0.248	-	0.175	0.164
M37	0.000	0.000	0.000	0.000	0.000	0.001	0.000	0.000	0.002	0.001	0.003	0.002	0.003	0.008	0.007	0.014	0.005	0.102	0.045	0.121	0.141	0.191	0.175	-	0.220
M28	0.000	0.000	0.000	0.000	0.000	0.000	0.001	0.000	0.000	0.000	0.001	0.000	0.002	0.003	0.005	0.008	0.008	0.000	0.096	0.108	0.091	0.172	0.164	0.220	
M41	0.001	0.000	0.000	0.001	0.002	0.007	0.006	0.002	0.013	0.013	0.017	0.011	0.014	0.027	0.015	0.015	0.019	0.116	0.026	0.071	0.133	0.103	0.134	0.181	0.229
M47	0.000	0.000	0.000	0.000	0.000	0.000	0.000	0.000	0.000	0.000	0.000	0.000	0.000	0.000	0.002	0.004	0.001	0.023	0.024	0.079	0.024	0.115	0.072	0.133	0.171
M6	0.000	0.000	0.000	0.000	0.000	0.000	0.001	0.002	0.001	0.001	0.000	0.002	0.004	0.005	0.016	0.027	0.018	0.037	0.103	0.115	0.065	0.157	0.135	0.182	0.184
M38	0.000	0.000	0.000	0.000	0.001	0.003	0.002	0.001	0.004	0.004	0.008	0.005	0.006	0.014	0.007	0.010	0.008	0.086	0.006	0.058	0.097	0.077	0.071	0.101	0.192
M31	0.000	0.000	0.000	0.000	0.000	0.000	0.000	0.000	0.000	0.000	0.000	0.000	0.000	0.000	0.001	0.002	0.000	0.020	0.013	0.044	0.025	0.080	0.039	0.062	0.122
M46	0.000	0.000	0.000	0.000	0.000	0.000	0.000	0.000	0.000	0.000	0.000	0.000	0.000	0.000	0.000	0.000	0.000	0.014	0.002	0.033	0.009	0.038	0.017	0.052	0.087
M16	0.000	0.000	0.000	0.000	0.000	0.001	0.001	0.000	0.002	0.002	0.002	0.002	0.002	0.004	0.001	0.001	0.001	0.056	0.003	0.028	0.038	0.008	0.002	0.083	0.142
M13	0.000	0.000	0.000	0.000	0.000	0.001	0.001	0.000	0.001	0.002	0.001	0.002	0.001	0.003	0.001	0.001	0.001	0.045	0.001	0.017	0.029	0.003	0.001	0.061	0.117
M39	0.000	0.000	0.000	0.000	0.000	0.000	0.000	0.000	0.000	0.000	0.000	0.000	0.000	0.001	0.000	0.001	0.000	0.005	0.007	0.022	0.007	0.033	0.017	0.017	0.036
M42	0.000	0.000	0.000	0.000	0.000	0.002	0.001	0.000	0.003	0.003	0.004	0.002	0.003	0.007	0.002	0.002	0.003	0.042	0.002	0.016	0.033	0.013	0.021	0.029	0.092
M35	0.000	0.000	0.000	0.000	0.000	0.000	0.000	0.000	0.000	0.000	0.000	0.000	0.000	0.000	0.000	0.001	0.000	0.011	0.001	0.016	0.004	0.016	0.003	0.005	0.043
M40	0.000	0.000	0.000	0.000	0.000	0.000	0.000	0.000	0.000	0.000	0.000	0.000	0.000	0.000	0.000	0.000	0.000	0.002	0.002	0.011	0.001	0.014	0.004	0.004	0.015
M9	0.000	0.000	0.000	0.000	0.000	0.000	0.000	0.000	0.000	0.001	0.001	0.000	0.000	0.001	0.000	0.000	0.000	0.012	0.000	0.002	0.005	0.000	0.001	0.008	0.028
M8	0.000	0.000	0.000	0.000	0.000	0.000	0.000	0.000	0.000	0.001	0.001	0.000	0.000	0.001	0.000	0.000	0.000	0.012	0.000	0.002	0.005	0.000	0.001	0.008	0.028
M34	0.000	0.000	0.000	0.000	0.000	0.000	0.000	0.000	0.000	0.000	0.000	0.000	0.000	0.001	0.000	0.000	0.000	0.007	0.000	0.003	0.005	0.004	0.002	0.001	0.022
M5	0.000	0.000	0.000	0.000	0.000	0.000	0.000	0.000	0.000	0.000	0.000	0.000	0.000	0.000	0.000	0.000	0.000	0.004	0.000	0.000	0.003	0.000	0.001	0.001	0.011
M36	0.000	0.000	0.000	0.000	0.000	0.000	0.000	0.000	0.000	0.000	0.000	0.000	0.000	0.000	0.000	0.000	0.000	0.002	0.000	0.004	0.000	0.002	0.000	0.001	0.010
M11	0.000	0.000	0.000	0.000	0.000	0.000	0.000	0.000	0.000	0.000	0.000	0.000	0.000	0.001	0.000	0.000	0.000	0.006	0.000	0.001	0.002	0.000	0.000	0.002	0.012
M44	0.000	0.000	0.000	0.000	0.000	0.000	0.000	0.000	0.000	0.000	0.000	0.000	0.000	0.000	0.000	0.000	0.000	0.003	0.000	0.000	0.002	0.000	0.000	0.000	0.006
M48	0.000	0.000	0.000	0.000	0.000	0.000	0.000	0.000	0.000	0.000	0.000	0.000	0.000	0.000	0.000	0.000	0.000	0.002	0.000	0.000	0.001	0.000	0.000	0.000	0.004
M50	0.000	0.000	0.000	0.000	0.000	0.000	0.000	0.000	0.001	0.001	0.001	0.001	0.000	0.001	0.000	0.000	0.000	0.006	0.000	0.001	0.004	0.000	0.001	0.001	0.010
M49	0.000	0.000	0.000	0.000	0.000	0.000	0.000	0.000	0.000	0.000	0.000	0.000	0.000	0.000	0.000	0.000	0.000	0.000	0.000	0.000	0.000	0.000	0.000	0.000	0.000
M45	0.000	0.000	0.000	0.000	0.000	0.000	0.000	0.000	0.000	0.000	0.000	0.000	0.000	0.001	0.000	0.000	0.000	0.003	0.000	0.000	0.002	0.000	0.000	0.001	0.005
M30	0.000	0.000	0.000	0.000	0.000	0.000	0.000	0.000	0.000	0.000	0.000	0.000	0.000	0.000	0.000	0.000	0.000	0.000	0.000	0.000	0.000	0.000	0.000	0.000	0.000
M18	0.027	0.033	0.039	0.032	0.038	0.024	0.028	0.051	0.025	0.022	0.030	0.039	0.047	0.038	0.070	0.092	0.069	0.035	0.124	0.148	0.064	0.161	0.107	0.109	0.061

Table A.2: This table shows the resulting p-values comparing all the methods in table 8, which allows us to study the statistical significance of the results, as detailed in the Discussion section. The methods are sorted by the average harmonic score obtained from the 12 artificially created datasets detailed in the main paper.

	M41	M47	M6	M38	M31	M46	M16	M13	M39	M42	M35	M40	M9	M8	M34	M5	M36	M11	M44	M48	M50	M49	M45	M30	M18
M4	0.001	0.000	0.000	0.000	0.000	0.000	0.000	0.000	0.000	0.000	0.000	0.000	0.000	0.000	0.000	0.000	0.000	0.000	0.000	0.000	0.000	0.000	0.000	0.000	0.027
M17	0.000	0.000	0.000	0.000	0.000	0.000	0.000	0.000	0.000	0.000	0.000	0.000	0.000	0.000	0.000	0.000	0.000	0.000	0.000	0.000	0.000	0.000	0.000	0.000	0.033
M7	0.000	0.000	0.000	0.000	0.000	0.000	0.000	0.000	0.000	0.000	0.000	0.000	0.000	0.000	0.000	0.000	0.000	0.000	0.000	0.000	0.000	0.000	0.000	0.000	0.039
M12	0.001	0.000	0.000	0.000	0.000	0.000	0.000	0.000	0.000	0.000	0.000	0.000	0.000	0.000	0.000	0.000	0.000	0.000	0.000	0.000	0.000	0.000	0.000	0.000	0.032
M26	0.002	0.000	0.000	0.001	0.000	0.000	0.000	0.000	0.000	0.000	0.000	0.000	0.000	0.000	0.000	0.000	0.000	0.000	0.000	0.000	0.000	0.000	0.000	0.000	0.038
M3	0.007	0.000	0.000	0.003	0.000	0.000	0.001	0.001	0.000	0.002	0.000	0.000	0.000	0.000	0.000	0.000	0.000	0.000	0.000	0.000	0.000	0.000	0.000	0.000	0.024
M33	0.006	0.000	0.001	0.002	0.000	0.000	0.001	0.001	0.000	0.001	0.000	0.000	0.000	0.000	0.000	0.000	0.000	0.000	0.000	0.000	0.000	0.000	0.000	0.000	0.028
M27	0.002	0.000	0.002	0.001	0.000	0.000	0.000	0.000	0.000	0.000	0.000	0.000	0.000	0.000	0.000	0.000	0.000	0.000	0.000	0.000	0.000	0.000	0.000	0.000	0.051
M32	0.013	0.000	0.001	0.004	0.000	0.000	0.002	0.001	0.000	0.003	0.000	0.000	0.000	0.000	0.000	0.000	0.000	0.000	0.000	0.000	0.001	0.000	0.000	0.000	0.025
M22	0.013	0.000	0.001	0.004	0.000	0.000	0.002	0.002	0.000	0.003	0.000	0.000	0.001	0.001	0.000	0.000	0.000	0.000	0.000	0.000	0.001	0.000	0.000	0.000	0.022
M2	0.017	0.000	0.000	0.008	0.000	0.000	0.002	0.001	0.000	0.004	0.000	0.000	0.001	0.001	0.000	0.000	0.000	0.000	0.000	0.000	0.001	0.000	0.000	0.000	0.030
M20	0.011	0.000	0.002	0.005	0.000	0.000	0.002	0.002	0.000	0.002	0.000	0.000	0.000	0.000	0.000	0.000	0.000	0.000	0.000	0.000	0.001	0.000	0.000	0.000	0.039
M19	0.014	0.000	0.004	0.006	0.000	0.000	0.002	0.001	0.000	0.003	0.000	0.000	0.000	0.000	0.000	0.000	0.000	0.000	0.000	0.000	0.000	0.000	0.000	0.000	0.047
M1	0.027	0.000	0.005	0.014	0.000	0.000	0.004	0.003	0.001	0.007	0.000	0.000	0.001	0.001	0.001	0.000	0.000	0.001	0.000	0.000	0.001	0.000	0.001	0.000	0.038
M23	0.015	0.002	0.016	0.007	0.001	0.000	0.001	0.001	0.000	0.002	0.000	0.000	0.000	0.000	0.000	0.000	0.000	0.000	0.000	0.000	0.000	0.000	0.000	0.000	0.070
M21	0.015	0.004	0.027	0.010	0.002	0.000	0.001	0.001	0.001	0.002	0.001	0.000	0.000	0.000	0.000	0.000	0.000	0.000	0.000	0.000	0.000	0.000	0.000	0.000	0.092
M24	0.019	0.001	0.018	0.008	0.000	0.000	0.001	0.001	0.000	0.003	0.000	0.000	0.000	0.000	0.000	0.000	0.000	0.000	0.000	0.000	0.000	0.000	0.000	0.000	0.069
M29	0.116	0.023	0.037	0.086	0.020	0.014	0.056	0.045	0.005	0.042	0.011	0.002	0.012	0.012	0.007	0.004	0.002	0.006	0.003	0.002	0.006	0.000	0.003	0.000	0.035
M43	0.026	0.024	0.103	0.006	0.013	0.002	0.003	0.001	0.007	0.002	0.001	0.002	0.000	0.000	0.000	0.000	0.000	0.000	0.000	0.000	0.000	0.000	0.000	0.000	0.124
M25	0.071	0.079	0.115	0.058	0.044	0.033	0.028	0.017	0.022	0.016	0.016	0.011	0.002	0.002	0.003	0.000	0.004	0.001	0.000	0.000	0.001	0.000	0.000	0.000	0.148
M15	0.133	0.024	0.065	0.097	0.025	0.009	0.038	0.029	0.007	0.033	0.004	0.001	0.005	0.005	0.005	0.003	0.000	0.002	0.002	0.001	0.004	0.000	0.002	0.000	0.064
M10	0.103	0.115	0.157	0.077	0.080	0.038	0.008	0.003	0.033	0.013	0.016	0.014	0.000	0.000	0.004	0.000	0.002	0.000	0.000	0.000	0.000	0.000	0.000	0.000	0.161
M14	0.134	0.072	0.135	0.071	0.039	0.017	0.002	0.001	0.017	0.021	0.003	0.004	0.001	0.001	0.002	0.001	0.000	0.000	0.000	0.000	0.001	0.000	0.000	0.000	0.107
M37	0.181	0.133	0.182	0.101	0.062	0.052	0.083	0.061	0.017	0.029	0.005	0.004	0.008	0.008	0.001	0.001	0.001	0.002	0.000	0.000	0.001	0.000	0.001	0.000	0.109
M28	0.229	0.171	0.184	0.192	0.122	0.087	0.142	0.117	0.036	0.092	0.043	0.015	0.028	0.028	0.022	0.011	0.010	0.012	0.006	0.004	0.010	0.000	0.005	0.000	0.061
M41	-	0.230	0.229	0.157	0.173	0.154	0.114	0.077	0.081	0.005	0.041	0.039	0.001	0.001	0.007	0.000	0.012	0.000	0.000	0.000	0.000	0.000	0.000	0.000	0.238
M47	0.230	-	0.232	0.224	0.145	0.101	0.159	0.126	0.023	0.083	0.013	0.003	0.023	0.023	0.011	0.008	0.002	0.007	0.004	0.003	0.008	0.000	0.004	0.000	0.070
M6	0.229	0.232	-	0.247	0.212	0.209	0.208	0.186	0.094	0.146	0.083	0.042	0.084	0.084	0.059	0.057	0.022	0.044	0.033	0.025	0.037	0.006	0.024	0.000	0.025
M38	0.157	0.224	0.247	-	0.215	0.195	0.164	0.117	0.090	0.039	0.040	0.040	0.007	0.007	0.008	0.003	0.009	0.001	0.000	0.000	0.001	0.000	0.000	0.000	0.215
M31	0.173	0.145	0.212	0.215	-	0.227	0.220	0.180	0.065	0.111	0.031	0.013	0.038	0.038	0.013	0.010	0.005	0.011	0.005	0.004	0.010	0.000	0.004	0.000	0.090
M46	0.154	0.101	0.209	0.195	0.227	-	0.230	0.179	0.088	0.111	0.050	0.024	0.017	0.017	0.024	0.004	0.008	0.004	0.002	0.001	0.005	0.000	0.002	0.000	0.120
M16	0.114	0.159	0.208	0.164	0.220	0.230	-	0.034	0.133	0.097	0.073	0.063	0.003	0.003	0.027	0.005	0.011	0.000	0.001	0.000	0.003	0.000	0.001	0.000	0.175
M13	0.077	0.126	0.186	0.117	0.180	0.179	0.034	-	0.163	0.130	0.105	0.086	0.004	0.004	0.043	0.007	0.021	0.000	0.001	0.000	0.003	0.000	0.001	0.000	0.185
M39	0.081	0.023	0.094	0.090	0.065	0.088	0.133	0.163	-	0.248	0.169	0.035	0.139	0.139	0.050	0.086	0.011	0.056	0.029	0.022	0.034	0.003	0.020	0.000	0.071
M42	0.005	0.083	0.146	0.039	0.111	0.111	0.097	0.130	0.248	-	0.201	0.155	0.079	0.079	0.087	0.049	0.066	0.006	0.002	0.002	0.003	0.002	0.001	0.000	0.266
M35	0.041	0.013	0.083	0.040	0.031	0.050	0.073	0.105	0.169	0.201	-	0.114	0.160	0.160	0.074	0.093	0.007	0.053	0.025	0.019	0.030	0.003	0.016	0.000	0.129
M40	0.039	0.003	0.042	0.040	0.013	0.024	0.063	0.086	0.035	0.155	0.114	-	0.220	0.220	0.183	0.153	0.060	0.097	0.054	0.040	0.055	0.005	0.034	0.000	0.098
M9	0.001	0.023	0.084	0.007	0.038	0.017	0.003	0.004	0.139	0.079	0.160	0.220	-		0.238	0.118	0.161	0.000	0.003	0.002	0.008	0.002	0.002	0.000	0.240
M8	0.001	0.023	0.084	0.007	0.038	0.017	0.003	0.004	0.139	0.079	0.160	0.220	-		0.238	0.118	0.161	0.000	0.003	0.002	0.008	0.002	0.002	0.000	0.240
M34	0.007	0.011	0.059	0.008	0.013	0.024	0.027	0.043	0.050	0.087	0.074	0.183	0.238	0.238	-	0.173	0.100	0.098	0.037	0.026	0.043	0.006	0.020	0.000	0.194
M5	0.000	0.008	0.057	0.003	0.010	0.004	0.005	0.007	0.086	0.049	0.093	0.153	0.118	0.118	0.173	-	0.236	0.097	0.021	0.011	0.022	0.012	0.005	0.000	0.251
M36	0.012	0.002	0.022	0.009	0.005	0.008	0.011	0.021	0.011	0.066	0.007	0.060	0.161	0.161	0.100	0.236	-	0.174	0.101	0.071	0.092	0.016	0.054	0.000	0.136
M11	0.000	0.007	0.044	0.001	0.011	0.004	0.000	0.000	0.056	0.006	0.053	0.097	0.000	0.000	0.098	0.097	0.174	-	0.075	0.033	0.046	0.030	0.013	0.000	0.275
M44	0.000	0.004	0.033	0.000	0.005	0.002	0.001	0.001	0.029	0.002	0.025	0.054	0.003	0.003	0.037	0.021	0.101	0.075	-	0.030	0.102	0.069	0.007	0.000	0.326
M48	0.000	0.003	0.025	0.000	0.004	0.001	0.000	0.000	0.022	0.002	0.019	0.040	0.002	0.002	0.026	0.011	0.071	0.033	0.030	-	0.242	0.139	0.042	0.000	0.323
M50	0.000	0.008	0.037	0.001	0.010	0.005	0.003	0.003	0.034	0.003	0.030	0.055	0.008	0.008	0.043	0.022	0.092	0.046	0.102	0.242	-	0.176	0.032	0.000	0.345
M49	0.000	0.000	0.006	0.000	0.000	0.000	0.000	0.000	0.003	0.002	0.003	0.005	0.002	0.002	0.006	0.012	0.016	0.030	0.069	0.139	0.176	-	0.236	0.000	0.287
M45	0.000	0.004	0.024	0.000	0.004	0.002	0.001	0.001	0.020	0.001	0.016	0.034	0.002	0.002	0.020	0.005	0.054	0.013	0.007	0.042	0.032	0.236	-	0.000	0.354
M30	0.000	0.000	0.000	0.000	0.000	0.000	0.000	0.000	0.000	0.000	0.000	0.000	0.000	0.000	0.000	0.000	0.000	0.000	0.000	0.000	0.000	0.000	0.000	-	0.409
M18	0.238	0.070	0.025	0.215	0.090	0.120	0.175	0.185	0.071	0.266	0.129	0.098	0.240	0.240	0.194	0.251	0.136	0.275	0.326	0.323	0.345	0.287	0.354	0.409	

Table A.3: This table shows the resulting p-values comparing all the string-based methods in table 8, which allows us to study the statistical significance of the results, as detailed in the Discussion section. The methods are sorted by the average harmonic score obtained from the 12 artificially created datasets detailed in the main paper.

	M4	M3	M2	M1	M6	M5
M4		0.000	0.000	0.000	0.000	0.000
M3	0.000		0.003	0.006	0.000	0.000
M2	0.000	0.003		0.078	0.000	0.000
M1	0.000	0.006	0.078		0.005	0.000
M6	0.000	0.000	0.000	0.005		0.057
M5	0.000	0.000	0.000	0.000	0.057	

Table A.4: This table shows the resulting p-values comparing all the ontology-based methods in table 8, which allows us to study the statistical significance of the results, as detailed in the Discussion section. The methods are sorted by the average harmonic score obtained from the 12 artificially created datasets detailed in the main paper.

	M17	M7	M12	M15	M10	M14	M16	M13	M9	M8	M11
M17		0.088	0.000	0.000	0.000	0.000	0.000	0.000	0.000	0.000	0.000
M7	0.088		0.041	0.000	0.000	0.000	0.000	0.000	0.000	0.000	0.000
M12	0.000	0.041		0.000	0.000	0.000	0.000	0.000	0.000	0.000	0.000
M15	0.000	0.000	0.000		0.194	0.168	0.038	0.029	0.005	0.005	0.002
M10	0.000	0.000	0.000	0.194		0.248	0.008	0.003	0.000	0.000	0.000
M14	0.000	0.000	0.000	0.168	0.248		0.002	0.001	0.001	0.001	0.000
M16	0.000	0.000	0.000	0.038	0.008	0.002		0.034	0.003	0.003	0.000
M13	0.000	0.000	0.000	0.029	0.003	0.001	0.034		0.004	0.004	0.000
M9	0.000	0.000	0.000	0.005	0.000	0.001	0.003	0.004			0.000
M8	0.000	0.000	0.000	0.005	0.000	0.001	0.003	0.004			0.000
M11	0.000	0.000	0.000	0.002	0.000	0.000	0.000	0.000	0.000	0.000	

Table A.5: This table shows the resulting p-values comparing all the embedding-based methods in table 8, which allows us to study the statistical significance of the results, as detailed in the Discussion section. The methods are sorted by the average harmonic score obtained from the 12 artificially created datasets detailed in the main paper.

	M26	M33	M27	M32	M22	M20	M19	M23	M21	M24	M29	M25	M28	M31	M30	M18
M26		0.032	0.007	0.022	0.023	0.006	0.006	0.005	0.003	0.000	0.000	0.003	0.000	0.000	0.000	0.038
M33	0.032		0.214	0.150	0.139	0.072	0.049	0.040	0.031	0.009	0.007	0.012	0.001	0.000	0.000	0.028
M27	0.007	0.214		0.182	0.178	0.079	0.037	0.018	0.008	0.000	0.006	0.004	0.000	0.000	0.000	0.051
M32	0.022	0.150	0.182		0.248	0.169	0.088	0.050	0.057	0.017	0.004	0.031	0.000	0.000	0.000	0.025
M22	0.023	0.139	0.178	0.248		0.137	0.045	0.041	0.048	0.013	0.002	0.024	0.000	0.000	0.000	0.022
M20	0.006	0.072	0.079	0.169	0.137		0.090	0.072	0.044	0.018	0.006	0.014	0.000	0.000	0.000	0.039
M19	0.006	0.049	0.037	0.088	0.045	0.090		0.095	0.083	0.030	0.027	0.031	0.002	0.000	0.000	0.047
M23	0.005	0.040	0.018	0.050	0.041	0.072	0.095		0.147	0.111	0.067	0.052	0.005	0.001	0.000	0.070
M21	0.003	0.031	0.008	0.057	0.048	0.044	0.083	0.147		0.199	0.098	0.038	0.008	0.002	0.000	0.092
M24	0.000	0.009	0.000	0.017	0.013	0.018	0.030	0.111	0.199		0.107	0.069	0.008	0.000	0.000	0.069
M29	0.000	0.007	0.006	0.004	0.002	0.006	0.027	0.067	0.098	0.107		0.212	0.000	0.020	0.000	0.035
M25	0.003	0.012	0.004	0.031	0.024	0.014	0.031	0.052	0.038	0.069	0.212		0.108	0.044	0.000	0.148
M28	0.000	0.001	0.000	0.000	0.000	0.000	0.002	0.005	0.008	0.008	0.000	0.108		0.122	0.000	0.061
M31	0.000	0.000	0.000	0.000	0.000	0.000	0.000	0.001	0.002	0.000	0.020	0.044	0.122		0.000	0.090
M30	0.000	0.000	0.000	0.000	0.000	0.000	0.000	0.000	0.000	0.000	0.000	0.000	0.000	0.000		0.409
M18	0.038	0.028	0.051	0.025	0.022	0.039	0.047	0.070	0.092	0.069	0.035	0.148	0.061	0.090	0.409	

Table A.6: This table shows the resulting p-values comparing all the BERT-based methods in table 8, which allows us to study the statistical significance of the results, as detailed in the Discussion section. The methods are sorted by the average harmonic score obtained from the 12 artificially created datasets detailed in the main paper.

	M43	M37	M41	M47	M38	M46	M39	M42	M35	M40	M34	M36	M48	M44	M50	M45	M49
M43		0.045	0.026	0.024	0.006	0.002	0.007	0.002	0.001	0.002	0.000	0.000	0.000	0.000	0.000	0.000	0.000
M37	0.045		0.181	0.133	0.101	0.052	0.017	0.029	0.005	0.004	0.001	0.001	0.000	0.000	0.001	0.000	0.001
M41	0.026	0.181		0.230	0.157	0.154	0.081	0.005	0.041	0.039	0.007	0.012	0.000	0.000	0.000	0.000	0.000
M47	0.024	0.133	0.230		0.224	0.101	0.023	0.083	0.013	0.003	0.011	0.002	0.004	0.003	0.008	0.000	0.004
M38	0.006	0.101	0.157	0.224		0.195	0.090	0.039	0.040	0.040	0.008	0.009	0.000	0.000	0.001	0.000	0.000
M46	0.002	0.052	0.154	0.101	0.195		0.088	0.111	0.050	0.024	0.024	0.008	0.002	0.001	0.005	0.000	0.002
M39	0.007	0.017	0.081	0.023	0.090	0.088		0.248	0.169	0.035	0.050	0.011	0.029	0.022	0.034	0.003	0.020
M42	0.002	0.029	0.005	0.083	0.039	0.111	0.248		0.201	0.155	0.087	0.066	0.002	0.002	0.003	0.002	0.001
M35	0.001	0.005	0.041	0.013	0.040	0.050	0.169	0.201		0.114	0.074	0.007	0.025	0.019	0.030	0.003	0.016
M40	0.002	0.004	0.039	0.003	0.040	0.024	0.035	0.155	0.114		0.183	0.060	0.054	0.040	0.055	0.005	0.034
M34	0.000	0.001	0.007	0.011	0.008	0.024	0.050	0.087	0.074	0.183		0.100	0.037	0.026	0.043	0.006	0.020
M36	0.000	0.001	0.012	0.002	0.009	0.008	0.011	0.066	0.007	0.060	0.100		0.101	0.071	0.092	0.016	0.054
M48	0.000	0.000	0.000	0.004	0.000	0.002	0.029	0.002	0.025	0.054	0.037	0.101		0.030	0.102	0.069	0.007
M44	0.000	0.000	0.000	0.003	0.000	0.001	0.022	0.002	0.019	0.040	0.026	0.071	0.030		0.242	0.139	0.042
M50	0.000	0.001	0.000	0.008	0.001	0.005	0.034	0.003	0.030	0.055	0.043	0.092	0.102	0.242		0.176	0.032
M45	0.000	0.000	0.000	0.000	0.000	0.000	0.003	0.002	0.003	0.005	0.006	0.016	0.069	0.139	0.176		0.236
M49	0.000	0.001	0.000	0.004	0.000	0.002	0.020	0.001	0.016	0.034	0.020	0.054	0.007	0.042	0.032	0.236	
