# Supplementary material for: A reproducible experimental survey on biomedical sentence similarity: A string-based method sets the state of the art
Source: PLoS One. 2022 Nov 21;17(11):e0276539. doi: 10.1371/journal.pone.0276539 (PMC9678326; doi:10.1371/journal.pone.0276539)
Supplement: S2 Appendix — We provide all the pre-processing raw output tables for the experiments evaluated herein as supplementary material. (PDF) [file pone.0276539.s002.pdf]

Appendix B
Pre-processing raw output files

This appendix shows all the pre-processing raw output tables for the experiments evaluated herein (see figure 2), as detailed in the workflow of our experiments.

Table A.1: Part 1 of 5. Pearson (r), Spearman (*ho*) and Harmonic score (h) obtained by the String-based similarity methods evaluated herein.

	BIOSSES			MedSTS			CTR			Avg
	r	ρ	h	r	ρ	h	r	ρ	h	Avg
LiBlock_tok.corenlp_lc_sw.nltk_cf.default_ner.ctakes	0.820	0.828	0.824	0.769	0.710	0.739	0.793	0.808	0.800	0.788
LiBlock_tok.corenlp_lc_sw.nltk_cf.biosses_ner.ctakes	0.820	0.829	0.825	0.769	0.710	0.738	0.792	0.807	0.799	0.787
LiBlock_tok.corenlp_lc_sw.biosses_cf.default_ner.ctakes	0.817	0.828	0.823	0.770	0.711	0.739	0.792	0.807	0.799	0.787
LiBlock_tok.corenlp_lc_sw.biosses_cf.blagec_ner.ctakes	0.818	0.827	0.822	0.769	0.711	0.739	0.792	0.807	0.799	0.787
LiBlock_tok.corenlp_lc_sw.biosses_cf.biosses_ner.ctakes	0.817	0.827	0.822	0.769	0.711	0.739	0.792	0.807	0.799	0.787
LiBlock_tok.corenlp_lc_sw.biosses_cf.none_ner.ctakes	0.818	0.828	0.823	0.768	0.707	0.737	0.792	0.807	0.800	0.786
LiBlock_tok.whitesp_lc_sw.nltk_cf.default_ner.ctakes	0.812	0.827	0.820	0.763	0.705	0.733	0.792	0.800	0.796	0.783
LiBlock_tok.whitesp_lc_sw.nltk_cf.biosses_ner.ctakes	0.811	0.827	0.819	0.764	0.705	0.733	0.792	0.800	0.796	0.783
LiBlock_tok.bioc_lc_sw.nltk_cf.default_ner.ctakes	0.812	0.826	0.819	0.763	0.704	0.732	0.793	0.801	0.797	0.783
LiBlock_tok.whitesp_lc_sw.biosses_cf.default_ner.ctakes	0.809	0.826	0.817	0.764	0.706	0.734	0.792	0.800	0.796	0.782
LiBlock_tok.bioc_lc_sw.nltk_cf.biosses_ner.ctakes	0.811	0.826	0.819	0.762	0.703	0.732	0.792	0.801	0.796	0.782
LiBlock_tok.whitesp_lc_sw.biosses_cf.biosses_ner.ctakes	0.808	0.825	0.817	0.764	0.706	0.734	0.792	0.800	0.796	0.782
LiBlock_tok.bioc_lc_sw.biosses_cf.default_ner.ctakes	0.810	0.825	0.817	0.763	0.704	0.732	0.792	0.801	0.796	0.782
LiBlock_tok.bioc_lc_sw.biosses_cf.none_ner.ctakes	0.809	0.825	0.817	0.762	0.703	0.731	0.793	0.801	0.797	0.782
LiBlock_tok.bioc_lc_sw.biosses_cf.blagec_ner.ctakes	0.809	0.824	0.817	0.763	0.704	0.732	0.792	0.800	0.796	0.782
LiBlock_tok.bioc_lc_sw.biosses_cf.biosses_ner.ctakes	0.809	0.825	0.817	0.763	0.704	0.732	0.792	0.800	0.796	0.782
LiBlock_tok.whitesp_lc_sw.biosses_cf.blagec_ner.ctakes	0.803	0.819	0.811	0.764	0.706	0.734	0.792	0.800	0.796	0.780
LiBlock_tok.corenlp_lc_sw.none_cf.default_ner.ctakes	0.800	0.826	0.812	0.762	0.710	0.735	0.780	0.783	0.781	0.776
LiBlock_tok.corenlp_lc_sw.none_cf.biosses_ner.ctakes	0.800	0.826	0.813	0.762	0.709	0.735	0.779	0.783	0.781	0.776
LiBlock_tok.corenlp_notlc_sw.nltk_cf.default_ner.ctakes	0.808	0.815	0.811	0.754	0.694	0.723	0.788	0.793	0.791	0.775
LiBlock_tok.corenlp_notlc_sw.nltk_cf.biosses_ner.ctakes	0.808	0.815	0.812	0.754	0.693	0.722	0.787	0.793	0.790	0.775
LiBlock_tok.corenlp_notlc_sw.biosses_cf.default_ner.ctakes	0.805	0.813	0.809	0.754	0.695	0.723	0.787	0.793	0.790	0.774
LiBlock_tok.corenlp_notlc_sw.biosses_cf.none_ner.ctakes	0.806	0.816	0.811	0.753	0.692	0.721	0.787	0.793	0.790	0.774
LiBlock_tok.corenlp_notlc_sw.biosses_cf.blagec_ner.ctakes	0.806	0.813	0.809	0.754	0.694	0.723	0.787	0.792	0.790	0.774
LiBlock_tok.corenlp_notlc_sw.biosses_cf.biosses_ner.ctakes	0.806	0.813	0.809	0.754	0.694	0.723	0.787	0.792	0.789	0.774
LiBlock_tok.whitesp_lc_sw.biosses_cf.none_ner.ctakes	0.789	0.806	0.797	0.753	0.698	0.725	0.793	0.801	0.797	0.773
LiBlock_tok.corenlp_lc_sw.nltk_cf.blagec_ner.ctakes	0.813	0.822	0.817	0.768	0.707	0.736	0.772	0.757	0.764	0.773
LiBlock_tok.whitesp_notlc_sw.nltk_cf.default_ner.ctakes	0.807	0.819	0.813	0.749	0.689	0.718	0.787	0.788	0.787	0.773
LiBlock_tok.whitesp_notlc_sw.nltk_cf.biosses_ner.ctakes	0.807	0.818	0.812	0.749	0.689	0.718	0.787	0.787	0.787	0.772
LiBlock_tok.bioc_notlc_sw.nltk_cf.default_ner.ctakes	0.807	0.818	0.812	0.748	0.687	0.716	0.787	0.788	0.788	0.772
LiBlock_tok.whitesp_notlc_sw.biosses_cf.default_ner.ctakes	0.804	0.818	0.811	0.750	0.690	0.719	0.786	0.787	0.786	0.772
LiBlock_tok.whitesp_notlc_sw.biosses_cf.biosses_ner.ctakes	0.804	0.817	0.810	0.750	0.690	0.719	0.786	0.787	0.786	0.772
LiBlock_tok.bioc_notlc_sw.nltk_cf.biosses_ner.ctakes	0.807	0.817	0.812	0.748	0.686	0.716	0.787	0.788	0.787	0.772
LiBlock_tok.whitesp_lc_sw.none_cf.biosses_ner.ctakes	0.791	0.823	0.807	0.758	0.705	0.731	0.779	0.776	0.777	0.771
LiBlock_tok.bioc_notlc_sw.biosses_cf.default_ner.ctakes	0.804	0.817	0.811	0.748	0.688	0.717	0.786	0.787	0.787	0.771
LiBlock_tok.bioc_notlc_sw.biosses_cf.blagec_ner.ctakes	0.804	0.816	0.810	0.748	0.687	0.716	0.787	0.788	0.787	0.771
BlockDist_tok.whitesp_lc_sw.nltk_cf.biosses_ner.none	0.798	0.818	0.808	0.731	0.683	0.706	0.797	0.801	0.799	0.771
LiBlock_tok.bioc_notlc_sw.biosses_cf.none_ner.ctakes	0.804	0.816	0.810	0.748	0.686	0.716	0.787	0.788	0.788	0.771
LiBlock_tok.bioc_notlc_sw.biosses_cf.biosses_ner.ctakes	0.804	0.816	0.810	0.748	0.687	0.716	0.786	0.787	0.787	0.771
BlockDist_tok.corenlp_lc_sw.nltk_cf.default_ner.none	0.788	0.803	0.795	0.740	0.691	0.715	0.795	0.809	0.802	0.771
BlockDist_tok.corenlp_lc_sw.nltk_cf.biosses_ner.none	0.788	0.804	0.796	0.740	0.691	0.715	0.794	0.808	0.801	0.770
LiBlock_tok.bioc_lc_sw.none_cf.biosses_ner.ctakes	0.791	0.822	0.807	0.754	0.701	0.727	0.778	0.775	0.777	0.770
LiBlock_tok.whitesp_lc_sw.none_cf.default_ner.ctakes	0.787	0.816	0.802	0.757	0.706	0.731	0.779	0.776	0.778	0.770
BlockDist_tok.corenlp_lc_sw.biosses_cf.default_ner.none	0.782	0.801	0.792	0.741	0.692	0.716	0.794	0.807	0.801	0.770
LiBlock_tok.whitesp_notlc_sw.biosses_cf.blagec_ner.ctakes	0.797	0.808	0.803	0.750	0.690	0.719	0.787	0.787	0.787	0.769
LiBlock_tok.corenlp_lc_sw.none_cf.blagec_ner.ctakes	0.795	0.821	0.808	0.761	0.707	0.733	0.766	0.769	0.767	0.769
BlockDist_tok.whitesp_lc_sw.biosses_cf.biosses_ner.none	0.794	0.814	0.804	0.732	0.683	0.707	0.795	0.799	0.797	0.769
LiBlock_tok.whitesp_lc_sw.nltk_cf.blagec_ner.ctakes	0.806	0.822	0.814	0.763	0.703	0.732	0.771	0.752	0.761	0.769
BlockDist_tok.corenlp_lc_sw.biosses_cf.blagec_ner.none	0.783	0.803	0.792	0.741	0.691	0.715	0.793	0.806	0.800	0.769
BlockDist_tok.corenlp_lc_sw.biosses_cf.biosses_ner.none	0.782	0.802	0.792	0.741	0.691	0.715	0.793	0.807	0.800	0.769
LiBlock_tok.bioc_lc_sw.none_cf.default_ner.ctakes	0.788	0.816	0.802	0.754	0.702	0.727	0.779	0.776	0.777	0.769
LiBlock_tok.bioc_lc_sw.nltk_cf.blagec_ner.ctakes	0.804	0.816	0.810	0.761	0.700	0.729	0.773	0.756	0.765	0.768
BlockDist_tok.whitesp_lc_sw.nltk_cf.default_ner.none	0.788	0.808	0.798	0.730	0.683	0.706	0.797	0.802	0.799	0.768
LiBlock_tok.corenlp_notlc_sw.none_cf.default_ner.ctakes	0.788	0.815	0.801	0.750	0.699	0.723	0.777	0.777	0.777	0.767
LiBlock_tok.corenlp_notlc_sw.none_cf.biosses_ner.ctakes	0.788	0.815	0.801	0.750	0.698	0.723	0.776	0.777	0.777	0.767
BlockDist_tok.corenlp_lc_sw.biosses_cf.none_ner.none	0.781	0.803	0.792	0.736	0.682	0.708	0.793	0.807	0.800	0.767
BlockDist_tok.whitesp_lc_sw.biosses_cf.default_ner.none	0.783	0.804	0.794	0.732	0.684	0.707	0.795	0.800	0.797	0.766
LiBlock_tok.corenlp_lc_sw.none_cf.none_ner.ctakes	0.798	0.820	0.809	0.755	0.700	0.726	0.766	0.759	0.763	0.766
BlockDist_tok.bioc_lc_sw.nltk_cf.biosses_ner.none	0.799	0.816	0.808	0.714	0.668	0.690	0.797	0.801	0.799	0.766
LiBlock_tok.whitesp_lc_sw.none_cf.blagec_ner.ctakes	0.787	0.818	0.802	0.757	0.704	0.730	0.766	0.764	0.765	0.765
LiBlock_tok.bioc_lc_sw.none_cf.blagec_ner.ctakes	0.787	0.817	0.802	0.753	0.699	0.725	0.766	0.768	0.767	0.765
BlockDist_tok.bioc_lc_sw.biosses_cf.blagec_ner.none	0.794	0.813	0.804	0.715	0.668	0.691	0.796	0.799	0.798	0.764
BlockDist_tok.bioc_lc_sw.biosses_cf.biosses_ner.none	0.794	0.813	0.804	0.715	0.668	0.691	0.795	0.799	0.797	0.764
BlockDist_tok.whitesp_lc_sw.biosses_cf.blagec_ner.none	0.775	0.796	0.786	0.732	0.685	0.708	0.796	0.799	0.798	0.764
LiBlock_tok.whitesp_notlc_sw.biosses_cf.none_ner.ctakes	0.781	0.797	0.789	0.741	0.685	0.712	0.787	0.789	0.788	0.763
BlockDist_tok.bioc_lc_sw.nltk_cf.default_ner.none	0.789	0.807	0.798	0.715	0.669	0.691	0.797	0.802	0.799	0.763
LiBlock_tok.corenlp_lc_sw.nltk_cf.none_ner.ctakes	0.806	0.821	0.813	0.760	0.699	0.729	0.770	0.724	0.746	0.763
LiBlock_tok.whitesp_notlc_sw.none_cf.default_ner.ctakes	0.781	0.805	0.793	0.746	0.695	0.720	0.775	0.772	0.774	0.762
LiBlock_tok.whitesp_notlc_sw.none_cf.biosses_ner.ctakes	0.781	0.805	0.793	0.746	0.695	0.720	0.775	0.772	0.773	0.762
LiBlock_tok.corenlp_notlc_sw.nltk_cf.blagec_ner.ctakes	0.800	0.809	0.805	0.753	0.691	0.720	0.767	0.752	0.760	0.762
BlockDist_tok.bioc_lc_sw.biosses_cf.default_ner.none	0.784	0.803	0.793	0.716	0.670	0.692	0.795	0.800	0.797	0.761
LiBlock_tok.bioc_lc_sw.none_cf.none_ner.ctakes	0.787	0.809	0.798	0.746	0.691	0.718	0.771	0.763	0.767	0.761
LiBlock_tok.corenlp_notlc_sw.none_cf.none_ner.ctakes	0.791	0.819	0.805	0.744	0.690	0.716	0.764	0.758	0.761	0.761
LiBlock_tok.bioc_notlc_sw.none_cf.default_ner.ctakes	0.781	0.804	0.793	0.742	0.690	0.715	0.775	0.773	0.774	0.761
LiBlock_tok.bioc_notlc_sw.none_cf.biosses_ner.ctakes	0.781	0.805	0.793	0.742	0.690	0.715	0.775	0.772	0.773	0.760
LiBlock_tok.whitesp_notlc_sw.nltk_cf.blagec_ner.ctakes	0.801	0.812	0.806	0.748	0.687	0.716	0.766	0.749	0.757	0.760
BlockDist_tok.bioc_lc_sw.biosses_cf.none_ner.none	0.783	0.802	0.792	0.715	0.667	0.690	0.795	0.800	0.797	0.760
LiBlock_tok.bioc_notlc_sw.nltk_cf.blagec_ner.ctakes	0.799	0.810	0.804	0.746	0.684	0.714	0.768	0.754	0.761	0.760
LiBlock_tok.corenlp_notlc_sw.none_cf.blagec_ner.ctakes	0.783	0.808	0.795	0.749	0.696	0.722	0.762	0.761	0.762	0.760
LiBlock_tok.bioc_lc_sw.nltk_cf.none_ner.ctakes	0.802	0.813	0.807	0.751	0.691	0.720	0.776	0.729	0.752	0.760
LiBlock_tok.bioc_notlc_sw.none_cf.none_ner.ctakes	0.786	0.809	0.797	0.736	0.682	0.708	0.767	0.765	0.766	0.757
Jaccard_tok.whitesp_lc_sw.nltk_cf.biosses_ner.none	0.782	0.815	0.798	0.706	0.680	0.693	0.759	0.797	0.777	0.756
LiBlock_tok.whitesp_lc_sw.none_cf.none_ner.ctakes	0.766	0.802	0.784	0.746	0.695	0.719	0.769	0.758	0.764	0.756
LiBlock_tok.whitesp_notlc_sw.none_cf.blagec_ner.ctakes	0.776	0.800	0.788	0.746	0.694	0.719	0.761	0.759	0.760	0.756
LiBlock_tok.corenlp_notlc_sw.nltk_cf.none_ner.ctakes	0.799	0.820	0.809	0.748	0.685	0.715	0.763	0.720	0.741	0.755
LiBlock_tok.bioc_notlc_sw.nltk_cf.none_ner.ctakes	0.805	0.817	0.811	0.739	0.677	0.707	0.768	0.727	0.747	0.755
LiBlock_tok.bioc_notlc_sw.none_cf.blagec_ner.ctakes	0.777	0.799	0.788	0.742	0.688	0.714	0.762	0.763	0.763	0.755
Jaccard_tok.whitesp_lc_sw.biosses_cf.biosses_ner.none	0.777	0.812	0.794	0.708	0.680	0.694	0.757	0.796	0.776	0.755
Jaccard_tok.corenlp_lc_sw.nltk_cf.default_ner.none	0.773	0.804	0.788	0.716	0.690	0.703	0.739	0.804	0.770	0.754
LiBlock_tok.whitesp_lc_sw.nltk_cf.none_ner.ctakes	0.790	0.806	0.798	0.748	0.689	0.717	0.773	0.720	0.745	0.754
Jaccard_tok.whitesp_lc_sw.nltk_cf.default_ner.none	0.774	0.807	0.790	0.706	0.680	0.692	0.758	0.798	0.777	0.753
Jaccard_tok.corenlp_lc_sw.nltk_cf.biosses_ner.none	0.773	0.805	0.789	0.715	0.690	0.702	0.738	0.803	0.769	0.753
Jaccard_tok.corenlp_lc_sw.biosses_cf.default_ner.none	0.767	0.801	0.784	0.716	0.690	0.703	0.738	0.802	0.768	0.752
Jaccard_tok.corenlp_lc_sw.biosses_cf.blagec_ner.none	0.768	0.801	0.784	0.716	0.690	0.703	0.738	0.802	0.768	0.752
Jaccard_tok.whitesp_lc_sw.biosses_cf.default_ner.none	0.769	0.803	0.786	0.707	0.680	0.693	0.756	0.796	0.776	0.752
Jaccard_tok.bioc_lc_sw.nltk_cf.biosses_ner.none	0.783	0.814	0.798	0.692	0.665	0.678	0.759	0.797	0.777	0.751
Jaccard_tok.corenlp_lc_sw.biosses_cf.biosses_ner.none	0.767	0.801	0.784	0.716	0.690	0.703	0.736	0.801	0.767	0.751
Jaccard_tok.bioc_lc_sw.biosses_cf.blagec_ner.none	0.778	0.811	0.794	0.693	0.666	0.679	0.758	0.795	0.776	0.750
Jaccard_tok.bioc_lc_sw.biosses_cf.biosses_ner.none	0.779	0.811	0.794	0.693	0.666	0.679	0.757	0.796	0.776	0.750
Jaccard_tok.corenlp_lc_sw.biosses_cf.none_ner.none	0.764	0.803	0.783	0.713	0.683	0.698	0.737	0.802	0.768	0.750
LiBlock_tok.whitesp_notlc_sw.none_cf.none_ner.ctakes	0.759	0.793	0.776	0.736	0.686	0.710	0.765	0.758	0.762	0.749
Jaccard_tok.bioc_lc_sw.nltk_cf.default_ner.none	0.775	0.805	0.790	0.693	0.666	0.679	0.758	0.798	0.777	0.749
Jaccard_tok.whitesp_lc_sw.biosses_cf.blagec_ner.none	0.757	0.792	0.774	0.707	0.681	0.694	0.758	0.795	0.776	0.748
Jaccard_tok.bioc_lc_sw.biosses_cf.default_ner.none	0.770	0.802	0.786	0.693	0.666	0.680	0.756	0.796	0.776	0.747
Jaccard_tok.bioc_lc_sw.biosses_cf.none_ner.none	0.770	0.800	0.785	0.692	0.665	0.678	0.756	0.797	0.776	0.746

Table A.2: Part 2 of 5. Pearson (r), Spearman (*ho*) and Harmonic score (h) obtained by the String-based similarity methods evaluated herein.

	BIOSSES			MedSTS			CTR			Avg
	r	ρ	h	r	ρ	h	r	ρ	h	Avg
LiBlock_tok.whitesp_notlc_sw.nltk_cf.none_ner.ctakes	0.779	0.793	0.786	0.736	0.676	0.704	0.765	0.717	0.741	0.744
BlockDist_tok.whitesp_lc_sw.nltk_cf.blagec_ner.none	0.781	0.800	0.790	0.731	0.684	0.706	0.729	0.711	0.720	0.739
Qgram_tok.whitesp_lc_sw.nltk_cf.biosses_ner.none	0.752	0.773	0.763	0.701	0.674	0.687	0.763	0.766	0.764	0.738
Qgram_tok.corenlp_lc_sw.nltk_cf.default_ner.none	0.743	0.766	0.754	0.709	0.681	0.695	0.760	0.764	0.762	0.737
Qgram_tok.whitesp_lc_sw.biosses_cf.biosses_ner.none	0.750	0.771	0.760	0.701	0.674	0.687	0.761	0.763	0.762	0.737
Qgram_tok.whitesp_lc_sw.nltk_cf.default_ner.none	0.743	0.765	0.754	0.708	0.680	0.694	0.760	0.763	0.762	0.737
Qgram_tok.corenlp_lc_sw.biosses_cf.none_ner.none	0.742	0.767	0.754	0.709	0.678	0.693	0.759	0.763	0.761	0.736
Qgram_tok.corenlp_lc_sw.nltk_cf.biosses_ner.none	0.745	0.769	0.757	0.701	0.675	0.688	0.760	0.765	0.762	0.736
Qgram_tok.corenlp_lc_sw.biosses_cf.default_ner.none	0.741	0.764	0.752	0.710	0.681	0.695	0.759	0.761	0.760	0.736
Qgram_tok.whitesp_lc_sw.biosses_cf.default_ner.none	0.741	0.765	0.753	0.709	0.680	0.694	0.759	0.761	0.760	0.736
Qgram_tok.whitesp_lc_sw.biosses_cf.blagec_ner.none	0.743	0.768	0.755	0.701	0.674	0.687	0.762	0.764	0.763	0.735
Qgram_tok.whitesp_lc_sw.nltk_cf.blagec_ner.none	0.746	0.772	0.759	0.701	0.674	0.687	0.753	0.764	0.759	0.735
Qgram_tok.corenlp_lc_sw.biosses_cf.blagec_ner.none	0.743	0.767	0.755	0.702	0.676	0.688	0.760	0.762	0.761	0.735
Qgram_tok.corenlp_lc_sw.biosses_cf.biosses_ner.none	0.742	0.766	0.754	0.702	0.676	0.688	0.759	0.762	0.760	0.734
Qgram_tok.whitesp_lc_sw.none_cf.biosses_ner.none	0.725	0.760	0.742	0.711	0.697	0.704	0.742	0.772	0.757	0.734
Qgram_tok.bioc_lc_sw.nltk_cf.biosses_ner.none	0.753	0.774	0.763	0.685	0.658	0.671	0.763	0.766	0.764	0.733
BlockDist_tok.corenlp_lc_sw.nltk_cf.blagec_ner.none	0.761	0.769	0.765	0.737	0.685	0.710	0.733	0.714	0.723	0.733
OverlapCoeff_tok.corenlp_lc_sw.nltk_cf.default_ner.none	0.782	0.795	0.788	0.696	0.564	0.623	0.781	0.793	0.787	0.733
OverlapCoeff_tok.corenlp_lc_sw.nltk_cf.biosses_ner.none	0.781	0.795	0.788	0.695	0.564	0.623	0.780	0.792	0.786	0.732
Qgram_tok.corenlp_lc_sw.nltk_cf.blagec_ner.none	0.741	0.762	0.752	0.701	0.675	0.687	0.752	0.761	0.756	0.732
Qgram_tok.bioc_lc_sw.nltk_cf.default_ner.none	0.744	0.765	0.754	0.694	0.665	0.679	0.760	0.763	0.762	0.732
Qgram_tok.bioc_lc_sw.biosses_cf.blagec_ner.none	0.751	0.771	0.761	0.686	0.658	0.672	0.762	0.764	0.763	0.732
Qgram_tok.bioc_lc_sw.biosses_cf.biosses_ner.none	0.751	0.771	0.761	0.686	0.658	0.672	0.761	0.763	0.762	0.731
Qgram_tok.corenlp_lc_sw.none_cf.biosses_ner.none	0.716	0.755	0.735	0.711	0.698	0.705	0.740	0.769	0.755	0.731
Qgram_tok.whitesp_lc_sw.none_cf.default_ner.none	0.715	0.752	0.733	0.711	0.698	0.705	0.741	0.771	0.756	0.731
Qgram_tok.bioc_lc_sw.biosses_cf.none_ner.none	0.744	0.764	0.754	0.693	0.662	0.677	0.760	0.764	0.762	0.731
Qgram_tok.corenlp_lc_sw.none_cf.default_ner.none	0.715	0.752	0.733	0.711	0.698	0.705	0.741	0.771	0.756	0.731
Qgram_tok.bioc_lc_sw.biosses_cf.default_ner.none	0.741	0.765	0.753	0.695	0.665	0.679	0.759	0.761	0.760	0.731
OverlapCoeff_tok.corenlp_lc_sw.biosses_cf.default_ner.none	0.777	0.790	0.784	0.696	0.564	0.623	0.780	0.790	0.785	0.730
OverlapCoeff_tok.corenlp_lc_sw.biosses_cf.blagec_ner.none	0.777	0.791	0.784	0.695	0.564	0.623	0.779	0.790	0.785	0.730
BlockDist_tok.whitesp_lc_sw.biosses_cf.none_ner.none	0.706	0.743	0.724	0.692	0.648	0.669	0.795	0.800	0.797	0.730
OverlapCoeff_tok.corenlp_lc_sw.biosses_cf.biosses_ner.none	0.776	0.790	0.783	0.695	0.564	0.623	0.779	0.790	0.784	0.730
Qgram_tok.whitesp_lc_sw.biosses_cf.none_ner.none	0.726	0.749	0.737	0.705	0.676	0.690	0.760	0.764	0.762	0.730
Qgram_tok.whitesp_lc_sw.nltk_cf.none_ner.none	0.729	0.752	0.740	0.703	0.675	0.689	0.757	0.764	0.760	0.730
Qgram_tok.bioc_lc_sw.nltk_cf.blagec_ner.none	0.749	0.770	0.759	0.685	0.657	0.671	0.753	0.764	0.759	0.730
OverlapCoeff_tok.corenlp_lc_sw.biosses_cf.none_ner.none	0.777	0.790	0.783	0.693	0.559	0.619	0.779	0.792	0.785	0.729
Qgram_tok.whitesp_lc_sw.none_cf.blagec_ner.none	0.720	0.750	0.734	0.710	0.697	0.704	0.735	0.762	0.749	0.729
Qgram_tok.corenlp_lc_sw.nltk_cf.none_ner.none	0.733	0.752	0.742	0.700	0.673	0.686	0.753	0.762	0.758	0.729
Qgram_tok.bioc_lc_sw.none_cf.biosses_ner.none	0.726	0.760	0.743	0.691	0.679	0.685	0.742	0.772	0.757	0.728
Qgram_tok.corenlp_lc_sw.none_cf.blagec_ner.none	0.713	0.748	0.730	0.711	0.697	0.704	0.734	0.761	0.747	0.727
OverlapCoeff_tok.whitesp_lc_sw.nltk_cf.biosses_ner.none	0.776	0.798	0.787	0.686	0.556	0.614	0.774	0.785	0.780	0.727
Jaccard_tok.whitesp_lc_sw.nltk_cf.blagec_ner.none	0.762	0.796	0.779	0.705	0.680	0.692	0.705	0.712	0.708	0.726
BlockDist_tok.bioc_lc_sw.nltk_cf.blagec_ner.none	0.768	0.775	0.772	0.711	0.662	0.686	0.729	0.711	0.720	0.726
Qgram_tok.bioc_lc_sw.nltk_cf.none_ner.none	0.736	0.754	0.745	0.685	0.658	0.671	0.757	0.764	0.760	0.726
Qgram_tok.bioc_lc_sw.none_cf.default_ner.none	0.715	0.752	0.733	0.692	0.680	0.686	0.741	0.771	0.756	0.725
OverlapCoeff_tok.whitesp_lc_sw.biosses_cf.biosses_ner.none	0.771	0.794	0.782	0.687	0.557	0.615	0.773	0.782	0.778	0.725
OverlapCoeff_tok.whitesp_lc_sw.nltk_cf.default_ner.none	0.770	0.789	0.779	0.685	0.555	0.614	0.778	0.786	0.782	0.725
Qgram_tok.corenlp_notlc_sw.nltk_cf.default_ner.none	0.709	0.739	0.723	0.708	0.676	0.691	0.757	0.760	0.758	0.724
Qgram_tok.whitesp_notlc_sw.nltk_cf.default_ner.none	0.709	0.740	0.724	0.707	0.675	0.691	0.756	0.760	0.758	0.724
Qgram_tok.corenlp_notlc_sw.biosses_cf.none_ner.none	0.708	0.740	0.724	0.708	0.674	0.691	0.755	0.760	0.758	0.724
Qgram_tok.whitesp_notlc_sw.nltk_cf.biosses_ner.none	0.712	0.745	0.728	0.699	0.669	0.684	0.758	0.761	0.760	0.724
Qgram_tok.bioc_lc_sw.none_cf.blagec_ner.none	0.723	0.755	0.738	0.691	0.678	0.684	0.735	0.762	0.749	0.724
Qgram_tok.corenlp_notlc_sw.biosses_cf.default_ner.none	0.706	0.737	0.721	0.709	0.677	0.692	0.755	0.759	0.757	0.724
Qgram_tok.whitesp_notlc_sw.biosses_cf.default_ner.none	0.706	0.738	0.722	0.708	0.676	0.691	0.755	0.759	0.757	0.723
Qgram_tok.corenlp_notlc_sw.nltk_cf.biosses_ner.none	0.711	0.742	0.726	0.700	0.670	0.685	0.756	0.761	0.759	0.723
OverlapCoeff_tok.whitesp_lc_sw.biosses_cf.default_ner.none	0.765	0.785	0.775	0.686	0.556	0.614	0.776	0.783	0.780	0.723
OverlapCoeff_tok.bioc_lc_sw.nltk_cf.biosses_ner.none	0.776	0.796	0.786	0.673	0.546	0.603	0.774	0.785	0.780	0.723
Qgram_tok.whitesp_notlc_sw.biosses_cf.biosses_ner.none	0.710	0.743	0.726	0.700	0.669	0.684	0.757	0.759	0.758	0.723
Qgram_tok.whitesp_lc_sw.none_cf.none_ner.none	0.700	0.733	0.716	0.706	0.694	0.700	0.737	0.767	0.752	0.722
Qgram_tok.corenlp_notlc_sw.biosses_cf.blagec_ner.none	0.709	0.740	0.724	0.700	0.671	0.685	0.756	0.760	0.758	0.722
Qgram_tok.corenlp_notlc_sw.biosses_cf.biosses_ner.none	0.709	0.740	0.724	0.700	0.671	0.685	0.755	0.760	0.758	0.722
Qgram_tok.corenlp_lc_sw.none_cf.none_ner.none	0.704	0.733	0.718	0.704	0.691	0.698	0.734	0.767	0.750	0.722
OverlapCoeff_tok.bioc_lc_sw.nltk_cf.default_ner.none	0.770	0.788	0.779	0.674	0.547	0.604	0.778	0.786	0.782	0.721
Jaccard_tok.corenlp_lc_sw.nltk_cf.blagec_ner.none	0.753	0.778	0.766	0.711	0.682	0.696	0.690	0.715	0.702	0.721
OverlapCoeff_tok.bioc_lc_sw.biosses_cf.blagec_ner.none	0.771	0.793	0.782	0.673	0.547	0.603	0.773	0.783	0.778	0.721
OverlapCoeff_tok.bioc_lc_sw.biosses_cf.biosses_ner.none	0.771	0.793	0.782	0.673	0.547	0.603	0.773	0.782	0.778	0.721
Qgram_tok.whitesp_notlc_sw.biosses_cf.blagec_ner.none	0.702	0.736	0.719	0.700	0.669	0.684	0.757	0.760	0.759	0.721
Qgram_tok.whitesp_notlc_sw.nltk_cf.blagec_ner.none	0.705	0.737	0.721	0.699	0.669	0.683	0.750	0.760	0.755	0.720
OverlapCoeff_tok.bioc_lc_sw.biosses_cf.default_ner.none	0.765	0.784	0.775	0.674	0.547	0.604	0.776	0.783	0.780	0.719
Qgram_tok.bioc_notlc_sw.nltk_cf.default_ner.none	0.709	0.741	0.725	0.692	0.659	0.675	0.756	0.760	0.758	0.719
OverlapCoeff_tok.whitesp_lc_sw.biosses_cf.blagec_ner.none	0.752	0.774	0.763	0.687	0.559	0.617	0.773	0.783	0.778	0.719
Qgram_tok.corenlp_notlc_sw.nltk_cf.blagec_ner.none	0.706	0.735	0.720	0.699	0.670	0.684	0.748	0.758	0.753	0.719
Qgram_tok.corenlp_notlc_sw.nltk_cf.none_ner.none	0.703	0.731	0.717	0.699	0.669	0.684	0.750	0.762	0.756	0.719
Qgram_tok.bioc_notlc_sw.nltk_cf.biosses_ner.none	0.713	0.745	0.729	0.683	0.652	0.667	0.758	0.761	0.760	0.719
Qgram_tok.bioc_notlc_sw.biosses_cf.none_ner.none	0.710	0.739	0.724	0.691	0.657	0.674	0.755	0.760	0.758	0.719
Qgram_tok.bioc_notlc_sw.biosses_cf.default_ner.none	0.707	0.739	0.722	0.693	0.660	0.676	0.755	0.759	0.757	0.718
Qgram_tok.bioc_lc_sw.none_cf.none_ner.none	0.710	0.739	0.724	0.685	0.674	0.680	0.737	0.767	0.752	0.718
OverlapCoeff_tok.bioc_lc_sw.biosses_cf.none_ner.none	0.763	0.781	0.772	0.672	0.545	0.602	0.777	0.784	0.780	0.718
Qgram_tok.bioc_notlc_sw.biosses_cf.blagec_ner.none	0.711	0.743	0.727	0.684	0.653	0.668	0.757	0.760	0.759	0.718
BlockDist_tok.whitesp_notlc_sw.nltk_cf.default_ner.none	0.718	0.746	0.732	0.695	0.648	0.671	0.755	0.746	0.750	0.718
Qgram_tok.whitesp_notlc_sw.nltk_cf.none_ner.none	0.697	0.723	0.710	0.702	0.671	0.686	0.752	0.762	0.757	0.718
Jaccard_tok.bioc_lc_sw.nltk_cf.blagec_ner.none	0.761	0.782	0.772	0.688	0.658	0.673	0.705	0.712	0.708	0.718
Qgram_tok.bioc_notlc_sw.biosses_cf.biosses_ner.none	0.711	0.743	0.726	0.684	0.653	0.668	0.757	0.759	0.758	0.718
Qgram_tok.whitesp_notlc_sw.biosses_cf.none_ner.none	0.694	0.721	0.707	0.704	0.672	0.688	0.755	0.760	0.758	0.718
BlockDist_tok.whitesp_notlc_sw.nltk_cf.biosses_ner.none	0.718	0.746	0.732	0.695	0.647	0.670	0.755	0.745	0.750	0.717
BlockDist_tok.corenlp_notlc_sw.nltk_cf.default_ner.none	0.712	0.728	0.720	0.703	0.654	0.678	0.755	0.751	0.753	0.717
BlockDist_tok.corenlp_notlc_sw.nltk_cf.biosses_ner.none	0.712	0.728	0.720	0.702	0.653	0.677	0.754	0.751	0.752	0.716
BlockDist_tok.whitesp_notlc_sw.biosses_cf.default_ner.none	0.712	0.741	0.727	0.697	0.650	0.673	0.753	0.744	0.749	0.716
Qgram_tok.whitesp_notlc_sw.none_cf.biosses_ner.none	0.686	0.724	0.705	0.702	0.688	0.695	0.733	0.763	0.748	0.716
BlockDist_tok.whitesp_notlc_sw.biosses_cf.biosses_ner.none	0.712	0.741	0.726	0.697	0.649	0.672	0.754	0.743	0.748	0.716
BlockDist_tok.corenlp_notlc_sw.biosses_cf.default_ner.none	0.706	0.723	0.714	0.704	0.656	0.679	0.755	0.750	0.752	0.715
Qgram_tok.corenlp_notlc_sw.none_cf.biosses_ner.none	0.685	0.724	0.704	0.703	0.689	0.696	0.732	0.760	0.746	0.715
Jaccard_tok.corenlp_lc_sw.none_cf.default_ner.none	0.731	0.780	0.755	0.691	0.681	0.686	0.682	0.728	0.704	0.715
Qgram_tok.whitesp_notlc_sw.none_cf.default_ner.none	0.683	0.722	0.702	0.702	0.689	0.696	0.732	0.762	0.747	0.715
Qgram_tok.corenlp_notlc_sw.none_cf.default_ner.none	0.683	0.722	0.702	0.702	0.689	0.696	0.732	0.762	0.747	0.715
BlockDist_tok.corenlp_notlc_sw.biosses_cf.biosses_ner.none	0.706	0.723	0.714	0.704	0.655	0.678	0.753	0.750	0.752	0.715
Qgram_tok.bioc_notlc_sw.nltk_cf.blagec_ner.none	0.708	0.739	0.723	0.683	0.652	0.667	0.750	0.760	0.755	0.715
BlockDist_tok.corenlp_notlc_sw.biosses_cf.blagec_ner.none	0.706	0.723	0.715	0.704	0.655	0.678	0.754	0.749	0.752	0.715
BlockDist_tok.corenlp_notlc_sw.biosses_cf.none_ner.none	0.707	0.731	0.719	0.701	0.647	0.673	0.753	0.752	0.752	0.715
Jaccard_tok.corenlp_lc_sw.none_cf.biosses_ner.none	0.731	0.780	0.755	0.691	0.680	0.686	0.680	0.727	0.703	0.714
Qgram_tok.bioc_notlc_sw.nltk_cf.none_ner.none	0.705	0.731	0.718	0.684	0.653	0.668	0.752	0.762	0.757	0.714
Qgram_tok.corenlp_notlc_sw.none_cf.blagec_ner.none	0.682	0.724	0.702	0.702	0.688	0.695	0.726	0.754	0.740	0.712
BlockDist_tok.bioc_notlc_sw.nltk_cf.default_ner.none	0.719	0.744	0.731	0.679	0.632	0.655	0.755	0.746	0.750	0.712
Jaccard_tok.whitesp_lc_sw.biosses_cf.none_ner.none	0.686	0.727	0.706	0.664	0.645	0.654	0.756	0.797	0.776	0.712

Table A.3: Part 3 of 5. Pearson (r), Spearman (*ho*) and Harmonic score (h) obtained by the String-based similarity methods evaluated herein.

	BIOSSES			MedSTS			CTR			Avg
	r	ρ	h	r	ρ	h	r	ρ	h	Avg
BlockDist_tok.bioc_notlc_sw.nltk_cf.biosses_ner.none	0.719	0.745	0.732	0.678	0.631	0.654	0.755	0.745	0.750	0.712
Qgram_tok.whitesp_notlc_sw.none_cf.blagec_ner.none	0.681	0.716	0.698	0.702	0.687	0.695	0.727	0.756	0.741	0.711
BlockDist_tok.bioc_notlc_sw.biosses_cf.blagec_ner.none	0.713	0.741	0.727	0.679	0.632	0.655	0.755	0.743	0.749	0.710
BlockDist_tok.bioc_notlc_sw.biosses_cf.default_ner.none	0.713	0.739	0.725	0.680	0.634	0.656	0.753	0.744	0.749	0.710
BlockDist_tok.bioc_notlc_sw.biosses_cf.biosses_ner.none	0.713	0.741	0.727	0.679	0.632	0.655	0.754	0.743	0.748	0.710
Qgram_tok.bioc_notlc_sw.none_cf.biosses_ner.none	0.688	0.724	0.705	0.682	0.669	0.676	0.733	0.763	0.748	0.710
BlockDist_tok.whitesp_notlc_sw.biosses_cf.blagec_ner.none	0.691	0.724	0.707	0.697	0.650	0.673	0.755	0.743	0.749	0.710
BlockDist_tok.bioc_notlc_sw.biosses_cf.none_ner.none	0.713	0.739	0.725	0.678	0.632	0.654	0.753	0.745	0.749	0.709
Qgram_tok.bioc_notlc_sw.none_cf.default_ner.none	0.684	0.722	0.703	0.684	0.671	0.677	0.732	0.762	0.747	0.709
Qgram_tok.corenlp_notlc_sw.none_cf.none_ner.none	0.678	0.711	0.694	0.696	0.682	0.689	0.727	0.756	0.741	0.708
Qgram_tok.whitesp_notlc_sw.none_cf.none_ner.none	0.670	0.700	0.685	0.697	0.684	0.691	0.730	0.762	0.745	0.707
Jaccard_tok.whitesp_lc_sw.none_cf.biosses_ner.none	0.715	0.759	0.736	0.683	0.672	0.677	0.692	0.717	0.704	0.706
Qgram_tok.bioc_notlc_sw.none_cf.blagec_ner.none	0.684	0.720	0.701	0.682	0.668	0.675	0.727	0.756	0.741	0.706
BlockDist_tok.corenlp_lc_sw.none_cf.default_ner.none	0.704	0.751	0.727	0.679	0.675	0.677	0.713	0.712	0.713	0.705
Jaccard_tok.whitesp_lc_sw.none_cf.default_ner.none	0.710	0.755	0.732	0.683	0.672	0.677	0.693	0.720	0.706	0.705
BlockDist_tok.corenlp_lc_sw.none_cf.biosses_ner.none	0.704	0.750	0.727	0.679	0.674	0.677	0.712	0.710	0.711	0.705
Jaccard_tok.whitesp_notlc_sw.nltk_cf.default_ner.none	0.703	0.744	0.723	0.668	0.640	0.654	0.728	0.744	0.736	0.704
Jaccard_tok.whitesp_notlc_sw.nltk_cf.biosses_ner.none	0.703	0.742	0.722	0.668	0.640	0.654	0.729	0.744	0.737	0.704
Qgram_tok.bioc_notlc_sw.none_cf.none_ner.none	0.681	0.711	0.696	0.677	0.665	0.671	0.730	0.762	0.745	0.704
Jaccard_tok.whitesp_notlc_sw.biosses_cf.default_ner.none	0.698	0.738	0.717	0.670	0.642	0.656	0.727	0.743	0.735	0.703
Jaccard_tok.whitesp_notlc_sw.biosses_cf.biosses_ner.none	0.697	0.737	0.717	0.670	0.641	0.655	0.728	0.743	0.735	0.703
OverlapCoeff_tok.corenlp_lc_sw.nltk_cf.blagec_ner.none	0.765	0.774	0.770	0.693	0.560	0.619	0.723	0.714	0.718	0.702
Jaccard_tok.bioc_lc_sw.none_cf.biosses_ner.none	0.717	0.759	0.737	0.666	0.655	0.661	0.692	0.717	0.704	0.701
Jaccard_tok.corenlp_notlc_sw.nltk_cf.default_ner.none	0.689	0.727	0.708	0.676	0.649	0.662	0.714	0.749	0.731	0.700
Jaccard_tok.bioc_lc_sw.nltk_cf.none_ner.none	0.740	0.764	0.752	0.675	0.647	0.661	0.705	0.672	0.688	0.700
BlockDist_tok.bioc_lc_sw.nltk_cf.none_ner.none	0.746	0.755	0.750	0.686	0.645	0.665	0.712	0.660	0.685	0.700
Jaccard_tok.bioc_lc_sw.none_cf.default_ner.none	0.711	0.756	0.733	0.667	0.655	0.661	0.693	0.720	0.706	0.700
BlockDist_tok.corenlp_lc_sw.nltk_cf.none_ner.none	0.735	0.743	0.739	0.706	0.658	0.681	0.705	0.656	0.679	0.700
Jaccard_tok.corenlp_notlc_sw.nltk_cf.biosses_ner.none	0.689	0.727	0.707	0.676	0.648	0.662	0.712	0.749	0.730	0.700
Jaccard_tok.bioc_notlc_sw.nltk_cf.default_ner.none	0.704	0.741	0.722	0.655	0.626	0.640	0.728	0.744	0.736	0.699
Jaccard_tok.bioc_notlc_sw.nltk_cf.biosses_ner.none	0.704	0.740	0.722	0.654	0.624	0.639	0.729	0.744	0.737	0.699
Jaccard_tok.corenlp_notlc_sw.biosses_cf.default_ner.none	0.684	0.724	0.703	0.677	0.650	0.663	0.712	0.748	0.730	0.699
Jaccard_tok.corenlp_notlc_sw.biosses_cf.blagec_ner.none	0.684	0.724	0.703	0.677	0.649	0.663	0.712	0.748	0.730	0.699
Jaccard_tok.corenlp_notlc_sw.biosses_cf.biosses_ner.none	0.683	0.724	0.703	0.677	0.649	0.663	0.711	0.748	0.729	0.698
Jaccard_tok.corenlp_notlc_sw.biosses_cf.none_ner.none	0.683	0.730	0.705	0.674	0.644	0.659	0.712	0.750	0.731	0.698
Jaccard_tok.bioc_notlc_sw.biosses_cf.blagec_ner.none	0.699	0.736	0.717	0.655	0.626	0.640	0.729	0.743	0.736	0.698
Jaccard_tok.corenlp_lc_sw.nltk_cf.none_ner.none	0.730	0.754	0.742	0.695	0.664	0.679	0.682	0.662	0.672	0.698
OverlapCoeff_tok.whitesp_lc_sw.nltk_cf.blagec_ner.none	0.758	0.777	0.767	0.686	0.558	0.616	0.714	0.707	0.710	0.698
Jaccard_tok.bioc_notlc_sw.biosses_cf.biosses_ner.none	0.699	0.737	0.717	0.655	0.626	0.640	0.728	0.743	0.735	0.698
Jaccard_tok.bioc_notlc_sw.biosses_cf.default_ner.none	0.699	0.736	0.717	0.656	0.627	0.641	0.727	0.743	0.735	0.698
Jaccard_tok.bioc_notlc_sw.biosses_cf.none_ner.none	0.699	0.734	0.716	0.654	0.625	0.640	0.727	0.745	0.736	0.697
Jaccard_tok.corenlp_lc_sw.none_cf.blagec_ner.none	0.718	0.764	0.740	0.688	0.674	0.681	0.648	0.692	0.669	0.697
Jaccard_tok.whitesp_notlc_sw.biosses_cf.blagec_ner.none	0.675	0.717	0.695	0.670	0.643	0.656	0.729	0.743	0.736	0.696
BlockDist_tok.whitesp_lc_sw.none_cf.biosses_ner.none	0.694	0.744	0.718	0.668	0.664	0.666	0.700	0.701	0.701	0.695
BlockDist_tok.whitesp_lc_sw.nltk_cf.none_ner.none	0.714	0.752	0.733	0.685	0.644	0.664	0.712	0.660	0.685	0.694
OverlapCoeff_tok.whitesp_lc_sw.biosses_cf.none_ner.none	0.680	0.721	0.700	0.655	0.553	0.600	0.777	0.784	0.780	0.693
BlockDist_tok.whitesp_lc_sw.none_cf.default_ner.none	0.686	0.736	0.710	0.669	0.664	0.666	0.701	0.704	0.703	0.693
OverlapCoeff_tok.bioc_lc_sw.nltk_cf.blagec_ner.none	0.757	0.771	0.763	0.671	0.542	0.599	0.714	0.707	0.710	0.691
BlockDist_tok.bioc_lc_sw.none_cf.biosses_ner.none	0.694	0.741	0.717	0.648	0.647	0.647	0.700	0.701	0.701	0.688
Jaccard_tok.whitesp_lc_sw.none_cf.blagec_ner.none	0.693	0.738	0.715	0.683	0.673	0.678	0.657	0.687	0.671	0.688
BlockDist_tok.whitesp_notlc_sw.nltk_cf.blagec_ner.none	0.697	0.730	0.713	0.695	0.648	0.671	0.686	0.668	0.677	0.687
BlockDist_tok.bioc_lc_sw.none_cf.default_ner.none	0.687	0.734	0.710	0.648	0.648	0.648	0.701	0.704	0.703	0.687
BlockDist_tok.corenlp_lc_sw.none_cf.blagec_ner.none	0.689	0.726	0.707	0.677	0.670	0.673	0.677	0.681	0.679	0.687
OverlapCoeff_tok.corenlp_notlc_sw.nltk_cf.default_ner.none	0.710	0.726	0.718	0.657	0.532	0.588	0.753	0.745	0.749	0.685
Jaccard_tok.corenlp_lc_sw.none_cf.none_ner.none	0.709	0.741	0.724	0.673	0.659	0.666	0.646	0.682	0.663	0.684
OverlapCoeff_tok.corenlp_notlc_sw.nltk_cf.biosses_ner.none	0.710	0.725	0.717	0.656	0.532	0.588	0.752	0.744	0.748	0.684
OverlapCoeff_tok.corenlp_lc_sw.none_cf.default_ner.none	0.727	0.774	0.750	0.660	0.556	0.604	0.698	0.702	0.700	0.684
Jaccard_tok.whitesp_lc_sw.nltk_cf.none_ner.none	0.693	0.734	0.713	0.660	0.642	0.651	0.705	0.672	0.688	0.684
OverlapCoeff_tok.corenlp_lc_sw.none_cf.biosses_ner.none	0.726	0.774	0.750	0.660	0.556	0.604	0.697	0.701	0.699	0.684
OverlapCoeff_tok.corenlp_notlc_sw.biosses_cf.none_ner.none	0.708	0.725	0.716	0.657	0.529	0.586	0.752	0.745	0.748	0.684
Jaccard_tok.bioc_lc_sw.none_cf.blagec_ner.none	0.704	0.742	0.723	0.663	0.649	0.656	0.657	0.687	0.671	0.683
OverlapCoeff_tok.corenlp_notlc_sw.biosses_cf.blagec_ner.none	0.705	0.721	0.713	0.658	0.534	0.589	0.751	0.743	0.747	0.683
OverlapCoeff_tok.corenlp_notlc_sw.biosses_cf.default_ner.none	0.705	0.720	0.713	0.658	0.533	0.589	0.751	0.743	0.747	0.683
OverlapCoeff_tok.corenlp_notlc_sw.biosses_cf.biosses_ner.none	0.705	0.720	0.712	0.658	0.534	0.589	0.751	0.742	0.747	0.683
OverlapCoeff_tok.whitesp_notlc_sw.nltk_cf.default_ner.none	0.708	0.737	0.723	0.650	0.525	0.581	0.748	0.739	0.744	0.683
BlockDist_tok.whitesp_notlc_sw.biosses_cf.none_ner.none	0.639	0.685	0.661	0.657	0.617	0.636	0.753	0.745	0.749	0.682
OverlapCoeff_tok.whitesp_notlc_sw.nltk_cf.biosses_ner.none	0.705	0.734	0.719	0.650	0.525	0.581	0.747	0.739	0.743	0.681
OverlapCoeff_tok.whitesp_notlc_sw.biosses_cf.default_ner.none	0.703	0.729	0.716	0.651	0.527	0.583	0.747	0.738	0.742	0.680
BlockDist_tok.corenlp_notlc_sw.nltk_cf.blagec_ner.none	0.680	0.688	0.684	0.700	0.649	0.673	0.692	0.670	0.681	0.679
OverlapCoeff_tok.whitesp_notlc_sw.biosses_cf.biosses_ner.none	0.699	0.726	0.712	0.651	0.527	0.583	0.746	0.737	0.741	0.679
OverlapCoeff_tok.bioc_notlc_sw.nltk_cf.default_ner.none	0.709	0.735	0.722	0.637	0.515	0.570	0.748	0.739	0.744	0.678
OverlapCoeff_tok.bioc_notlc_sw.nltk_cf.biosses_ner.none	0.704	0.731	0.717	0.636	0.515	0.569	0.747	0.739	0.743	0.676
BlockDist_tok.whitesp_lc_sw.none_cf.blagec_ner.none	0.672	0.720	0.696	0.669	0.665	0.667	0.665	0.668	0.666	0.676
Jaccard_tok.whitesp_notlc_sw.nltk_cf.blagec_ner.none	0.680	0.723	0.701	0.668	0.641	0.654	0.675	0.671	0.673	0.676
OverlapCoeff_tok.bioc_notlc_sw.biosses_cf.default_ner.none	0.703	0.727	0.715	0.638	0.517	0.571	0.747	0.738	0.742	0.676
OverlapCoeff_tok.bioc_notlc_sw.biosses_cf.none_ner.none	0.702	0.726	0.713	0.637	0.515	0.570	0.748	0.739	0.744	0.676
OverlapCoeff_tok.bioc_notlc_sw.biosses_cf.blagec_ner.none	0.699	0.723	0.711	0.638	0.517	0.571	0.746	0.738	0.742	0.675
OverlapCoeff_tok.bioc_notlc_sw.biosses_cf.biosses_ner.none	0.699	0.723	0.711	0.638	0.517	0.571	0.746	0.737	0.741	0.674
Jaccard_tok.bioc_lc_sw.none_cf.none_ner.none	0.696	0.711	0.704	0.652	0.638	0.645	0.662	0.684	0.673	0.674
OverlapCoeff_tok.whitesp_notlc_sw.biosses_cf.blagec_ner.none	0.677	0.708	0.692	0.653	0.531	0.585	0.746	0.738	0.742	0.673
OverlapCoeff_tok.corenlp_lc_sw.nltk_cf.none_ner.none	0.744	0.747	0.746	0.673	0.547	0.603	0.698	0.642	0.669	0.673
BlockDist_tok.corenlp_notlc_sw.none_cf.default_ner.none	0.649	0.691	0.669	0.656	0.649	0.652	0.699	0.692	0.696	0.673
Jaccard_tok.corenlp_notlc_sw.none_cf.default_ner.none	0.662	0.705	0.683	0.659	0.650	0.654	0.667	0.693	0.680	0.672
BlockDist_tok.corenlp_notlc_sw.none_cf.biosses_ner.none	0.649	0.691	0.670	0.656	0.648	0.652	0.698	0.691	0.695	0.672
BlockDist_tok.bioc_notlc_sw.nltk_cf.blagec_ner.none	0.682	0.696	0.689	0.676	0.626	0.650	0.686	0.668	0.677	0.672
Jaccard_tok.corenlp_notlc_sw.none_cf.biosses_ner.none	0.662	0.704	0.683	0.659	0.650	0.654	0.666	0.691	0.678	0.672
BlockDist_tok.corenlp_lc_sw.none_cf.none_ner.none	0.687	0.707	0.697	0.656	0.650	0.653	0.663	0.661	0.662	0.671
OverlapCoeff_tok.whitesp_lc_sw.none_cf.biosses_ner.none	0.698	0.754	0.725	0.651	0.549	0.595	0.685	0.689	0.687	0.669
BlockDist_tok.bioc_lc_sw.none_cf.blagec_ner.none	0.678	0.716	0.696	0.646	0.643	0.645	0.665	0.668	0.666	0.669
OverlapCoeff_tok.whitesp_lc_sw.none_cf.default_ner.none	0.695	0.754	0.723	0.651	0.549	0.595	0.687	0.690	0.688	0.669
OverlapCoeff_tok.corenlp_lc_sw.none_cf.blagec_ner.none	0.715	0.759	0.737	0.658	0.552	0.601	0.662	0.676	0.669	0.669
Jaccard_tok.corenlp_notlc_sw.nltk_cf.blagec_ner.none	0.666	0.693	0.679	0.673	0.642	0.657	0.664	0.674	0.669	0.668
Jaccard_tok.whitesp_notlc_sw.biosses_cf.none_ner.none	0.621	0.668	0.643	0.629	0.611	0.620	0.727	0.745	0.736	0.666
Jaccard_tok.whitesp_notlc_sw.none_cf.default_ner.none	0.651	0.693	0.671	0.652	0.642	0.647	0.674	0.687	0.680	0.666
Jaccard_tok.whitesp_notlc_sw.none_cf.biosses_ner.none	0.652	0.693	0.672	0.652	0.642	0.647	0.673	0.685	0.679	0.666
Jaccard_tok.bioc_notlc_sw.nltk_cf.blagec_ner.none	0.678	0.701	0.689	0.651	0.619	0.634	0.675	0.671	0.673	0.666
OverlapCoeff_tok.bioc_lc_sw.nltk_cf.none_ner.none	0.737	0.746	0.742	0.656	0.533	0.588	0.697	0.639	0.667	0.665
OverlapCoeff_tok.bioc_lc_sw.none_cf.biosses_ner.none	0.699	0.753	0.725	0.636	0.537	0.582	0.685	0.689	0.687	0.665
OverlapCoeff_tok.bioc_lc_sw.none_cf.default_ner.none	0.696	0.752	0.723	0.637	0.538	0.583	0.687	0.690	0.688	0.665
BlockDist_tok.whitesp_notlc_sw.none_cf.default_ner.none	0.637	0.686	0.661	0.649	0.641	0.645	0.688	0.687	0.688	0.664
BlockDist_tok.whitesp_notlc_sw.none_cf.biosses_ner.none	0.638	0.688	0.662	0.648	0.640	0.644	0.688	0.684	0.686	0.664
BlockDist_tok.bioc_lc_sw.none_cf.none_ner.none	0.681	0.703	0.692	0.631	0.631	0.631	0.661	0.659	0.660	0.661
Jaccard_tok.bioc_notlc_sw.none_cf.default_ner.none	0.652	0.692	0.671	0.636	0.626	0.631	0.674	0.687	0.680	0.661
Jaccard_tok.bioc_notlc_sw.none_cf.biosses_ner.none	0.653	0.693	0.672	0.636	0.625	0.630	0.673	0.685	0.679	0.661
BlockDist_tok.bioc_notlc_sw.none_cf.default_ner.none	0.637	0.685	0.660	0.629	0.624	0.626	0.688	0.687	0.688	0.658

Table A.4: Part 4 of 5. Pearson (r), Spearman (*ho*) and Harmonic score (h) obtained by the String-based similarity methods evaluated herein.

	BIOSSES			MedSTS			CTR			Avg
	r	ρ	h	r	ρ	h	r	ρ	h	Avg
BlockDist_tok.bioc_notlc_sw.none_cf.biosses_ner.none	0.638	0.687	0.662	0.629	0.623	0.626	0.688	0.684	0.686	0.658
BlockDist_tok.bioc_notlc_sw.nltk_cf.none_ner.none	0.676	0.698	0.687	0.656	0.613	0.634	0.674	0.631	0.652	0.658
OverlapCoeff_tok.whitesp_lc_sw.nltk_cf.none_ner.none	0.688	0.726	0.707	0.651	0.551	0.597	0.697	0.639	0.667	0.657
Jaccard_tok.bioc_notlc_sw.nltk_cf.none_ner.none	0.673	0.696	0.684	0.641	0.611	0.625	0.675	0.644	0.659	0.656
OverlapCoeff_tok.corenlp_notlc_sw.nltk_cf.blagec_ner.none	0.691	0.705	0.698	0.655	0.528	0.585	0.694	0.677	0.685	0.656
Jaccard_tok.corenlp_notlc_sw.none_cf.blagec_ner.none	0.647	0.687	0.666	0.656	0.645	0.651	0.631	0.661	0.646	0.654
OverlapCoeff_tok.whitesp_notlc_sw.nltk_cf.blagec_ner.none	0.683	0.716	0.699	0.651	0.528	0.583	0.687	0.674	0.680	0.654
BlockDist_tok.corenlp_notlc_sw.nltk_cf.none_ner.none	0.658	0.674	0.666	0.675	0.627	0.650	0.670	0.623	0.646	0.654
OverlapCoeff_tok.whitesp_lc_sw.none_cf.blagec_ner.none	0.677	0.733	0.704	0.652	0.550	0.597	0.651	0.666	0.658	0.653
OverlapCoeff_tok.whitesp_notlc_sw.biosses_cf.none_ner.none	0.623	0.672	0.647	0.619	0.523	0.567	0.748	0.739	0.744	0.653
BlockDist_tok.corenlp_notlc_sw.none_cf.blagec_ner.none	0.630	0.664	0.647	0.655	0.645	0.650	0.661	0.659	0.660	0.652
Jaccard_tok.corenlp_notlc_sw.nltk_cf.none_ner.none	0.649	0.682	0.665	0.660	0.628	0.644	0.657	0.637	0.647	0.652
OverlapCoeff_tok.corenlp_lc_sw.none_cf.none_ner.none	0.701	0.729	0.715	0.639	0.538	0.584	0.650	0.661	0.656	0.652
Jaccard_tok.whitesp_lc_sw.none_cf.none_ner.none	0.620	0.646	0.633	0.649	0.647	0.648	0.662	0.684	0.673	0.651
BlockDist_tok.whitesp_notlc_sw.nltk_cf.none_ner.none	0.649	0.693	0.670	0.651	0.612	0.631	0.674	0.631	0.652	0.651
OverlapCoeff_tok.bioc_lc_sw.none_cf.blagec_ner.none	0.687	0.740	0.713	0.635	0.533	0.580	0.651	0.666	0.658	0.650
OverlapCoeff_tok.corenlp_notlc_sw.none_cf.default_ner.none	0.665	0.711	0.687	0.626	0.530	0.574	0.684	0.683	0.683	0.648
Jaccard_tok.whitesp_notlc_sw.none_cf.blagec_ner.none	0.629	0.674	0.651	0.652	0.643	0.648	0.636	0.656	0.646	0.648
OverlapCoeff_tok.corenlp_notlc_sw.none_cf.biosses_ner.none	0.664	0.711	0.687	0.626	0.530	0.574	0.683	0.682	0.683	0.648
Jaccard_tok.corenlp_notlc_sw.none_cf.none_ner.none	0.642	0.670	0.656	0.645	0.632	0.638	0.631	0.657	0.644	0.646
OverlapCoeff_tok.bioc_notlc_sw.nltk_cf.blagec_ner.none	0.681	0.700	0.690	0.635	0.510	0.566	0.687	0.674	0.680	0.645
BlockDist_tok.whitesp_notlc_sw.none_cf.blagec_ner.none	0.617	0.666	0.640	0.649	0.641	0.645	0.648	0.648	0.648	0.644
Jaccard_tok.bioc_notlc_sw.none_cf.blagec_ner.none	0.638	0.673	0.655	0.634	0.621	0.627	0.636	0.656	0.646	0.643
Jaccard_tok.whitesp_notlc_sw.nltk_cf.none_ner.none	0.629	0.677	0.652	0.625	0.608	0.617	0.675	0.644	0.659	0.643
Jaccard_tok.bioc_notlc_sw.none_cf.none_ner.none	0.640	0.663	0.652	0.625	0.612	0.618	0.643	0.662	0.652	0.641
BlockDist_tok.corenlp_notlc_sw.none_cf.none_ner.none	0.631	0.649	0.640	0.640	0.628	0.634	0.648	0.646	0.647	0.640
BlockDist_tok.bioc_notlc_sw.none_cf.blagec_ner.none	0.619	0.658	0.638	0.628	0.620	0.624	0.648	0.648	0.648	0.637
BlockDist_tok.whitesp_lc_sw.none_cf.none_ner.none	0.593	0.631	0.612	0.635	0.640	0.637	0.661	0.659	0.660	0.636
OverlapCoeff_tok.whitesp_notlc_sw.none_cf.default_ner.none	0.641	0.695	0.667	0.620	0.524	0.568	0.673	0.675	0.674	0.636
OverlapCoeff_tok.bioc_lc_sw.none_cf.none_ner.none	0.677	0.706	0.691	0.623	0.524	0.569	0.644	0.650	0.647	0.636
OverlapCoeff_tok.whitesp_notlc_sw.none_cf.biosses_ner.none	0.638	0.695	0.665	0.620	0.523	0.567	0.672	0.674	0.673	0.635
BlockDist_tok.bioc_notlc_sw.none_cf.none_ner.none	0.633	0.656	0.644	0.617	0.611	0.614	0.646	0.643	0.644	0.634
OverlapCoeff_tok.bioc_notlc_sw.none_cf.default_ner.none	0.642	0.696	0.668	0.605	0.512	0.555	0.673	0.675	0.674	0.632
OverlapCoeff_tok.corenlp_notlc_sw.nltk_cf.none_ner.none	0.674	0.681	0.677	0.639	0.518	0.572	0.676	0.617	0.645	0.631
OverlapCoeff_tok.bioc_notlc_sw.none_cf.biosses_ner.none	0.639	0.696	0.666	0.605	0.512	0.554	0.672	0.674	0.673	0.631
OverlapCoeff_tok.corenlp_notlc_sw.none_cf.blagec_ner.none	0.651	0.690	0.670	0.626	0.527	0.572	0.646	0.654	0.650	0.631
OverlapCoeff_tok.bioc_notlc_sw.nltk_cf.none_ner.none	0.680	0.697	0.688	0.623	0.504	0.557	0.673	0.620	0.645	0.630
OverlapCoeff_tok.whitesp_notlc_sw.nltk_cf.none_ner.none	0.633	0.680	0.655	0.616	0.521	0.565	0.673	0.620	0.645	0.622
OverlapCoeff_tok.whitesp_lc_sw.none_cf.none_ner.none	0.605	0.647	0.625	0.627	0.550	0.586	0.644	0.650	0.647	0.619
OverlapCoeff_tok.whitesp_notlc_sw.none_cf.blagec_ner.none	0.616	0.672	0.643	0.621	0.526	0.569	0.635	0.646	0.640	0.618
Jaccard_tok.whitesp_notlc_sw.none_cf.none_ner.none	0.565	0.597	0.581	0.620	0.619	0.620	0.643	0.662	0.652	0.618
OverlapCoeff_tok.corenlp_notlc_sw.none_cf.none_ner.none	0.642	0.663	0.653	0.611	0.516	0.559	0.637	0.641	0.639	0.617
OverlapCoeff_tok.bioc_notlc_sw.none_cf.blagec_ner.none	0.624	0.677	0.650	0.605	0.508	0.552	0.635	0.646	0.640	0.614
BlockDist_tok.whitesp_notlc_sw.none_cf.none_ner.none	0.548	0.592	0.569	0.618	0.617	0.617	0.646	0.643	0.644	0.610
OverlapCoeff_tok.bioc_notlc_sw.none_cf.none_ner.none	0.627	0.655	0.641	0.596	0.502	0.545	0.632	0.640	0.636	0.607
OverlapCoeff_tok.whitesp_notlc_sw.none_cf.none_ner.none	0.557	0.607	0.581	0.597	0.525	0.559	0.632	0.640	0.636	0.592
Levenshtein_tok.whitesp_notlc_sw.biosses_cf.none_ner.none	0.529	0.536	0.533	0.610	0.634	0.622	0.498	0.536	0.516	0.557
Levenshtein_tok.whitesp_lc_sw.biosses_cf.biosses_ner.none	0.547	0.529	0.538	0.623	0.644	0.633	0.480	0.520	0.499	0.557
Levenshtein_tok.whitesp_notlc_sw.biosses_cf.blagec_ner.none	0.523	0.518	0.521	0.613	0.636	0.625	0.500	0.543	0.521	0.555
Levenshtein_tok.whitesp_lc_sw.biosses_cf.blagec_ner.none	0.543	0.527	0.535	0.622	0.642	0.632	0.481	0.518	0.499	0.555
Levenshtein_tok.whitesp_notlc_sw.biosses_cf.biosses_ner.none	0.525	0.515	0.520	0.614	0.638	0.626	0.499	0.542	0.520	0.555
Levenshtein_tok.whitesp_lc_sw.biosses_cf.none_ner.none	0.544	0.536	0.540	0.618	0.640	0.629	0.480	0.513	0.496	0.555
Levenshtein_tok.whitesp_lc_sw.nltk_cf.biosses_ner.none	0.547	0.524	0.535	0.621	0.643	0.632	0.477	0.516	0.496	0.554

Table A.5: Part 5 of 5. Pearson (r), Spearman (*ho*) and Harmonic score (h) obtained by the String-based similarity methods evaluated herein.

	BIOSSES			MedSTS			CTR			Avg
	r	ρ	h	r	ρ	h	r	ρ	h	Avg
Levenshtein_tok.bioc_notlc_sw.biosses_cf.none_ner.none	0.534	0.529	0.532	0.602	0.624	0.613	0.498	0.536	0.516	0.554
Levenshtein_tok.bioc_lc_sw.biosses_cf.none_ner.none	0.551	0.537	0.544	0.610	0.630	0.620	0.480	0.513	0.496	0.553
Levenshtein_tok.whitesp_notlc_sw.nltk_cf.biosses_ner.none	0.527	0.512	0.519	0.613	0.636	0.625	0.495	0.535	0.514	0.553
Levenshtein_tok.bioc_lc_sw.biosses_cf.biosses_ner.none	0.549	0.529	0.539	0.610	0.630	0.620	0.480	0.520	0.499	0.553
Levenshtein_tok.bioc_lc_sw.biosses_cf.blagec_ner.none	0.548	0.529	0.539	0.610	0.630	0.620	0.481	0.518	0.499	0.552
Levenshtein_tok.whitesp_lc_sw.biosses_cf.default_ner.none	0.549	0.534	0.541	0.622	0.643	0.632	0.472	0.493	0.482	0.552
Levenshtein_tok.corenlp_notlc_sw.biosses_cf.blagec_ner.none	0.525	0.521	0.523	0.614	0.638	0.626	0.492	0.524	0.507	0.552
Levenshtein_tok.corenlp_notlc_sw.biosses_cf.biosses_ner.none	0.525	0.522	0.523	0.614	0.638	0.626	0.491	0.523	0.506	0.552
Levenshtein_tok.bioc_notlc_sw.biosses_cf.blagec_ner.none	0.527	0.515	0.521	0.602	0.625	0.613	0.500	0.543	0.521	0.551
Levenshtein_tok.whitesp_lc_sw.nltk_cf.default_ner.none	0.551	0.533	0.542	0.620	0.643	0.631	0.471	0.492	0.481	0.551
Levenshtein_tok.bioc_notlc_sw.biosses_cf.biosses_ner.none	0.527	0.516	0.521	0.602	0.625	0.613	0.499	0.542	0.520	0.551
Levenshtein_tok.corenlp_lc_sw.nltk_cf.biosses_ner.none	0.546	0.522	0.533	0.622	0.643	0.632	0.473	0.503	0.487	0.551
Levenshtein_tok.corenlp_notlc_sw.biosses_cf.none_ner.none	0.522	0.522	0.522	0.613	0.636	0.624	0.492	0.521	0.506	0.551
Levenshtein_tok.whitesp_notlc_sw.biosses_cf.default_ner.none	0.525	0.528	0.526	0.614	0.637	0.625	0.487	0.514	0.500	0.551
Levenshtein_tok.corenlp_lc_sw.biosses_cf.biosses_ner.none	0.544	0.519	0.531	0.622	0.644	0.633	0.473	0.503	0.488	0.551
Levenshtein_tok.corenlp_lc_sw.biosses_cf.none_ner.none	0.545	0.530	0.538	0.622	0.642	0.632	0.470	0.494	0.482	0.550
Levenshtein_tok.bioc_lc_sw.nltk_cf.biosses_ner.none	0.549	0.523	0.536	0.609	0.629	0.619	0.477	0.516	0.496	0.550
Levenshtein_tok.corenlp_lc_sw.biosses_cf.blagec_ner.none	0.543	0.519	0.531	0.622	0.644	0.633	0.473	0.500	0.486	0.550
Levenshtein_tok.corenlp_lc_sw.biosses_cf.default_ner.none	0.544	0.521	0.532	0.623	0.645	0.634	0.472	0.496	0.483	0.550
Levenshtein_tok.corenlp_notlc_sw.biosses_cf.default_ner.none	0.523	0.521	0.522	0.615	0.638	0.626	0.487	0.515	0.501	0.550
Levenshtein_tok.corenlp_lc_sw.nltk_cf.default_ner.none	0.545	0.522	0.534	0.622	0.644	0.633	0.471	0.494	0.482	0.550
Levenshtein_tok.bioc_notlc_sw.nltk_cf.biosses_ner.none	0.529	0.514	0.522	0.601	0.623	0.612	0.495	0.535	0.514	0.549
Levenshtein_tok.corenlp_notlc_sw.nltk_cf.biosses_ner.none	0.527	0.515	0.521	0.613	0.637	0.625	0.487	0.518	0.502	0.549
Levenshtein_tok.whitesp_notlc_sw.nltk_cf.none_ner.none	0.530	0.533	0.531	0.607	0.632	0.619	0.485	0.508	0.496	0.549
Levenshtein_tok.bioc_lc_sw.biosses_cf.default_ner.none	0.553	0.535	0.544	0.609	0.630	0.619	0.472	0.493	0.482	0.548
Levenshtein_tok.bioc_lc_sw.nltk_cf.default_ner.none	0.555	0.536	0.545	0.608	0.629	0.618	0.471	0.492	0.481	0.548
Levenshtein_tok.whitesp_notlc_sw.nltk_cf.default_ner.none	0.527	0.522	0.525	0.612	0.636	0.624	0.483	0.507	0.494	0.548
Levenshtein_tok.bioc_notlc_sw.biosses_cf.default_ner.none	0.528	0.530	0.529	0.602	0.624	0.613	0.487	0.514	0.500	0.548
Levenshtein_tok.corenlp_notlc_sw.nltk_cf.default_ner.none	0.525	0.513	0.519	0.614	0.638	0.626	0.483	0.506	0.494	0.546
Levenshtein_tok.whitesp_lc_sw.nltk_cf.blagec_ner.none	0.543	0.524	0.533	0.620	0.642	0.631	0.457	0.493	0.474	0.546
Levenshtein_tok.whitesp_lc_sw.nltk_cf.none_ner.none	0.541	0.525	0.533	0.616	0.638	0.627	0.466	0.484	0.475	0.545
Levenshtein_tok.whitesp_notlc_sw.nltk_cf.blagec_ner.none	0.526	0.517	0.521	0.612	0.635	0.623	0.475	0.506	0.490	0.545
Levenshtein_tok.corenlp_notlc_sw.nltk_cf.none_ner.none	0.521	0.532	0.526	0.602	0.628	0.615	0.482	0.505	0.493	0.545
Levenshtein_tok.bioc_notlc_sw.nltk_cf.default_ner.none	0.530	0.523	0.527	0.601	0.623	0.612	0.483	0.507	0.494	0.544
Levenshtein_tok.corenlp_lc_sw.nltk_cf.none_ner.none	0.541	0.516	0.528	0.611	0.635	0.623	0.466	0.497	0.481	0.544
Levenshtein_tok.corenlp_notlc_sw.nltk_cf.blagec_ner.none	0.525	0.517	0.521	0.611	0.635	0.623	0.471	0.500	0.485	0.543
Levenshtein_tok.corenlp_lc_sw.nltk_cf.blagec_ner.none	0.535	0.511	0.522	0.620	0.642	0.631	0.457	0.495	0.475	0.543
Levenshtein_tok.bioc_notlc_sw.nltk_cf.blagec_ner.none	0.518	0.507	0.512	0.599	0.622	0.610	0.475	0.506	0.490	0.538
Levenshtein_tok.bioc_lc_sw.nltk_cf.blagec_ner.none	0.531	0.511	0.521	0.608	0.628	0.618	0.457	0.493	0.474	0.538
Levenshtein_tok.whitesp_lc_sw.none_cf.biosses_ner.none	0.521	0.533	0.527	0.589	0.627	0.608	0.463	0.487	0.474	0.536
Levenshtein_tok.bioc_notlc_sw.nltk_cf.none_ner.none	0.510	0.499	0.505	0.592	0.617	0.604	0.485	0.508	0.496	0.535
Levenshtein_tok.bioc_lc_sw.nltk_cf.none_ner.none	0.523	0.504	0.513	0.600	0.623	0.611	0.466	0.484	0.475	0.533
Levenshtein_tok.bioc_lc_sw.none_cf.biosses_ner.none	0.525	0.537	0.531	0.575	0.612	0.593	0.463	0.487	0.474	0.533
Levenshtein_tok.whitesp_notlc_sw.none_cf.biosses_ner.none	0.501	0.517	0.509	0.586	0.623	0.604	0.468	0.495	0.481	0.531
Levenshtein_tok.whitesp_lc_sw.none_cf.blagec_ner.none	0.521	0.534	0.527	0.589	0.627	0.607	0.449	0.461	0.455	0.530
Levenshtein_tok.corenlp_lc_sw.none_cf.biosses_ner.none	0.510	0.519	0.515	0.589	0.628	0.608	0.457	0.471	0.464	0.529
Levenshtein_tok.whitesp_lc_sw.none_cf.default_ner.none	0.510	0.520	0.515	0.589	0.628	0.608	0.455	0.470	0.462	0.528
Levenshtein_tok.corenlp_lc_sw.none_cf.default_ner.none	0.509	0.518	0.514	0.589	0.628	0.608	0.455	0.470	0.462	0.528
Levenshtein_tok.bioc_notlc_sw.none_cf.biosses_ner.none	0.504	0.520	0.512	0.571	0.608	0.589	0.468	0.495	0.481	0.527
Levenshtein_tok.whitesp_notlc_sw.none_cf.blagec_ner.none	0.501	0.518	0.509	0.585	0.623	0.604	0.456	0.475	0.465	0.526
Levenshtein_tok.bioc_lc_sw.none_cf.default_ner.none	0.516	0.524	0.520	0.575	0.613	0.593	0.455	0.470	0.462	0.525
Levenshtein_tok.corenlp_notlc_sw.none_cf.biosses_ner.none	0.488	0.499	0.493	0.586	0.624	0.604	0.464	0.485	0.474	0.524
Levenshtein_tok.corenlp_lc_sw.none_cf.blagec_ner.none	0.507	0.516	0.511	0.588	0.626	0.607	0.446	0.456	0.451	0.523
Levenshtein_tok.bioc_lc_sw.none_cf.blagec_ner.none	0.516	0.522	0.519	0.574	0.612	0.592	0.449	0.461	0.455	0.522
Levenshtein_tok.corenlp_notlc_sw.none_cf.default_ner.none	0.486	0.497	0.491	0.586	0.624	0.604	0.462	0.479	0.470	0.522
Levenshtein_tok.whitesp_notlc_sw.none_cf.default_ner.none	0.487	0.494	0.490	0.586	0.624	0.604	0.462	0.479	0.470	0.522
Levenshtein_tok.whitesp_lc_sw.none_cf.none_ner.none	0.510	0.527	0.519	0.585	0.624	0.604	0.439	0.434	0.436	0.520
Levenshtein_tok.whitesp_notlc_sw.none_cf.none_ner.none	0.492	0.514	0.503	0.582	0.620	0.600	0.449	0.460	0.454	0.519
Levenshtein_tok.corenlp_notlc_sw.none_cf.blagec_ner.none	0.486	0.490	0.488	0.585	0.622	0.603	0.455	0.479	0.466	0.519
Levenshtein_tok.bioc_notlc_sw.none_cf.default_ner.none	0.493	0.501	0.497	0.571	0.609	0.589	0.462	0.479	0.470	0.519
Levenshtein_tok.bioc_notlc_sw.none_cf.blagec_ner.none	0.496	0.511	0.503	0.570	0.607	0.588	0.456	0.475	0.465	0.519
Levenshtein_tok.corenlp_lc_sw.none_cf.none_ner.none	0.505	0.523	0.513	0.582	0.621	0.601	0.441	0.442	0.441	0.519
Levenshtein_tok.corenlp_notlc_sw.none_cf.none_ner.none	0.484	0.509	0.496	0.579	0.617	0.598	0.452	0.461	0.457	0.517
Levenshtein_tok.bioc_lc_sw.none_cf.none_ner.none	0.511	0.526	0.518	0.568	0.607	0.587	0.439	0.434	0.436	0.514
Levenshtein_tok.bioc_notlc_sw.none_cf.none_ner.none	0.493	0.502	0.498	0.565	0.603	0.583	0.449	0.460	0.454	0.512

Table A.6: Pearson (r), Spearman (*ho*) and Harmonic score (h) obtained by the OurWE similarity methods evaluated herein.

	BIOSSES			MedSTS			CTR			Avg
	r	ρ	h	r	ρ	h	r	ρ	h	Avg
TOK.bioc_sg_defchar__tok.corenlp_lc_sw.user_cf.def_ner.none_Min	0.814	0.777	0.795	0.758	0.660	0.706	0.761	0.760	0.760	0.754
TOK.bioc_sg_defchar__tok.corenlp_lc_sw.user_cf.def_ner.none_Max	0.821	0.794	0.807	0.731	0.624	0.674	0.751	0.745	0.748	0.743
SW.bioc_sg_defchar__tok.whitesp_lc_sw.user_cf.def_ner.none_Min.2	0.746	0.734	0.740	0.755	0.677	0.714	0.771	0.761	0.766	0.740
CF.bioc_sg_defchar__tok.whitesp_lc_sw.user_cf.def_ner.none_Min	0.746	0.734	0.740	0.755	0.677	0.714	0.771	0.761	0.766	0.740
TOK.bioc_sg_defchar__tok.whitesp_lc_sw.user_cf.def_ner.none_Min	0.746	0.734	0.740	0.755	0.677	0.714	0.771	0.761	0.766	0.740
LC.bioc_sg_defchar__tok.whitesp_lc_sw.user_cf.def_ner.none_Min	0.746	0.734	0.740	0.755	0.677	0.714	0.771	0.761	0.766	0.740
SW.bioc_sg_defchar__tok.whitesp_lc_sw.user_cf.def_ner.none_Min.1	0.721	0.723	0.722	0.749	0.674	0.709	0.755	0.762	0.759	0.730
SW.bioc_sg_defchar__tok.whitesp_lc_sw.user_cf.def_ner.none_Min	0.721	0.722	0.721	0.748	0.673	0.709	0.755	0.761	0.758	0.729
TOK.bioc_sg_defchar__tok.bioc_lc_sw.user_cf.def_ner.none_Min	0.743	0.738	0.741	0.687	0.661	0.673	0.771	0.761	0.766	0.727
SW.bioc_sg_defchar__tok.whitesp_lc_sw.user_cf.def_ner.none_Max.2	0.706	0.700	0.703	0.732	0.641	0.683	0.770	0.760	0.765	0.717
CF.bioc_sg_defchar__tok.whitesp_lc_sw.user_cf.def_ner.none_Max	0.706	0.700	0.703	0.732	0.641	0.683	0.770	0.760	0.765	0.717
TOK.bioc_sg_defchar__tok.whitesp_lc_sw.user_cf.def_ner.none_Max	0.706	0.700	0.703	0.732	0.641	0.683	0.770	0.760	0.765	0.717
LC.bioc_sg_defchar__tok.whitesp_lc_sw.user_cf.def_ner.none_Max	0.706	0.700	0.703	0.732	0.641	0.683	0.770	0.760	0.765	0.717
SW.bioc_sg_defchar__tok.whitesp_lc_sw.user_cf.def_ner.none_Avg.1	0.657	0.684	0.670	0.753	0.660	0.703	0.723	0.808	0.763	0.712
SW.bioc_sg_defchar__tok.whitesp_lc_sw.user_cf.def_ner.none_Sum.1	0.657	0.684	0.670	0.753	0.660	0.703	0.723	0.808	0.763	0.712
SW.bioc_sg_defchar__tok.whitesp_lc_sw.user_cf.def_ner.none_Avg	0.658	0.676	0.667	0.753	0.658	0.702	0.724	0.807	0.763	0.711
SW.bioc_sg_defchar__tok.whitesp_lc_sw.user_cf.def_ner.none_Sum	0.658	0.676	0.667	0.753	0.658	0.702	0.724	0.807	0.763	0.711
SW.bioc_sg_defchar__tok.whitesp_lc_sw.user_cf.def_ner.none_Max	0.690	0.690	0.690	0.727	0.635	0.678	0.755	0.752	0.754	0.707
SW.bioc_sg_defchar__tok.whitesp_lc_sw.user_cf.def_ner.none_Max.1	0.687	0.688	0.688	0.727	0.635	0.678	0.755	0.756	0.756	0.707
TOK.bioc_sg_defchar__tok.bioc_lc_sw.user_cf.def_ner.none_Max	0.710	0.701	0.705	0.660	0.626	0.643	0.770	0.760	0.765	0.704
CF.bioc_sg_defchar__tok.whitesp_lc_sw.user_cf.biosses_ner.none_Max	0.733	0.767	0.750	0.655	0.606	0.629	0.605	0.608	0.606	0.662
TOK.bioc_sg_defchar__tok.corenlp_lc_sw.user_cf.def_ner.none_Avg	0.616	0.669	0.642	0.695	0.641	0.667	0.623	0.728	0.671	0.660
TOK.bioc_sg_defchar__tok.corenlp_lc_sw.user_cf.def_ner.none_Sum	0.616	0.669	0.642	0.695	0.641	0.667	0.623	0.728	0.671	0.660
CF.bioc_sg_defchar__tok.whitesp_lc_sw.user_cf.biosses_ner.none_Avg	0.621	0.663	0.641	0.661	0.629	0.645	0.606	0.704	0.651	0.646
CF.bioc_sg_defchar__tok.whitesp_lc_sw.user_cf.biosses_ner.none_Sum	0.621	0.663	0.641	0.661	0.629	0.645	0.606	0.704	0.651	0.646
SW.bioc_sg_defchar__tok.whitesp_lc_sw.user_cf.def_ner.none_Avg.2	0.568	0.616	0.591	0.679	0.645	0.662	0.613	0.719	0.662	0.638
SW.bioc_sg_defchar__tok.whitesp_lc_sw.user_cf.def_ner.none_Sum.2	0.568	0.616	0.591	0.679	0.645	0.662	0.613	0.719	0.662	0.638
CF.bioc_sg_defchar__tok.whitesp_lc_sw.user_cf.def_ner.none_Avg	0.568	0.616	0.591	0.679	0.645	0.662	0.613	0.719	0.662	0.638
CF.bioc_sg_defchar__tok.whitesp_lc_sw.user_cf.def_ner.none_Sum	0.568	0.616	0.591	0.679	0.645	0.662	0.613	0.719	0.662	0.638
TOK.bioc_sg_defchar__tok.whitesp_lc_sw.user_cf.def_ner.none_Avg	0.568	0.616	0.591	0.679	0.645	0.662	0.613	0.719	0.662	0.638
TOK.bioc_sg_defchar__tok.whitesp_lc_sw.user_cf.def_ner.none_Sum	0.568	0.616	0.591	0.679	0.645	0.662	0.613	0.719	0.662	0.638
LC.bioc_sg_defchar__tok.whitesp_lc_sw.user_cf.def_ner.none_Avg	0.568	0.616	0.591	0.679	0.645	0.662	0.613	0.719	0.662	0.638
LC.bioc_sg_defchar__tok.whitesp_lc_sw.user_cf.def_ner.none_Sum	0.568	0.616	0.591	0.679	0.645	0.662	0.613	0.719	0.662	0.638
TOK.bioc_sg_defchar__tok.bioc_lc_sw.user_cf.def_ner.none_Avg	0.579	0.630	0.603	0.556	0.632	0.592	0.613	0.719	0.662	0.619
TOK.bioc_sg_defchar__tok.bioc_lc_sw.user_cf.def_ner.none_Sum	0.579	0.630	0.603	0.556	0.632	0.592	0.613	0.719	0.662	0.619
CF.bioc_sg_defchar__tok.whitesp_lc_sw.user_cf.biosses_ner.none_Min	0.623	0.746	0.679	0.634	0.637	0.635	0.488	0.592	0.535	0.616
CF.bioc_sg_defchar__tok.whitesp_lc_sw.user_cf.blagec_ner.none_Avg	0.652	0.672	0.662	0.658	0.633	0.645	0.398	0.463	0.428	0.578
CF.bioc_sg_defchar__tok.whitesp_lc_sw.user_cf.blagec_ner.none_Sum	0.652	0.672	0.662	0.658	0.633	0.645	0.398	0.463	0.428	0.578
CF.bioc_sg_defchar__tok.whitesp_lc_sw.user_cf.blagec_ner.none_Max	0.669	0.749	0.707	0.615	0.597	0.606	0.244	0.269	0.256	0.523
LC.bioc_sg_defchar__tok.whitesp_notlc_sw.user_cf.def_ner.none_Avg	0.396	0.484	0.435	0.330	0.504	0.399	0.532	0.643	0.582	0.472
LC.bioc_sg_defchar__tok.whitesp_notlc_sw.user_cf.def_ner.none_Sum	0.396	0.484	0.435	0.330	0.504	0.399	0.532	0.643	0.582	0.472
CF.bioc_sg_defchar__tok.whitesp_lc_sw.user_cf.blagec_ner.none_Min	0.586	0.726	0.648	0.581	0.607	0.594	0.160	0.191	0.174	0.472
CF.bioc_sg_defchar__tok.whitesp_lc_sw.user_cf.none_ner.none_Avg	0.432	0.542	0.481	0.416	0.521	0.463	0.266	0.338	0.298	0.414
CF.bioc_sg_defchar__tok.whitesp_lc_sw.user_cf.none_ner.none_Sum	0.432	0.542	0.481	0.416	0.521	0.463	0.266	0.338	0.298	0.414
LC.bioc_sg_defchar__tok.whitesp_notlc_sw.user_cf.def_ner.none_Max	0.361	0.335	0.348	0.280	0.333	0.305	0.462	0.476	0.469	0.374
CF.bioc_sg_defchar__tok.whitesp_lc_sw.user_cf.none_ner.none_Max	0.347	0.501	0.410	0.411	0.448	0.429	0.236	0.297	0.263	0.367
CF.bioc_sg_defchar__tok.whitesp_lc_sw.user_cf.none_ner.none_Min	0.333	0.496	0.398	0.369	0.450	0.406	0.189	0.272	0.223	0.342
LC.bioc_sg_defchar__tok.whitesp_notlc_sw.user_cf.def_ner.none_Min	0.251	0.244	0.247	0.238	0.312	0.270	0.364	0.362	0.363	0.293

Table A.7: Table : Pearson (r), Spearman (*ho*) and Harmonic score (h) obtained by the WBSM similarity methods evaluated herein.

	BIOSSES			MedSTS			CTR			Avg
	r	ρ	h	r	ρ	h	r	ρ	h	Avg
WBSM_Rada_tok.corenlp_2_0_lc_sw.nltk_cf.biosses_ner.none	0.772	0.791	0.782	0.774	0.709	0.740	0.785	0.765	0.775	0.766
SW.WBSM_Rada_tok.whitesp_lc_sw.nltk_cf.def_ner.none	0.743	0.769	0.756	0.762	0.700	0.730	0.786	0.755	0.770	0.752
SW.WBSM_Rada_tok.whitesp_lc_sw.biosses_cf.def_ner.none	0.738	0.770	0.753	0.763	0.700	0.731	0.787	0.753	0.770	0.751
TOK.WBSM_Rada_tok.corenlp_2_0_lc_sw.none_cf.def_ner.none	0.723	0.765	0.743	0.753	0.702	0.726	0.726	0.723	0.725	0.731
CF.WBSM_Rada_tok.whitesp_lc_sw.none_cf.biosses_ner.none	0.694	0.733	0.713	0.741	0.691	0.715	0.720	0.712	0.716	0.715
SW.WBSM_Rada_tok.whitesp_lc_sw.none_cf.def_ner.none	0.687	0.730	0.708	0.742	0.692	0.716	0.721	0.715	0.718	0.714
CF.WBSM_Rada_tok.whitesp_lc_sw.none_cf.def_ner.none	0.687	0.730	0.708	0.742	0.692	0.716	0.721	0.715	0.718	0.714
TOK.WBSM_Rada_tok.whitesp_lc_sw.none_cf.def_ner.none	0.687	0.730	0.708	0.742	0.692	0.716	0.721	0.715	0.718	0.714
LC.WBSM_Rada_tok.whitesp_lc_sw.none_cf.def_ner.none	0.687	0.730	0.708	0.742	0.692	0.716	0.721	0.715	0.718	0.714
TOK.WBSM_Rada_tok.bioc_lc_sw.none_cf.def_ner.none	0.690	0.733	0.711	0.722	0.677	0.699	0.721	0.715	0.718	0.709
CF.WBSM_Rada_tok.whitesp_lc_sw.none_cf.blagec_ner.none	0.673	0.717	0.695	0.741	0.692	0.716	0.685	0.687	0.686	0.699
LC.WBSM_Rada_tok.whitesp_notlc_sw.none_cf.def_ner.none	0.646	0.685	0.665	0.703	0.657	0.679	0.712	0.702	0.707	0.684
CF.WBSM_Rada_tok.whitesp_lc_sw.none_cf.none_ner.none	0.609	0.641	0.625	0.701	0.662	0.681	0.692	0.688	0.690	0.665
TOK.WBSM_CosNWJC_tok.corenlp_2_0_lc_sw.none_cf.def_ner.none	0.569	0.607	0.587	0.688	0.645	0.666	0.615	0.601	0.608	0.620
WBSM_CosNWJC_tok.corenlp_2_0_lc_sw.nltk_cf.biosses_ner.none	0.571	0.566	0.568	0.705	0.651	0.677	0.637	0.590	0.613	0.619
CF.WBSM_CosNWJC_tok.whitesp_lc_sw.none_cf.biosses_ner.none	0.548	0.606	0.575	0.675	0.622	0.647	0.614	0.595	0.604	0.609
SW.WBSM_CosNWJC_tok.whitesp_lc_sw.nltk_cf.def_ner.none	0.538	0.560	0.549	0.689	0.630	0.658	0.641	0.590	0.615	0.607
SW.WBSM_CosNWJC_tok.whitesp_lc_sw.none_cf.def_ner.none	0.541	0.599	0.569	0.673	0.625	0.648	0.614	0.594	0.604	0.607
CF.WBSM_CosNWJC_tok.whitesp_lc_sw.none_cf.def_ner.none	0.541	0.599	0.569	0.673	0.625	0.648	0.614	0.594	0.604	0.607
TOK.WBSM_CosNWJC_tok.whitesp_lc_sw.none_cf.def_ner.none	0.541	0.599	0.569	0.673	0.625	0.648	0.614	0.594	0.604	0.607
LC.WBSM_CosNWJC_tok.whitesp_lc_sw.none_cf.def_ner.none	0.541	0.599	0.569	0.673	0.625	0.648	0.614	0.594	0.604	0.607
SW.WBSM_CosNWJC_tok.whitesp_lc_sw.biosses_cf.def_ner.none	0.535	0.556	0.545	0.690	0.633	0.660	0.638	0.592	0.614	0.607
TOK.WBSM_CosNWJC_tok.bioc_lc_sw.none_cf.def_ner.none	0.546	0.601	0.572	0.654	0.609	0.631	0.614	0.594	0.604	0.602
CF.WBSM_CosNWJC_tok.whitesp_lc_sw.none_cf.blagec_ner.none	0.530	0.581	0.554	0.674	0.622	0.647	0.586	0.579	0.582	0.594
LC.WBSM_CosNWJC_tok.whitesp_notlc_sw.none_cf.def_ner.none	0.494	0.547	0.519	0.630	0.594	0.611	0.598	0.576	0.587	0.572
CF.WBSM_CosNWJC_tok.whitesp_lc_sw.none_cf.none_ner.none	0.462	0.509	0.484	0.651	0.617	0.633	0.597	0.585	0.591	0.570
TOK.WBSM_WJC_tok.corenlp_2_0_lc_sw.none_cf.def_ner.none	0.480	0.548	0.512	0.647	0.615	0.631	0.536	0.515	0.525	0.556
TOK.WBSM_JC_tok.corenlp_2_0_lc_sw.none_cf.def_ner.none	0.480	0.548	0.512	0.647	0.615	0.631	0.536	0.515	0.525	0.556
CF.WBSM_JC_tok.whitesp_lc_sw.none_cf.biosses_ner.none	0.458	0.513	0.484	0.633	0.589	0.610	0.537	0.514	0.525	0.540
CF.WBSM_WJC_tok.whitesp_lc_sw.none_cf.biosses_ner.none	0.458	0.513	0.484	0.633	0.589	0.610	0.537	0.514	0.525	0.540
WBSM_JC_tok.corenlp_2_0_lc_sw.nltk_cf.biosses_ner.none	0.452	0.454	0.453	0.660	0.620	0.639	0.540	0.503	0.521	0.538
WBSM_WJC_tok.corenlp_2_0_lc_sw.nltk_cf.biosses_ner.none	0.452	0.454	0.453	0.660	0.620	0.639	0.540	0.503	0.521	0.538
SW.WBSM_JC_tok.whitesp_lc_sw.none_cf.def_ner.none	0.450	0.504	0.476	0.630	0.592	0.611	0.537	0.515	0.526	0.537
CF.WBSM_JC_tok.whitesp_lc_sw.none_cf.def_ner.none	0.450	0.504	0.476	0.630	0.592	0.611	0.537	0.515	0.526	0.537
TOK.WBSM_JC_tok.whitesp_lc_sw.none_cf.def_ner.none	0.450	0.504	0.476	0.630	0.592	0.611	0.537	0.515	0.526	0.537
LC.WBSM_JC_tok.whitesp_lc_sw.none_cf.def_ner.none	0.450	0.504	0.476	0.630	0.592	0.611	0.537	0.515	0.526	0.537
SW.WBSM_WJC_tok.whitesp_lc_sw.none_cf.def_ner.none	0.450	0.504	0.476	0.630	0.592	0.611	0.537	0.515	0.526	0.537
CF.WBSM_WJC_tok.whitesp_lc_sw.none_cf.def_ner.none	0.450	0.504	0.476	0.630	0.592	0.611	0.537	0.515	0.526	0.537
TOK.WBSM_WJC_tok.whitesp_lc_sw.none_cf.def_ner.none	0.450	0.504	0.476	0.630	0.592	0.611	0.537	0.515	0.526	0.537
LC.WBSM_WJC_tok.whitesp_lc_sw.none_cf.def_ner.none	0.450	0.504	0.476	0.630	0.592	0.611	0.537	0.515	0.526	0.537
TOK.WBSM_JC_tok.bioc_lc_sw.none_cf.def_ner.none	0.456	0.514	0.483	0.615	0.578	0.596	0.537	0.515	0.526	0.535
TOK.WBSM_WJC_tok.bioc_lc_sw.none_cf.def_ner.none	0.456	0.514	0.483	0.615	0.578	0.596	0.537	0.515	0.526	0.535
CF.WBSM_JC_tok.whitesp_lc_sw.none_cf.blagec_ner.none	0.447	0.508	0.475	0.632	0.589	0.610	0.513	0.495	0.504	0.530
CF.WBSM_WJC_tok.whitesp_lc_sw.none_cf.blagec_ner.none	0.447	0.508	0.475	0.632	0.589	0.610	0.513	0.495	0.504	0.530
TOK.WBSM_Cai_tok.corenlp_2_0_lc_sw.none_cf.def_ner.none	0.455	0.535	0.492	0.628	0.603	0.615	0.494	0.459	0.476	0.527
SW.WBSM_JC_tok.whitesp_lc_sw.nltk_cf.def_ner.none	0.415	0.432	0.423	0.638	0.592	0.614	0.543	0.503	0.522	0.520
SW.WBSM_WJC_tok.whitesp_lc_sw.nltk_cf.def_ner.none	0.415	0.432	0.423	0.638	0.592	0.614	0.543	0.503	0.522	0.520
SW.WBSM_JC_tok.whitesp_lc_sw.biosses_cf.def_ner.none	0.417	0.430	0.423	0.640	0.594	0.616	0.540	0.500	0.519	0.520
SW.WBSM_WJC_tok.whitesp_lc_sw.biosses_cf.def_ner.none	0.417	0.430	0.423	0.640	0.594	0.616	0.540	0.500	0.519	0.520
CF.WBSM_Cai_tok.whitesp_lc_sw.none_cf.biosses_ner.none	0.434	0.513	0.470	0.614	0.576	0.594	0.498	0.461	0.478	0.514
SW.WBSM_Cai_tok.whitesp_lc_sw.none_cf.def_ner.none	0.424	0.507	0.462	0.610	0.579	0.594	0.499	0.459	0.478	0.511
CF.WBSM_Cai_tok.whitesp_lc_sw.none_cf.def_ner.none	0.424	0.507	0.462	0.610	0.579	0.594	0.499	0.459	0.478	0.511
TOK.WBSM_Cai_tok.whitesp_lc_sw.none_cf.def_ner.none	0.424	0.507	0.462	0.610	0.579	0.594	0.499	0.459	0.478	0.511
LC.WBSM_Cai_tok.whitesp_lc_sw.none_cf.def_ner.none	0.424	0.507	0.462	0.610	0.579	0.594	0.499	0.459	0.478	0.511
CF.WBSM_WJC_tok.whitesp_lc_sw.none_cf.none_ner.none	0.371	0.438	0.401	0.615	0.598	0.607	0.530	0.512	0.520	0.509
CF.WBSM_JC_tok.whitesp_lc_sw.none_cf.none_ner.none	0.371	0.438	0.401	0.615	0.598	0.607	0.530	0.512	0.520	0.509
TOK.WBSM_Cai_tok.bioc_lc_sw.none_cf.def_ner.none	0.429	0.509	0.466	0.598	0.565	0.581	0.499	0.459	0.478	0.508
CF.WBSM_Cai_tok.whitesp_lc_sw.none_cf.blagec_ner.none	0.426	0.506	0.463	0.612	0.575	0.593	0.473	0.439	0.455	0.504
LC.WBSM_WJC_tok.whitesp_notlc_sw.none_cf.def_ner.none	0.400	0.455	0.426	0.582	0.561	0.572	0.526	0.500	0.512	0.503
LC.WBSM_JC_tok.whitesp_notlc_sw.none_cf.def_ner.none	0.400	0.455	0.426	0.582	0.561	0.572	0.526	0.500	0.512	0.503
WBSM_Cai_tok.corenlp_2_0_lc_sw.nltk_cf.biosses_ner.none	0.407	0.449	0.427	0.637	0.604	0.620	0.483	0.443	0.462	0.503
CF.WBSM_Cai_tok.whitesp_lc_sw.none_cf.none_ner.none	0.339	0.422	0.376	0.598	0.592	0.595	0.491	0.458	0.474	0.482
SW.WBSM_Cai_tok.whitesp_lc_sw.nltk_cf.def_ner.none	0.373	0.393	0.383	0.613	0.574	0.593	0.488	0.445	0.465	0.480
SW.WBSM_Cai_tok.whitesp_lc_sw.biosses_cf.def_ner.none	0.377	0.391	0.384	0.614	0.576	0.595	0.482	0.443	0.462	0.480
LC.WBSM_Cai_tok.whitesp_notlc_sw.none_cf.def_ner.none	0.366	0.442	0.400	0.561	0.549	0.555	0.483	0.464	0.473	0.476

Table A.8: Table : Pearson (r), Spearman (*ho*) and Harmonic score (h) obtained by the UBSM similarity methods evaluated herein.

	BIOSSES			MedSTS			CTR			Avg
	r	ρ	h	r	ρ	h	r	ρ	h	Avg
SW.UBSM_Rada_tok.whitesp_lc_sw.nltk_cf.def_ner.ctakes	0.793	0.813	0.803	0.754	0.692	0.722	0.779	0.780	0.779	0.768
SW.UBSM_Rada_tok.whitesp_lc_sw.biosses_cf.def_ner.ctakes	0.788	0.808	0.798	0.755	0.693	0.723	0.777	0.776	0.777	0.766
TOK.UBSM_Rada_tok.corenlp_2_0_lc_sw.none_cf.def_ner.ctakes	0.728	0.780	0.753	0.738	0.692	0.714	0.717	0.720	0.718	0.729
CF.UBSM_Rada_tok.whitesp_lc_sw.none_cf.biosses_ner.ctakes	0.714	0.771	0.741	0.732	0.685	0.707	0.712	0.710	0.711	0.720
SW.UBSM_Rada_tok.whitesp_lc_sw.none_cf.def_ner.ctakes	0.706	0.765	0.734	0.731	0.685	0.707	0.713	0.712	0.713	0.718
CF.UBSM_Rada_tok.whitesp_lc_sw.none_cf.def_ner.ctakes	0.706	0.765	0.734	0.731	0.685	0.707	0.713	0.712	0.713	0.718
UBSM_Rada_tok.corenlp_2_0_lc_sw.nltk_cf.biosses_ner.metamap	0.729	0.755	0.742	0.712	0.651	0.680	0.729	0.736	0.732	0.718
TOK.UBSM_Rada_tok.whitesp_lc_sw.none_cf.def_ner.ctakes	0.706	0.765	0.734	0.731	0.685	0.707	0.713	0.712	0.713	0.718
LC.UBSM_Rada_tok.whitesp_lc_sw.none_cf.def_ner.ctakes	0.706	0.765	0.734	0.731	0.685	0.707	0.713	0.712	0.713	0.718
NER.UBSM_WJC_tok.whitesp_lc_sw.none_cf.def_ner.ctakes	0.702	0.767	0.733	0.733	0.689	0.710	0.709	0.707	0.708	0.717
NER.UBSM_Rada_tok.whitesp_lc_sw.none_cf.def_ner.ctakes	0.702	0.767	0.733	0.733	0.689	0.710	0.709	0.707	0.708	0.717
NER.UBSM_CosNWJC_tok.whitesp_lc_sw.none_cf.def_ner.ctakes	0.702	0.767	0.733	0.733	0.689	0.710	0.709	0.707	0.708	0.717
NER.UBSM_Cai_tok.whitesp_lc_sw.none_cf.def_ner.ctakes	0.702	0.767	0.733	0.733	0.689	0.710	0.709	0.707	0.708	0.717
NER.UBSM_JC_tok.whitesp_lc_sw.none_cf.def_ner.ctakes	0.702	0.767	0.733	0.733	0.689	0.710	0.709	0.707	0.708	0.717
TOK.UBSM_Rada_tok.bioc_lc_sw.none_cf.def_ner.ctakes	0.707	0.764	0.734	0.711	0.670	0.690	0.712	0.711	0.712	0.712
CF.UBSM_Rada_tok.whitesp_lc_sw.none_cf.blagec_ner.ctakes	0.700	0.759	0.728	0.730	0.683	0.706	0.673	0.676	0.674	0.703
LC.UBSM_Rada_tok.whitesp_notlc_sw.none_cf.def_ner.ctakes	0.670	0.730	0.699	0.698	0.656	0.677	0.700	0.693	0.697	0.691
SW.UBSM_CosNWJC_tok.whitesp_lc_sw.nltk_cf.def_ner.ctakes	0.697	0.731	0.713	0.683	0.610	0.644	0.706	0.660	0.682	0.680
SW.UBSM_CosNWJC_tok.whitesp_lc_sw.biosses_cf.def_ner.ctakes	0.694	0.729	0.711	0.685	0.612	0.646	0.704	0.656	0.679	0.679
TOK.UBSM_CosNWJC_tok.corenlp_2_0_lc_sw.none_cf.def_ner.ctakes	0.672	0.727	0.698	0.678	0.623	0.649	0.684	0.676	0.680	0.676
CF.UBSM_Rada_tok.whitesp_lc_sw.none_cf.none_ner.ctakes	0.639	0.685	0.661	0.699	0.655	0.676	0.679	0.675	0.677	0.671
CF.UBSM_CosNWJC_tok.whitesp_lc_sw.none_cf.biosses_ner.ctakes	0.644	0.696	0.669	0.670	0.613	0.641	0.674	0.661	0.668	0.659
NER.UBSM_WJC_tok.whitesp_lc_sw.none_cf.def_ner.metamap	0.649	0.701	0.674	0.672	0.644	0.657	0.646	0.644	0.645	0.659
NER.UBSM_Rada_tok.whitesp_lc_sw.none_cf.def_ner.metamap	0.649	0.701	0.674	0.672	0.644	0.657	0.646	0.644	0.645	0.659
NER.UBSM_CosNWJC_tok.whitesp_lc_sw.none_cf.def_ner.metamap	0.649	0.701	0.674	0.672	0.644	0.657	0.646	0.644	0.645	0.659
NER.UBSM_Cai_tok.whitesp_lc_sw.none_cf.def_ner.metamap	0.649	0.701	0.674	0.672	0.644	0.657	0.646	0.644	0.645	0.659
NER.UBSM_JC_tok.whitesp_lc_sw.none_cf.def_ner.metamap	0.649	0.701	0.674	0.672	0.644	0.657	0.646	0.644	0.645	0.659
SW.UBSM_CosNWJC_tok.whitesp_lc_sw.none_cf.def_ner.ctakes	0.635	0.693	0.663	0.670	0.614	0.640	0.676	0.664	0.670	0.658
CF.UBSM_CosNWJC_tok.whitesp_lc_sw.none_cf.def_ner.ctakes	0.635	0.693	0.663	0.670	0.614	0.640	0.676	0.664	0.670	0.658
TOK.UBSM_CosNWJC_tok.whitesp_lc_sw.none_cf.def_ner.ctakes	0.635	0.693	0.663	0.669	0.613	0.640	0.676	0.664	0.670	0.658
LC.UBSM_CosNWJC_tok.whitesp_lc_sw.none_cf.def_ner.ctakes	0.635	0.693	0.663	0.669	0.613	0.640	0.676	0.664	0.670	0.658
TOK.UBSM_CosNWJC_tok.bioc_lc_sw.none_cf.def_ner.ctakes	0.637	0.695	0.665	0.648	0.600	0.623	0.677	0.664	0.670	0.652
NER.UBSM_CosNWJC_tok.whitesp_lc_sw.none_cf.def_ner.metamaplite	0.578	0.619	0.598	0.707	0.654	0.680	0.679	0.679	0.679	0.652
CF.UBSM_CosNWJC_tok.whitesp_lc_sw.none_cf.blagec_ner.ctakes	0.631	0.684	0.656	0.670	0.612	0.640	0.641	0.634	0.638	0.645
TOK.UBSM_WJC_tok.corenlp_2_0_lc_sw.none_cf.def_ner.ctakes	0.621	0.672	0.645	0.645	0.585	0.613	0.648	0.630	0.639	0.632
LC.UBSM_CosNWJC_tok.whitesp_notlc_sw.none_cf.def_ner.ctakes	0.596	0.659	0.626	0.633	0.581	0.606	0.660	0.650	0.655	0.629
NER.UBSM_WJC_tok.whitesp_lc_sw.none_cf.def_ner.metamaplite	0.527	0.573	0.549	0.689	0.639	0.663	0.643	0.636	0.640	0.617
CF.UBSM_CosNWJC_tok.whitesp_lc_sw.none_cf.none_ner.ctakes	0.562	0.609	0.585	0.636	0.581	0.607	0.654	0.649	0.651	0.614
SW.UBSM_WJC_tok.whitesp_lc_sw.nltk_cf.def_ner.ctakes	0.611	0.656	0.633	0.643	0.565	0.602	0.638	0.581	0.608	0.614
UBSM_CosNWJC_tok.corenlp_2_0_lc_sw.nltk_cf.biosses_ner.metamap	0.645	0.660	0.653	0.676	0.606	0.639	0.571	0.528	0.549	0.614
SW.UBSM_WJC_tok.whitesp_lc_sw.biosses_cf.def_ner.ctakes	0.608	0.655	0.631	0.645	0.567	0.604	0.635	0.578	0.605	0.613
CF.UBSM_WJC_tok.whitesp_lc_sw.none_cf.biosses_ner.ctakes	0.582	0.629	0.604	0.638	0.576	0.605	0.635	0.620	0.627	0.612
SW.UBSM_WJC_tok.whitesp_lc_sw.none_cf.def_ner.ctakes	0.573	0.628	0.599	0.637	0.576	0.605	0.636	0.620	0.628	0.611
CF.UBSM_WJC_tok.whitesp_lc_sw.none_cf.def_ner.ctakes	0.573	0.628	0.599	0.637	0.576	0.605	0.636	0.620	0.628	0.611
TOK.UBSM_WJC_tok.whitesp_lc_sw.none_cf.def_ner.ctakes	0.573	0.628	0.599	0.637	0.576	0.605	0.636	0.620	0.628	0.611
LC.UBSM_WJC_tok.whitesp_lc_sw.none_cf.def_ner.ctakes	0.573	0.628	0.599	0.637	0.576	0.605	0.636	0.620	0.628	0.611
TOK.UBSM_WJC_tok.bioc_lc_sw.none_cf.def_ner.ctakes	0.575	0.629	0.601	0.615	0.563	0.588	0.639	0.627	0.633	0.607
CF.UBSM_WJC_tok.whitesp_lc_sw.none_cf.blagec_ner.ctakes	0.569	0.618	0.593	0.637	0.575	0.605	0.606	0.600	0.603	0.600
TOK.UBSM_Cai_tok.corenlp_2_0_lc_sw.none_cf.def_ner.ctakes	0.575	0.629	0.601	0.624	0.558	0.589	0.611	0.597	0.604	0.598
TOK.UBSM_JC_tok.corenlp_2_0_lc_sw.none_cf.def_ner.ctakes	0.564	0.612	0.587	0.622	0.551	0.585	0.590	0.578	0.584	0.585
LC.UBSM_WJC_tok.whitesp_notlc_sw.none_cf.def_ner.ctakes	0.534	0.591	0.561	0.599	0.542	0.569	0.621	0.609	0.615	0.582
CF.UBSM_Cai_tok.whitesp_lc_sw.none_cf.biosses_ner.ctakes	0.527	0.579	0.552	0.617	0.549	0.581	0.596	0.579	0.587	0.573
CF.UBSM_WJC_tok.whitesp_lc_sw.none_cf.none_ner.ctakes	0.502	0.548	0.524	0.604	0.546	0.573	0.624	0.618	0.621	0.573
TOK.UBSM_Cai_tok.whitesp_lc_sw.none_cf.def_ner.ctakes	0.520	0.576	0.546	0.616	0.549	0.581	0.597	0.580	0.588	0.572
LC.UBSM_Cai_tok.whitesp_lc_sw.none_cf.def_ner.ctakes	0.520	0.576	0.546	0.616	0.549	0.581	0.597	0.580	0.588	0.572
SW.UBSM_Cai_tok.whitesp_lc_sw.none_cf.def_ner.ctakes	0.520	0.576	0.546	0.616	0.549	0.581	0.597	0.580	0.588	0.572
CF.UBSM_Cai_tok.whitesp_lc_sw.none_cf.def_ner.ctakes	0.520	0.576	0.546	0.616	0.549	0.581	0.597	0.580	0.588	0.572
TOK.UBSM_Cai_tok.bioc_lc_sw.none_cf.def_ner.ctakes	0.521	0.578	0.548	0.593	0.536	0.563	0.603	0.592	0.597	0.569
CF.UBSM_Cai_tok.whitesp_lc_sw.none_cf.blagec_ner.ctakes	0.516	0.566	0.540	0.617	0.549	0.581	0.571	0.568	0.570	0.563
SW.UBSM_Cai_tok.whitesp_lc_sw.nltk_cf.def_ner.ctakes	0.540	0.592	0.565	0.618	0.532	0.572	0.576	0.525	0.550	0.562
CF.UBSM_JC_tok.whitesp_lc_sw.none_cf.biosses_ner.ctakes	0.520	0.558	0.538	0.615	0.543	0.576	0.574	0.566	0.570	0.562
SW.UBSM_Cai_tok.whitesp_lc_sw.biosses_cf.def_ner.ctakes	0.539	0.584	0.561	0.620	0.534	0.574	0.574	0.523	0.547	0.561
SW.UBSM_JC_tok.whitesp_lc_sw.none_cf.def_ner.ctakes	0.512	0.557	0.534	0.614	0.542	0.576	0.575	0.568	0.571	0.560
CF.UBSM_JC_tok.whitesp_lc_sw.none_cf.def_ner.ctakes	0.512	0.557	0.534	0.614	0.542	0.576	0.575	0.568	0.571	0.560
TOK.UBSM_JC_tok.whitesp_lc_sw.none_cf.def_ner.ctakes	0.512	0.557	0.534	0.614	0.542	0.576	0.575	0.568	0.571	0.560
LC.UBSM_JC_tok.whitesp_lc_sw.none_cf.def_ner.ctakes	0.512	0.557	0.534	0.614	0.542	0.576	0.575	0.568	0.571	0.560
TOK.UBSM_JC_tok.bioc_lc_sw.none_cf.def_ner.ctakes	0.514	0.558	0.535	0.591	0.530	0.559	0.582	0.581	0.581	0.558
CF.UBSM_JC_tok.whitesp_lc_sw.none_cf.blagec_ner.ctakes	0.509	0.552	0.530	0.615	0.542	0.576	0.549	0.546	0.548	0.551
SW.UBSM_JC_tok.whitesp_lc_sw.nltk_cf.def_ner.ctakes	0.535	0.578	0.556	0.613	0.524	0.565	0.545	0.515	0.530	0.550
SW.UBSM_JC_tok.whitesp_lc_sw.biosses_cf.def_ner.ctakes	0.534	0.575	0.554	0.615	0.525	0.567	0.543	0.515	0.528	0.549
LC.UBSM_Cai_tok.whitesp_notlc_sw.none_cf.def_ner.ctakes	0.478	0.527	0.501	0.577	0.515	0.544	0.583	0.573	0.578	0.541
CF.UBSM_Cai_tok.whitesp_lc_sw.none_cf.none_ner.ctakes	0.451	0.511	0.479	0.583	0.521	0.551	0.592	0.583	0.587	0.539
LC.UBSM_JC_tok.whitesp_notlc_sw.none_cf.def_ner.ctakes	0.469	0.519	0.493	0.575	0.507	0.539	0.561	0.556	0.558	0.530
CF.UBSM_JC_tok.whitesp_lc_sw.none_cf.none_ner.ctakes	0.448	0.502	0.473	0.581	0.514	0.545	0.568	0.569	0.568	0.529
UBSM_WJC_tok.corenlp_2_0_lc_sw.nltk_cf.biosses_ner.metamap	0.545	0.534	0.539	0.639	0.574	0.605	0.443	0.416	0.429	0.524
NER.UBSM_JC_tok.whitesp_lc_sw.none_cf.def_ner.metamaplite	0.375	0.428	0.400	0.646	0.598	0.621	0.536	0.523	0.530	0.517
NER.UBSM_Cai_tok.whitesp_lc_sw.none_cf.def_ner.metamaplite	0.361	0.422	0.389	0.642	0.599	0.620	0.527	0.514	0.520	0.510
NER.UBSM_Rada_tok.whitesp_lc_sw.none_cf.def_ner.metamaplite	0.341	0.374	0.357	0.672	0.626	0.648	0.511	0.505	0.508	0.504
UBSM_Cai_tok.corenlp_2_0_lc_sw.nltk_cf.biosses_ner.metamap	0.429	0.438	0.433	0.609	0.557	0.582	0.312	0.316	0.314	0.443
UBSM_JC_tok.corenlp_2_0_lc_sw.nltk_cf.biosses_ner.metamap	0.313	0.326	0.320	0.580	0.547	0.563	0.232	0.230	0.231	0.371

Table A.9: Part 1 of 2: Pearson (r), Spearman (*ho*) and Harmonic score (h) obtained by the SWEM similarity methods evaluated herein.

	BIOSSES			MedSTS			CTR			Avg
	r	ρ	h	r	ρ	h	r	ρ	h	Avg
TOK.bio_emb_int__tok.corenlp_lc_sw.none_cf.def_ner.none_Min	0.831	0.809	0.820	0.764	0.682	0.721	0.761	0.736	0.748	0.763
CF.bio_emb_int__tok.whitesp_lc_sw.none_cf.biosses_ner.none_Min	0.819	0.797	0.808	0.756	0.685	0.719	0.760	0.729	0.744	0.757
CF.bio_emb_int__tok.whitesp_lc_sw.none_cf.blagec_ner.none_Min	0.811	0.782	0.796	0.765	0.696	0.729	0.760	0.729	0.744	0.756
TOK.bio_emb_int__tok.corenlp_lc_sw.none_cf.def_ner.none_Max	0.829	0.793	0.811	0.759	0.665	0.709	0.757	0.724	0.740	0.753
CF.bio_emb_int__tok.whitesp_lc_sw.none_cf.biosses_ner.none_Max	0.822	0.794	0.808	0.753	0.668	0.708	0.762	0.724	0.743	0.753
CF.bio_emb_int__tok.whitesp_lc_sw.none_cf.blagec_ner.none_Max	0.797	0.766	0.781	0.760	0.681	0.718	0.762	0.724	0.743	0.747
SW.bio_emb_int__tok.whitesp_lc_sw.none_cf.def_ner.none_Min	0.755	0.737	0.746	0.760	0.692	0.724	0.758	0.729	0.743	0.738
CF.bio_emb_int__tok.whitesp_lc_sw.none_cf.def_ner.none_Min	0.755	0.737	0.746	0.760	0.692	0.724	0.758	0.729	0.743	0.738
TOK.bio_emb_int__tok.whitesp_lc_sw.none_cf.def_ner.none_Min	0.755	0.737	0.746	0.760	0.692	0.724	0.758	0.729	0.743	0.738
LC.bio_emb_int__tok.whitesp_lc_sw.none_cf.def_ner.none_Min	0.755	0.737	0.746	0.760	0.692	0.724	0.758	0.729	0.743	0.738
CF.bio_emb_int__tok.whitesp_lc_sw.none_cf.none_ner.none_Min	0.782	0.741	0.761	0.688	0.644	0.665	0.773	0.742	0.757	0.728
TOK.bio_emb_int__tok.bioc_lc_sw.none_cf.def_ner.none_Min	0.756	0.740	0.748	0.705	0.676	0.690	0.758	0.729	0.743	0.727
SW.bio_emb_int__tok.whitesp_lc_sw.biosses_cf.def_ner.none_Min	0.720	0.712	0.716	0.755	0.687	0.720	0.752	0.733	0.742	0.726
SW.bio_emb_int__tok.whitesp_lc_sw.none_cf.def_ner.none_Max	0.721	0.726	0.723	0.755	0.672	0.711	0.759	0.727	0.743	0.726
CF.bio_emb_int__tok.whitesp_lc_sw.none_cf.def_ner.none_Max	0.721	0.726	0.723	0.755	0.672	0.711	0.759	0.727	0.743	0.726
TOK.bio_emb_int__tok.whitesp_lc_sw.none_cf.def_ner.none_Max	0.721	0.726	0.723	0.755	0.672	0.711	0.759	0.727	0.743	0.726
LC.bio_emb_int__tok.whitesp_lc_sw.none_cf.def_ner.none_Max	0.721	0.726	0.723	0.755	0.672	0.711	0.759	0.727	0.743	0.726
SW.bio_emb_int__tok.whitesp_lc_sw.nltk_cf.def_ner.none_Min	0.720	0.714	0.717	0.754	0.687	0.719	0.750	0.728	0.739	0.725
SW.bio_emb_int__tok.whitesp_lc_sw.biosses_cf.def_ner.none_Max	0.690	0.712	0.701	0.749	0.671	0.707	0.750	0.722	0.736	0.715
SW.bio_emb_int__tok.whitesp_lc_sw.nltk_cf.def_ner.none_Max	0.692	0.715	0.703	0.747	0.669	0.706	0.749	0.719	0.734	0.714
SW.PubMed.PMC.w2v__tok.whitesp_lc_sw.nltk_cf.def_ner.none_Avg	0.683	0.659	0.671	0.752	0.650	0.697	0.743	0.803	0.772	0.713
SW.PubMed.PMC.w2v__tok.whitesp_lc_sw.nltk_cf.def_ner.none_Sum	0.683	0.659	0.671	0.752	0.650	0.697	0.743	0.803	0.772	0.713
TOK.bio_emb_int__tok.bioc_lc_sw.none_cf.def_ner.none_Max	0.717	0.721	0.719	0.695	0.656	0.675	0.759	0.727	0.743	0.712
SW.PubMed.PMC.w2v__tok.whitesp_lc_sw.biosses_cf.def_ner.none_Avg	0.678	0.653	0.666	0.752	0.650	0.697	0.742	0.804	0.772	0.712
SW.PubMed.PMC.w2v__tok.whitesp_lc_sw.biosses_cf.def_ner.none_Sum	0.678	0.653	0.666	0.752	0.650	0.697	0.742	0.804	0.772	0.712
CF.bio_emb_int__tok.whitesp_lc_sw.none_cf.none_ner.none_Max	0.748	0.704	0.726	0.674	0.621	0.646	0.774	0.740	0.757	0.709
SW.bio_emb_int__tok.whitesp_lc_sw.biosses_cf.def_ner.none_Avg	0.661	0.679	0.670	0.760	0.667	0.711	0.703	0.750	0.725	0.702
SW.bio_emb_int__tok.whitesp_lc_sw.biosses_cf.def_ner.none_Sum	0.661	0.679	0.670	0.760	0.667	0.711	0.703	0.750	0.725	0.702
SW.bio_emb_int__tok.whitesp_lc_sw.nltk_cf.def_ner.none_Avg	0.663	0.678	0.670	0.759	0.665	0.709	0.703	0.748	0.725	0.701
SW.bio_emb_int__tok.whitesp_lc_sw.nltk_cf.def_ner.none_Sum	0.663	0.678	0.670	0.759	0.665	0.709	0.703	0.748	0.725	0.701
TOK.PubMed.PMC.w2v__tok.corenlp_lc_sw.none_cf.def_ner.none_Avg	0.628	0.681	0.653	0.718	0.634	0.673	0.665	0.746	0.703	0.677
TOK.PubMed.PMC.w2v__tok.corenlp_lc_sw.none_cf.def_ner.none_Sum	0.628	0.681	0.653	0.718	0.634	0.673	0.665	0.746	0.703	0.677
SW.BioNLP.win.2__tok.whitesp_lc_sw.nltk_cf.def_ner.none_Avg	0.577	0.608	0.592	0.747	0.640	0.690	0.689	0.736	0.711	0.665
SW.BioNLP.win.2__tok.whitesp_lc_sw.nltk_cf.def_ner.none_Sum	0.577	0.608	0.592	0.747	0.640	0.690	0.689	0.736	0.711	0.665
TOK.bio_emb_int__tok.corenlp_lc_sw.none_cf.def_ner.none_Avg	0.654	0.673	0.663	0.688	0.644	0.665	0.626	0.695	0.659	0.662
TOK.bio_emb_int__tok.corenlp_lc_sw.none_cf.def_ner.none_Sum	0.654	0.673	0.663	0.688	0.644	0.665	0.626	0.695	0.659	0.662
SW.BioNLP.win.2__tok.whitesp_lc_sw.biosses_cf.def_ner.none_Avg	0.573	0.609	0.590	0.748	0.641	0.691	0.680	0.732	0.705	0.662
SW.BioNLP.win.2__tok.whitesp_lc_sw.biosses_cf.def_ner.none_Sum	0.573	0.609	0.590	0.748	0.641	0.691	0.680	0.732	0.705	0.662
SW.PubMed.PMC.w2v__tok.whitesp_lc_sw.none_cf.def_ner.none_Avg	0.599	0.633	0.616	0.711	0.638	0.673	0.654	0.746	0.697	0.662
CF.PubMed.PMC.w2v__tok.whitesp_lc_sw.none_cf.def_ner.none_Avg	0.599	0.633	0.616	0.711	0.638	0.673	0.654	0.746	0.697	0.662
TOK.PubMed.PMC.w2v__tok.whitesp_lc_sw.none_cf.def_ner.none_Avg	0.599	0.633	0.616	0.711	0.638	0.673	0.654	0.746	0.697	0.662
LC.PubMed.PMC.w2v__tok.whitesp_lc_sw.none_cf.def_ner.none_Avg	0.599	0.633	0.616	0.711	0.638	0.673	0.654	0.746	0.697	0.662
SW.PubMed.PMC.w2v__tok.whitesp_lc_sw.none_cf.def_ner.none_Sum	0.599	0.633	0.616	0.711	0.638	0.673	0.654	0.746	0.697	0.662
CF.PubMed.PMC.w2v__tok.whitesp_lc_sw.none_cf.def_ner.none_Sum	0.599	0.633	0.616	0.711	0.638	0.673	0.654	0.746	0.697	0.662
TOK.PubMed.PMC.w2v__tok.whitesp_lc_sw.none_cf.def_ner.none_Sum	0.599	0.633	0.616	0.711	0.638	0.673	0.654	0.746	0.697	0.662
LC.PubMed.PMC.w2v__tok.whitesp_lc_sw.none_cf.def_ner.none_Sum	0.599	0.633	0.616	0.711	0.638	0.673	0.654	0.746	0.697	0.662
TOK.PubMed.PMC.w2v__tok.corenlp_lc_sw.none_cf.def_ner.none_Max	0.663	0.663	0.663	0.665	0.587	0.624	0.677	0.708	0.692	0.660
CF.PubMed.PMC.w2v__tok.whitesp_lc_sw.none_cf.biosses_ner.none_Avg	0.594	0.618	0.606	0.711	0.638	0.672	0.652	0.738	0.692	0.657
CF.PubMed.PMC.w2v__tok.whitesp_lc_sw.none_cf.biosses_ner.none_Sum	0.594	0.618	0.606	0.711	0.638	0.672	0.652	0.738	0.692	0.657
TOK.PubMed.PMC.w2v__tok.bioc_lc_sw.none_cf.def_ner.none_Avg	0.602	0.637	0.619	0.664	0.624	0.643	0.654	0.746	0.697	0.653
TOK.PubMed.PMC.w2v__tok.bioc_lc_sw.none_cf.def_ner.none_Sum	0.602	0.637	0.619	0.664	0.624	0.643	0.654	0.746	0.697	0.653
CF.bio_emb_int__tok.whitesp_lc_sw.none_cf.biosses_ner.none_Avg	0.629	0.657	0.643	0.674	0.648	0.661	0.613	0.690	0.649	0.651
CF.bio_emb_int__tok.whitesp_lc_sw.none_cf.biosses_ner.none_Sum	0.629	0.657	0.643	0.674	0.648	0.661	0.613	0.690	0.649	0.651
LC.PubMed.PMC.w2v__tok.whitesp_notlc_sw.none_cf.def_ner.none_Avg	0.630	0.647	0.638	0.632	0.577	0.604	0.666	0.760	0.710	0.651
LC.PubMed.PMC.w2v__tok.whitesp_notlc_sw.none_cf.def_ner.none_Sum	0.630	0.647	0.638	0.632	0.577	0.604	0.666	0.760	0.710	0.651
CF.bio_emb_int__tok.whitesp_lc_sw.none_cf.blagec_ner.none_Avg	0.619	0.645	0.632	0.678	0.651	0.664	0.613	0.690	0.649	0.648
CF.bio_emb_int__tok.whitesp_lc_sw.none_cf.blagec_ner.none_Sum	0.619	0.645	0.632	0.678	0.651	0.664	0.613	0.690	0.649	0.648
CF.PubMed.PMC.w2v__tok.whitesp_lc_sw.none_cf.blagec_ner.none_Avg	0.590	0.605	0.597	0.713	0.641	0.675	0.627	0.711	0.666	0.646
CF.PubMed.PMC.w2v__tok.whitesp_lc_sw.none_cf.blagec_ner.none_Sum	0.590	0.605	0.597	0.713	0.641	0.675	0.627	0.711	0.666	0.646
TOK.PubMed.PMC.w2v__tok.corenlp_lc_sw.none_cf.def_ner.none_Min	0.631	0.632	0.631	0.664	0.578	0.618	0.652	0.674	0.663	0.637
SW.bio_emb_int__tok.whitesp_lc_sw.none_cf.def_ner.none_Avg	0.577	0.632	0.603	0.672	0.650	0.661	0.607	0.684	0.643	0.636
CF.bio_emb_int__tok.whitesp_lc_sw.none_cf.def_ner.none_Avg	0.577	0.632	0.603	0.672	0.650	0.661	0.607	0.684	0.643	0.636
TOK.bio_emb_int__tok.whitesp_lc_sw.none_cf.def_ner.none_Avg	0.577	0.632	0.603	0.672	0.650	0.661	0.607	0.684	0.643	0.636
LC.bio_emb_int__tok.whitesp_lc_sw.none_cf.def_ner.none_Avg	0.577	0.632	0.603	0.672	0.650	0.661	0.607	0.684	0.643	0.636
SW.bio_emb_int__tok.whitesp_lc_sw.none_cf.def_ner.none_Sum	0.577	0.632	0.603	0.672	0.650	0.661	0.607	0.684	0.643	0.636
CF.bio_emb_int__tok.whitesp_lc_sw.none_cf.def_ner.none_Sum	0.577	0.632	0.603	0.672	0.650	0.661	0.607	0.684	0.643	0.636
TOK.bio_emb_int__tok.whitesp_lc_sw.none_cf.def_ner.none_Sum	0.577	0.632	0.603	0.672	0.650	0.661	0.607	0.684	0.643	0.636
LC.bio_emb_int__tok.whitesp_lc_sw.none_cf.def_ner.none_Sum	0.577	0.632	0.603	0.672	0.650	0.661	0.607	0.684	0.643	0.636
SW.PubMed.PMC.w2v__tok.whitesp_lc_sw.none_cf.def_ner.none_Max	0.543	0.548	0.545	0.668	0.598	0.631	0.670	0.702	0.686	0.621
CF.PubMed.PMC.w2v__tok.whitesp_lc_sw.none_cf.def_ner.none_Max	0.543	0.548	0.545	0.668	0.598	0.631	0.670	0.702	0.686	0.621
TOK.PubMed.PMC.w2v__tok.whitesp_lc_sw.none_cf.def_ner.none_Max	0.543	0.548	0.545	0.668	0.598	0.631	0.670	0.702	0.686	0.621
LC.PubMed.PMC.w2v__tok.whitesp_lc_sw.none_cf.def_ner.none_Max	0.543	0.548	0.545	0.668	0.598	0.631	0.670	0.702	0.686	0.621
TOK.bio_emb_int__tok.bioc_lc_sw.none_cf.def_ner.none_Avg	0.584	0.635	0.608	0.575	0.636	0.604	0.607	0.684	0.643	0.619
TOK.bio_emb_int__tok.bioc_lc_sw.none_cf.def_ner.none_Sum	0.584	0.635	0.608	0.575	0.636	0.604	0.607	0.684	0.643	0.619
CF.PubMed.PMC.w2v__tok.whitesp_lc_sw.none_cf.none_ner.none_Avg	0.585	0.575	0.580	0.640	0.574	0.605	0.633	0.712	0.670	0.618
CF.PubMed.PMC.w2v__tok.whitesp_lc_sw.none_cf.none_ner.none_Sum	0.585	0.575	0.580	0.640	0.574	0.605	0.633	0.712	0.670	0.618
CF.PubMed.PMC.w2v__tok.whitesp_lc_sw.none_cf.blagec_ner.none_Max	0.526	0.520	0.523	0.674	0.611	0.641	0.662	0.707	0.684	0.616
LC.bio_emb_int__tok.whitesp_notlc_sw.none_cf.def_ner.none_Min	0.570	0.571	0.571	0.652	0.649	0.650	0.586	0.665	0.623	0.615
CF.PubMed.PMC.w2v__tok.whitesp_lc_sw.none_cf.biosses_ner.none_Max	0.527	0.524	0.526	0.672	0.606	0.638	0.661	0.701	0.680	0.614
TOK.PubMed.PMC.w2v__tok.bioc_lc_sw.none_cf.def_ner.none_Max	0.533	0.541	0.537	0.617	0.587	0.601	0.670	0.702	0.686	0.608
CF.PubMed.PMC.w2v__tok.whitesp_lc_sw.none_cf.blagec_ner.none_Min	0.517	0.526	0.522	0.671	0.602	0.635	0.649	0.677	0.663	0.606
SW.PubMed.PMC.w2v__tok.whitesp_lc_sw.none_cf.def_ner.none_Min	0.512	0.545	0.528	0.663	0.587	0.622	0.654	0.676	0.665	0.605
CF.PubMed.PMC.w2v__tok.whitesp_lc_sw.none_cf.def_ner.none_Min	0.512	0.545	0.528	0.663	0.587	0.622	0.654	0.676	0.665	0.605
TOK.PubMed.PMC.w2v__tok.whitesp_lc_sw.none_cf.def_ner.none_Min	0.512	0.545	0.528	0.663	0.587	0.622	0.654	0.676	0.665	0.605
LC.PubMed.PMC.w2v__tok.whitesp_lc_sw.none_cf.def_ner.none_Min	0.512	0.545	0.528	0.663	0.587	0.622	0.654	0.676	0.665	0.605
LC.PubMed.PMC.w2v__tok.whitesp_notlc_sw.none_cf.def_ner.none_Max	0.589	0.589	0.589	0.573	0.511	0.540	0.674	0.699	0.686	0.605
LC.bio_emb_int__tok.whitesp_notlc_sw.none_cf.def_ner.none_Max	0.516	0.515	0.515	0.642	0.625	0.633	0.626	0.691	0.657	0.602
CF.PubMed.PMC.w2v__tok.whitesp_lc_sw.none_cf.biosses_ner.none_Min	0.509	0.522	0.515	0.666	0.595	0.628	0.650	0.672	0.661	0.602
CF.bio_emb_int__tok.whitesp_lc_sw.none_cf.none_ner.none_Avg	0.579	0.611	0.595	0.514	0.584	0.547	0.616	0.689	0.651	0.597
CF.bio_emb_int__tok.whitesp_lc_sw.none_cf.none_ner.none_Sum	0.579	0.611	0.595	0.514	0.584	0.547	0.616	0.689	0.651	0.597
TOK.BioNLP.win.2__tok.corenlp_lc_sw.none_cf.def_ner.none_Min	0.482	0.515	0.498	0.728	0.637	0.679	0.583	0.649	0.614	0.597
SW.PubMed.PMC.w2v__tok.whitesp_lc_sw.nltk_cf.def_ner.none_Max	0.494	0.503	0.498	0.674	0.594	0.631	0.654	0.667	0.660	0.597
SW.PubMed.PMC.w2v__tok.whitesp_lc_sw.biosses_cf.def_ner.none_Max	0.492	0.505	0.499	0.675	0.595	0.633	0.649	0.665	0.657	0.596
LC.PubMed.PMC.w2v__tok.whitesp_notlc_sw.none_cf.def_ner.none_Min	0.558	0.608	0.582	0.579	0.516	0.546	0.638	0.662	0.650	0.593
TOK.BioNLP.win.2__tok.corenlp_lc_sw.none_cf.def_ner.none_Avg	0.490	0.552	0.519	0.697	0.621	0.657	0.554	0.647	0.597	0.591
TOK.BioNLP.win.2__tok.corenlp_lc_sw.none_cf.def_ner.none_Sum	0.490	0.552	0.519	0.697	0.621	0.657	0.554	0.647	0.597	0.591
LC.BioNLP.win.2__tok.whitesp_notlc_sw.none_cf.def_ner.none_Avg	0.518	0.590	0.552	0.587	0.590	0.589	0.571	0.698	0.628	0.589
LC.BioNLP.win.2__tok.whitesp_notlc_sw.none_cf.def_ner.none_Sum	0.518	0.590	0.552	0.587	0.590	0.589	0.571	0.698	0.628	0.589
TOK.PubMed.PMC.w2v__tok.bioc_lc_sw.none_cf.def_ner.none_Min	0.503	0.535	0.519	0.595	0.574	0.585	0.654	0.676	0.665	0.589
TOK.BioNLP.win.2__tok.corenlp_lc_sw.none_cf.def_ner.none_Max	0.498	0.534	0.515	0.722	0.629	0.672	0.542	0.612	0.575	0.587
SW.PubMed.PMC.w2v__tok.whitesp_lc_sw.nltk_cf.def_ner.none_Min	0.455	0.500	0.477	0.674	0.591	0.630	0.639	0.655	0.647	0.585
SW.PubMed.PMC.w2v__tok.whitesp_lc_sw.biosses_cf.def_ner.none_Min	0.450	0.495	0.471	0.674	0.592	0.630	0.639	0.659	0.649	0.583

Table A.10: Part 2 of 2: Pearson (r), Spearman (*ho*) and Harmonic score (h) obtained by the SWEM similarity methods evaluated herein.

	BIOSSES			MedSTS			CTR			Avg
	r	ρ	h	r	ρ	h	r	ρ	h	Avg
SW.BioNLP.win.2__tok.whitesp_lc_sw.none_cf.def_ner.none_Avg	0.443	0.513	0.476	0.685	0.622	0.652	0.548	0.659	0.598	0.575
CF.BioNLP.win.2__tok.whitesp_lc_sw.none_cf.def_ner.none_Avg	0.443	0.513	0.476	0.685	0.622	0.652	0.548	0.659	0.598	0.575
TOK.BioNLP.win.2__tok.whitesp_lc_sw.none_cf.def_ner.none_Avg	0.443	0.513	0.476	0.685	0.622	0.652	0.548	0.659	0.598	0.575
LC.BioNLP.win.2__tok.whitesp_lc_sw.none_cf.def_ner.none_Avg	0.443	0.513	0.476	0.685	0.622	0.652	0.548	0.659	0.598	0.575
SW.BioNLP.win.2__tok.whitesp_lc_sw.none_cf.def_ner.none_Sum	0.443	0.513	0.476	0.685	0.622	0.652	0.548	0.659	0.598	0.575
CF.BioNLP.win.2__tok.whitesp_lc_sw.none_cf.def_ner.none_Sum	0.443	0.513	0.476	0.685	0.622	0.652	0.548	0.659	0.598	0.575
TOK.BioNLP.win.2__tok.whitesp_lc_sw.none_cf.def_ner.none_Sum	0.443	0.513	0.476	0.685	0.622	0.652	0.548	0.659	0.598	0.575
LC.BioNLP.win.2__tok.whitesp_lc_sw.none_cf.def_ner.none_Sum	0.443	0.513	0.476	0.685	0.622	0.652	0.548	0.659	0.598	0.575
SW.BioNLP.win.2__tok.whitesp_lc_sw.none_cf.def_ner.none_Max	0.440	0.499	0.468	0.716	0.632	0.671	0.548	0.614	0.579	0.572
CF.BioNLP.win.2__tok.whitesp_lc_sw.none_cf.def_ner.none_Max	0.440	0.499	0.468	0.716	0.632	0.671	0.548	0.614	0.579	0.572
TOK.BioNLP.win.2__tok.whitesp_lc_sw.none_cf.def_ner.none_Max	0.440	0.499	0.468	0.716	0.632	0.671	0.548	0.614	0.579	0.572
LC.BioNLP.win.2__tok.whitesp_lc_sw.none_cf.def_ner.none_Max	0.440	0.499	0.468	0.716	0.632	0.671	0.548	0.614	0.579	0.572
CF.BioNLP.win.2__tok.whitesp_lc_sw.none_cf.biosses_ner.none_Avg	0.434	0.504	0.466	0.687	0.624	0.654	0.543	0.658	0.595	0.572
CF.BioNLP.win.2__tok.whitesp_lc_sw.none_cf.biosses_ner.none_Sum	0.434	0.504	0.466	0.687	0.624	0.654	0.543	0.658	0.595	0.572
SW.BioNLP.win.2__tok.whitesp_lc_sw.none_cf.def_ner.none_Min	0.400	0.425	0.412	0.723	0.642	0.680	0.589	0.644	0.615	0.569
CF.BioNLP.win.2__tok.whitesp_lc_sw.none_cf.def_ner.none_Min	0.400	0.425	0.412	0.723	0.642	0.680	0.589	0.644	0.615	0.569
TOK.BioNLP.win.2__tok.whitesp_lc_sw.none_cf.def_ner.none_Min	0.400	0.425	0.412	0.723	0.642	0.680	0.589	0.644	0.615	0.569
LC.BioNLP.win.2__tok.whitesp_lc_sw.none_cf.def_ner.none_Min	0.400	0.425	0.412	0.723	0.642	0.680	0.589	0.644	0.615	0.569
CF.BioNLP.win.2__tok.whitesp_lc_sw.none_cf.biosses_ner.none_Max	0.425	0.492	0.456	0.716	0.633	0.672	0.540	0.606	0.571	0.566
CF.BioNLP.win.2__tok.whitesp_lc_sw.none_cf.blagec_ner.none_Max	0.427	0.490	0.457	0.715	0.635	0.673	0.531	0.605	0.566	0.565
CF.BioNLP.win.2__tok.whitesp_lc_sw.none_cf.biosses_ner.none_Min	0.381	0.418	0.399	0.722	0.643	0.680	0.580	0.636	0.606	0.562
TOK.BioNLP.win.2__tok.bioc_lc_sw.none_cf.def_ner.none_Avg	0.448	0.517	0.480	0.590	0.607	0.598	0.548	0.659	0.598	0.559
TOK.BioNLP.win.2__tok.bioc_lc_sw.none_cf.def_ner.none_Sum	0.448	0.517	0.480	0.590	0.607	0.598	0.548	0.659	0.598	0.559
CF.PubMed.PMC.w2v__tok.whitesp_lc_sw.none_cf.none_ner.none_Max	0.407	0.423	0.415	0.591	0.544	0.567	0.681	0.709	0.694	0.559
SW.BioNLP.win.2__tok.whitesp_lc_sw.nltk_cf.def_ner.none_Max	0.384	0.453	0.415	0.716	0.630	0.670	0.576	0.599	0.587	0.558
CF.BioNLP.win.2__tok.whitesp_lc_sw.none_cf.blagec_ner.none_Min	0.375	0.413	0.393	0.721	0.644	0.680	0.568	0.634	0.599	0.557
SW.BioNLP.win.2__tok.whitesp_lc_sw.nltk_cf.def_ner.none_Min	0.361	0.396	0.378	0.726	0.644	0.683	0.604	0.619	0.611	0.557
CF.BioNLP.win.2__tok.whitesp_lc_sw.none_cf.blagec_ner.none_Avg	0.432	0.490	0.459	0.682	0.625	0.652	0.502	0.626	0.557	0.556
CF.BioNLP.win.2__tok.whitesp_lc_sw.none_cf.blagec_ner.none_Sum	0.432	0.490	0.459	0.682	0.625	0.652	0.502	0.626	0.557	0.556
TOK.BioNLP.win.2__tok.bioc_lc_sw.none_cf.def_ner.none_Max	0.444	0.502	0.471	0.620	0.616	0.618	0.548	0.614	0.579	0.556
SW.BioNLP.win.2__tok.whitesp_lc_sw.biosses_cf.def_ner.none_Min	0.356	0.395	0.375	0.727	0.645	0.683	0.595	0.611	0.603	0.554
TOK.BioNLP.win.2__tok.bioc_lc_sw.none_cf.def_ner.none_Min	0.408	0.433	0.420	0.618	0.627	0.622	0.589	0.644	0.615	0.552
SW.BioNLP.win.2__tok.whitesp_lc_sw.biosses_cf.def_ner.none_Max	0.374	0.441	0.405	0.718	0.630	0.671	0.562	0.586	0.574	0.550
CF.PubMed.PMC.w2v__tok.whitesp_lc_sw.none_cf.none_ner.none_Min	0.354	0.401	0.376	0.598	0.542	0.569	0.682	0.704	0.693	0.546
LC.BioNLP.win.2__tok.whitesp_notlc_sw.none_cf.def_ner.none_Min	0.419	0.448	0.433	0.641	0.579	0.608	0.552	0.648	0.596	0.546
LC.bio_emb_int__tok.whitesp_notlc_sw.none_cf.def_ner.none_Avg	0.436	0.497	0.465	0.532	0.619	0.572	0.529	0.674	0.593	0.543
LC.bio_emb_int__tok.whitesp_notlc_sw.none_cf.def_ner.none_Sum	0.436	0.497	0.465	0.532	0.619	0.572	0.529	0.674	0.593	0.543
CF.BioNLP.win.2__tok.whitesp_lc_sw.none_cf.none_ner.none_Avg	0.402	0.486	0.440	0.545	0.568	0.556	0.521	0.622	0.567	0.521
CF.BioNLP.win.2__tok.whitesp_lc_sw.none_cf.none_ner.none_Sum	0.402	0.486	0.440	0.545	0.568	0.556	0.521	0.622	0.567	0.521
CF.BioNLP.win.2__tok.whitesp_lc_sw.none_cf.none_ner.none_Max	0.325	0.372	0.347	0.607	0.584	0.596	0.579	0.631	0.604	0.516
CF.BioNLP.win.2__tok.whitesp_lc_sw.none_cf.none_ner.none_Min	0.272	0.283	0.278	0.621	0.591	0.605	0.606	0.657	0.631	0.505
TOK.bioconc_fastt__tok.corenlp_lc_sw.none_cf.def_ner.none_Min	0.274	0.419	0.331	0.199	0.360	0.256	0.109	0.126	0.117	0.235
TOK.bioconc_fastt__tok.corenlp_lc_sw.none_cf.def_ner.none_Max	0.239	0.365	0.288	0.171	0.331	0.225	0.161	0.162	0.162	0.225
SW.bioconc_fastt__tok.whitesp_lc_sw.biosses_cf.def_ner.none_Avg	0.048	0.124	0.069	0.360	0.437	0.395	0.140	0.276	0.186	0.217
SW.bioconc_fastt__tok.whitesp_lc_sw.biosses_cf.def_ner.none_Sum	0.048	0.124	0.069	0.360	0.437	0.395	0.140	0.276	0.186	0.217
SW.bioconc_fastt__tok.whitesp_lc_sw.nltk_cf.def_ner.none_Avg	0.049	0.126	0.070	0.354	0.435	0.391	0.137	0.267	0.181	0.214
SW.bioconc_fastt__tok.whitesp_lc_sw.nltk_cf.def_ner.none_Sum	0.049	0.126	0.070	0.354	0.435	0.391	0.137	0.267	0.181	0.214
TOK.bioconc_fastt__tok.corenlp_lc_sw.none_cf.def_ner.none_Avg	0.020	0.132	0.035	0.294	0.385	0.334	0.225	0.284	0.252	0.207
TOK.bioconc_fastt__tok.corenlp_lc_sw.none_cf.def_ner.none_Sum	0.020	0.132	0.035	0.294	0.385	0.334	0.225	0.284	0.252	0.207
SW.bioconc_fastt__tok.whitesp_lc_sw.nltk_cf.def_ner.none_Min	0.164	0.338	0.220	0.167	0.335	0.223	0.141	0.202	0.166	0.203
SW.bioconc_fastt__tok.whitesp_lc_sw.biosses_cf.def_ner.none_Min	0.164	0.335	0.220	0.162	0.334	0.218	0.146	0.192	0.166	0.201
SW.bioconc_fastt__tok.whitesp_lc_sw.none_cf.def_ner.none_Min	0.187	0.380	0.251	0.159	0.324	0.213	0.138	0.137	0.138	0.200
CF.bioconc_fastt__tok.whitesp_lc_sw.none_cf.def_ner.none_Min	0.187	0.380	0.251	0.159	0.324	0.213	0.138	0.137	0.138	0.200
TOK.bioconc_fastt__tok.whitesp_lc_sw.none_cf.def_ner.none_Min	0.187	0.380	0.251	0.159	0.324	0.213	0.138	0.137	0.138	0.200
LC.bioconc_fastt__tok.whitesp_lc_sw.none_cf.def_ner.none_Min	0.187	0.380	0.251	0.159	0.324	0.213	0.138	0.137	0.138	0.200
TOK.bioconc_fastt__tok.bioc_lc_sw.none_cf.def_ner.none_Min	0.176	0.363	0.237	0.166	0.309	0.216	0.138	0.137	0.138	0.197
CF.bioconc_fastt__tok.whitesp_lc_sw.none_cf.none_ner.none_Min	0.152	0.306	0.203	0.168	0.315	0.219	0.137	0.182	0.157	0.193
SW.bioconc_fastt__tok.whitesp_lc_sw.none_cf.def_ner.none_Avg	0.011	0.079	0.020	0.260	0.381	0.309	0.201	0.261	0.227	0.185
CF.bioconc_fastt__tok.whitesp_lc_sw.none_cf.def_ner.none_Avg	0.011	0.079	0.020	0.260	0.381	0.309	0.201	0.261	0.227	0.185
TOK.bioconc_fastt__tok.whitesp_lc_sw.none_cf.def_ner.none_Avg	0.011	0.079	0.020	0.260	0.381	0.309	0.201	0.261	0.227	0.185
LC.bioconc_fastt__tok.whitesp_lc_sw.none_cf.def_ner.none_Avg	0.011	0.079	0.020	0.260	0.381	0.309	0.201	0.261	0.227	0.185
SW.bioconc_fastt__tok.whitesp_lc_sw.none_cf.def_ner.none_Sum	0.011	0.079	0.020	0.260	0.381	0.309	0.201	0.261	0.227	0.185
CF.bioconc_fastt__tok.whitesp_lc_sw.none_cf.def_ner.none_Sum	0.011	0.079	0.020	0.260	0.381	0.309	0.201	0.261	0.227	0.185
TOK.bioconc_fastt__tok.whitesp_lc_sw.none_cf.def_ner.none_Sum	0.011	0.079	0.020	0.260	0.381	0.309	0.201	0.261	0.227	0.185
LC.bioconc_fastt__tok.whitesp_lc_sw.none_cf.def_ner.none_Sum	0.011	0.079	0.020	0.260	0.381	0.309	0.201	0.261	0.227	0.185
CF.bioconc_fastt__tok.whitesp_lc_sw.none_cf.none_ner.none_Max	0.088	0.192	0.120	0.154	0.283	0.199	0.230	0.229	0.230	0.183
SW.bioconc_fastt__tok.whitesp_lc_sw.none_cf.def_ner.none_Max	0.128	0.278	0.175	0.142	0.296	0.192	0.183	0.159	0.171	0.179
CF.bioconc_fastt__tok.whitesp_lc_sw.none_cf.def_ner.none_Max	0.128	0.278	0.175	0.142	0.296	0.192	0.183	0.159	0.171	0.179
TOK.bioconc_fastt__tok.whitesp_lc_sw.none_cf.def_ner.none_Max	0.128	0.278	0.175	0.142	0.296	0.192	0.183	0.159	0.171	0.179
LC.bioconc_fastt__tok.whitesp_lc_sw.none_cf.def_ner.none_Max	0.128	0.278	0.175	0.142	0.296	0.192	0.183	0.159	0.171	0.179
LC.bioconc_fastt__tok.whitesp_notlc_sw.none_cf.def_ner.none_Avg	0.005	0.031	0.009	0.225	0.397	0.287	0.213	0.270	0.238	0.178
LC.bioconc_fastt__tok.whitesp_notlc_sw.none_cf.def_ner.none_Sum	0.005	0.031	0.009	0.225	0.397	0.287	0.213	0.270	0.238	0.178
CF.bioconc_fastt__tok.whitesp_lc_sw.none_cf.blagec_ner.none_Avg	0.013	0.071	0.022	0.238	0.374	0.291	0.198	0.240	0.217	0.177
CF.bioconc_fastt__tok.whitesp_lc_sw.none_cf.blagec_ner.none_Sum	0.013	0.071	0.022	0.238	0.374	0.291	0.198	0.240	0.217	0.177
TOK.bioconc_fastt__tok.bioc_lc_sw.none_cf.def_ner.none_Max	0.126	0.279	0.174	0.135	0.281	0.183	0.183	0.159	0.171	0.176
SW.bioconc_fastt__tok.whitesp_lc_sw.nltk_cf.def_ner.none_Max	0.077	0.250	0.118	0.147	0.307	0.199	0.186	0.215	0.199	0.172
SW.bioconc_fastt__tok.whitesp_lc_sw.biosses_cf.def_ner.none_Max	0.074	0.245	0.113	0.134	0.306	0.186	0.183	0.213	0.197	0.165
CF.bioconc_fastt__tok.whitesp_lc_sw.none_cf.none_ner.none_Avg	0.031	0.096	0.047	0.145	0.343	0.204	0.217	0.267	0.239	0.163
CF.bioconc_fastt__tok.whitesp_lc_sw.none_cf.none_ner.none_Sum	0.031	0.096	0.047	0.145	0.343	0.204	0.217	0.267	0.239	0.163
CF.bioconc_fastt__tok.whitesp_lc_sw.none_cf.blagec_ner.none_Min	0.142	0.296	0.192	0.144	0.318	0.198	0.107	0.091	0.098	0.163
TOK.bioconc_fastt__tok.bioc_lc_sw.none_cf.def_ner.none_Avg	0.019	0.090	0.031	0.165	0.369	0.228	0.201	0.261	0.227	0.162
TOK.bioconc_fastt__tok.bioc_lc_sw.none_cf.def_ner.none_Sum	0.019	0.090	0.031	0.165	0.369	0.228	0.201	0.261	0.227	0.162
CF.bioconc_fastt__tok.whitesp_lc_sw.none_cf.biosses_ner.none_Min	0.127	0.317	0.181	0.147	0.316	0.201	0.107	0.091	0.099	0.160
CF.bioconc_fastt__tok.whitesp_lc_sw.none_cf.biosses_ner.none_Max	0.096	0.240	0.137	0.128	0.285	0.177	0.190	0.147	0.166	0.160
CF.bioconc_fastt__tok.whitesp_lc_sw.none_cf.blagec_ner.none_Max	0.096	0.223	0.134	0.125	0.285	0.174	0.190	0.148	0.167	0.158
CF.bioconc_fastt__tok.whitesp_lc_sw.none_cf.biosses_ner.none_Avg	-0.026	0.077	-0.078	0.246	0.381	0.299	0.198	0.239	0.217	0.146
CF.bioconc_fastt__tok.whitesp_lc_sw.none_cf.biosses_ner.none_Sum	-0.026	0.077	-0.078	0.246	0.381	0.299	0.198	0.239	0.217	0.146
LC.bioconc_fastt__tok.whitesp_notlc_sw.none_cf.def_ner.none_Max	0.063	0.214	0.098	0.256	0.293	0.273	0.046	0.016	0.024	0.132
LC.bioconc_fastt__tok.whitesp_notlc_sw.none_cf.def_ner.none_Min	0.042	0.191	0.069	0.274	0.330	0.299	0.004	0.020	0.006	0.125

Table A.11: Pearson (r), Spearman (*ho*) and Harmonic score (h) obtained by the Sent2Vec similarity methods evaluated herein.

	BIOSSES			MedSTS			CTR			Avg
	r	ρ	h	r	ρ	h	r	ρ	h	Avg
SW.Sent2vec_tok.whitesp_lc_sw.biosses_cf.def_ner.none	0.789	0.760	0.774	0.765	0.638	0.696	0.788	0.818	0.803	0.758
SW.Sent2vec_tok.whitesp_lc_sw.nltk_cf.def_ner.none	0.787	0.758	0.772	0.763	0.637	0.694	0.787	0.815	0.801	0.756
TOK.Sent2vec_tok.corenlp_lc_sw.none_cf.def_ner.none	0.809	0.787	0.798	0.739	0.616	0.672	0.771	0.807	0.789	0.753
CF.Sent2vec_tok.whitesp_lc_sw.none_cf.biosses_ner.none	0.791	0.773	0.782	0.738	0.626	0.677	0.782	0.818	0.799	0.753
SW.Sent2vec_tok.whitesp_lc_sw.none_cf.def_ner.none	0.807	0.787	0.797	0.739	0.616	0.672	0.771	0.807	0.789	0.752
CF.Sent2vec_tok.whitesp_lc_sw.none_cf.def_ner.none	0.807	0.787	0.797	0.739	0.616	0.672	0.771	0.807	0.789	0.752
TOK.Sent2vec_tok.whitesp_lc_sw.none_cf.def_ner.none	0.807	0.787	0.797	0.739	0.616	0.672	0.771	0.807	0.789	0.752
LC.Sent2vec_tok.whitesp_lc_sw.none_cf.def_ner.none	0.807	0.787	0.797	0.739	0.616	0.672	0.771	0.807	0.789	0.752
TOK.Sent2vec_tok.bioc_lc_sw.none_cf.def_ner.none	0.809	0.787	0.798	0.722	0.601	0.656	0.771	0.807	0.789	0.747
CF.Sent2vec_tok.whitesp_lc_sw.none_cf.blagec_ner.none	0.770	0.745	0.757	0.738	0.628	0.679	0.782	0.815	0.798	0.745
CF.Sent2vec_tok.whitesp_lc_sw.none_cf.none_ner.none	0.750	0.733	0.741	0.662	0.562	0.608	0.796	0.828	0.812	0.720
LC.Sent2vec_tok.whitesp_notlc_sw.none_cf.def_ner.none	0.681	0.665	0.673	0.697	0.601	0.645	0.697	0.747	0.721	0.680

Table A.12: Pearson (r), Spearman (*ho*) and Harmonic score (h) obtained by the USE similarity methods evaluated herein.

	BIOSSES			MedSTS			CTR			Avg
	r	ρ	h	r	ρ	h	r	ρ	h	Avg
TOK.USE_tok.corenlp_lc_sw.none_cf.def_ner.none	0.666	0.669	0.668	0.679	0.606	0.640	0.663	0.684	0.674	0.660
LC.USE_tok.whitesp_notlc_sw.none_cf.def_ner.none	0.663	0.663	0.663	0.679	0.606	0.640	0.663	0.684	0.674	0.659
LC.USE_tok.whitesp_lc_sw.none_cf.def_ner.none	0.663	0.663	0.663	0.679	0.606	0.640	0.663	0.684	0.674	0.659
TOK.USE_tok.whitesp_lc_sw.none_cf.def_ner.none	0.663	0.663	0.663	0.679	0.606	0.640	0.663	0.684	0.674	0.659
SW.USE_tok.whitesp_lc_sw.none_cf.def_ner.none	0.663	0.663	0.663	0.679	0.606	0.640	0.663	0.684	0.674	0.659
CF.USE_tok.whitesp_lc_sw.none_cf.def_ner.none	0.663	0.663	0.663	0.679	0.606	0.640	0.663	0.684	0.674	0.659
TOK.USE_tok.bioc_lc_sw.none_cf.def_ner.none	0.664	0.668	0.666	0.662	0.591	0.625	0.663	0.684	0.674	0.655
CF.USE_tok.whitesp_lc_sw.none_cf.blagec_ner.none	0.658	0.645	0.651	0.675	0.608	0.640	0.655	0.674	0.664	0.652
CF.USE_tok.whitesp_lc_sw.none_cf.biosses_ner.none	0.658	0.645	0.651	0.675	0.608	0.640	0.655	0.674	0.664	0.652
CF.USE_tok.whitesp_lc_sw.none_cf.none_ner.none	0.658	0.645	0.651	0.675	0.608	0.640	0.655	0.674	0.664	0.652
SW.USE_tok.whitesp_lc_sw.biosses_cf.def_ner.none	0.598	0.598	0.598	0.696	0.624	0.658	0.684	0.715	0.699	0.652
SW.USE_tok.whitesp_lc_sw.nltk_cf.def_ner.none	0.597	0.594	0.596	0.695	0.624	0.658	0.684	0.718	0.701	0.651

Table A.13: Pearson (r), Spearman (*ho*) and Harmonic score (h) obtained by the Flair similarity methods evaluated herein.

	BIOSSES			MedSTS			CTR			Avg
	r	ρ	h	r	ρ	h	r	ρ	h	Avg
LC.Flair_tok.whitesp_notlc_sw.none_cf.def_ner.none	0.578	0.609	0.593	-0.015	-0.035	-0.021	0.653	0.719	0.684	0.419
CF.Flair_tok.whitesp_lc_sw.none_cf.biosses_ner.none	0.603	0.600	0.602	-0.014	-0.035	-0.020	0.642	0.703	0.671	0.418
CF.Flair_tok.whitesp_lc_sw.none_cf.none_ner.none	0.602	0.610	0.606	-0.014	-0.035	-0.020	0.606	0.658	0.631	0.406
SW.Flair_tok.whitesp_lc_sw.none_cf.def_ner.none	0.552	0.606	0.578	-0.015	-0.035	-0.021	0.617	0.684	0.649	0.402
CF.Flair_tok.whitesp_lc_sw.none_cf.def_ner.none	0.552	0.606	0.578	-0.015	-0.035	-0.021	0.617	0.684	0.649	0.402
TOK.Flair_tok.whitesp_lc_sw.none_cf.def_ner.none	0.552	0.606	0.578	-0.015	-0.035	-0.021	0.617	0.684	0.649	0.402
LC.Flair_tok.whitesp_lc_sw.none_cf.def_ner.none	0.552	0.606	0.578	-0.015	-0.035	-0.021	0.617	0.684	0.649	0.402
CF.Flair_tok.whitesp_lc_sw.none_cf.blagec_ner.none	0.575	0.582	0.578	-0.014	-0.035	-0.020	0.614	0.677	0.644	0.401
TOK.Flair_tok.corenlp_lc_sw.none_cf.def_ner.none	0.543	0.607	0.573	-0.015	-0.035	-0.021	0.617	0.684	0.649	0.400
TOK.Flair_tok.bioc_lc_sw.none_cf.def_ner.none	0.543	0.607	0.573	-0.015	-0.036	-0.021	0.617	0.684	0.649	0.400
SW.Flair_tok.whitesp_lc_sw.biosses_cf.def_ner.none	0.479	0.553	0.513	-0.012	-0.035	-0.017	0.626	0.684	0.654	0.383
SW.Flair_tok.whitesp_lc_sw.nltk_cf.def_ner.none	0.474	0.555	0.512	-0.012	-0.035	-0.018	0.621	0.681	0.650	0.381

Table A.14: Part 1 of 2: Pearson (r), Spearman (*ho*) and Harmonic score (h) obtained by the BERT similarity methods evaluated herein.

	BIOSSES			MedSTS			CTR			Avg
	r	ρ	h	r	ρ	h	r	ρ	h	Avg
SW.oubiob.base.uncased_tok.wordpie_lc_sw.none_cf.def_ner.none	0.687	0.729	0.707	0.707	0.583	0.639	0.670	0.695	0.682	0.676
CF.ncbi_pubmed_l.12_tok.wordp_lc_sw.none_cf.biosses_ner.none	0.616	0.630	0.623	0.672	0.559	0.610	0.651	0.695	0.673	0.635
CF.ncbi_pubmed_l.12_tok.wordp_lc_sw.none_cf.blagec_ner.none	0.595	0.604	0.599	0.675	0.564	0.615	0.655	0.693	0.673	0.629
CF.biob_large_v1.1_pubmed_tok.wordp_lc_sw.none_cf.biosses_ner.none	0.614	0.603	0.608	0.719	0.626	0.670	0.573	0.594	0.583	0.620
CF.biob_large_v1.1_pubmed_tok.wordp_lc_sw.none_cf.blagec_ner.none	0.609	0.605	0.607	0.714	0.624	0.666	0.565	0.577	0.571	0.615
CF.ncbi_pubmed_l.12_tok.wordp_lc_sw.none_cf.none_ner.none	0.548	0.572	0.560	0.658	0.534	0.590	0.667	0.711	0.688	0.613
SW.scibert_scivocab_tok.wordp_lc_sw.nltk_cf.def_ner.none	0.582	0.565	0.574	0.708	0.637	0.671	0.549	0.623	0.584	0.609
SW.oubiob.base.uncased_tok.wordp_lc_sw.nltk_cf.def_ner.none	0.625	0.639	0.632	0.725	0.623	0.670	0.676	0.708	0.692	0.665
CF.oubiob.base.uncased_tok.wordp_lc_sw.none_cf.def_ner.none	0.548	0.562	0.555	0.715	0.600	0.652	0.599	0.643	0.621	0.609
LC.oubiob.base.uncased_tok.wordp_lc_sw.none_cf.def_ner.none	0.548	0.562	0.555	0.715	0.600	0.652	0.599	0.643	0.621	0.609
LC.oubiob.base.uncased_tok.wordp_notlc_sw.none_cf.def_ner.none	0.548	0.562	0.555	0.715	0.600	0.652	0.599	0.643	0.621	0.609
SW.PubMedBERT.abs+full_tok.wordp_lc_sw.biosses_cf.def_ner.none	0.584	0.539	0.561	0.716	0.615	0.662	0.579	0.596	0.587	0.603
SW.scibert_scivocab_tok.wordp_lc_sw.biosses_cf.def_ner.none	0.532	0.523	0.527	0.706	0.640	0.671	0.567	0.649	0.605	0.601
SW.PubMedBERT.abs+full_tok.wordp_lc_sw.nltk_cf.def_ner.none	0.589	0.549	0.569	0.716	0.613	0.660	0.552	0.584	0.568	0.599
SW.biob_v1.0_pmc_tok.wordp_lc_sw.none_cf.def_ner.none	0.635	0.599	0.616	0.643	0.570	0.604	0.558	0.574	0.566	0.595
CF.biob_v1.0_pmc_tok.wordp_lc_sw.none_cf.def_ner.none	0.635	0.599	0.616	0.643	0.570	0.604	0.558	0.574	0.566	0.595
LC.biob_v1.0_pmc_tok.wordp_lc_sw.none_cf.def_ner.none	0.635	0.599	0.616	0.643	0.570	0.604	0.558	0.574	0.566	0.595
LC.biob_v1.0_pmc_tok.wordp_notlc_sw.none_cf.def_ner.none	0.635	0.599	0.616	0.643	0.570	0.604	0.558	0.574	0.566	0.595
SW.oubiob.base.uncased_tok.wordp_lc_sw.biosses_cf.def_ner.none	0.608	0.627	0.617	0.730	0.622	0.672	0.669	0.696	0.682	0.657
CF.scibert_scivocab_tok.wordp_lc_sw.none_cf.biosses_ner.none	0.624	0.635	0.629	0.610	0.561	0.585	0.543	0.603	0.571	0.595
CF.biob_v1.0_pmc_tok.wordp_lc_sw.none_cf.blagec_ner.none	0.596	0.567	0.581	0.654	0.566	0.607	0.577	0.597	0.587	0.592
CF.biob_v1.1_pubmed_tok.wordp_lc_sw.none_cf.biosses_ner.none	0.553	0.545	0.549	0.673	0.576	0.621	0.586	0.620	0.602	0.591
CF.biob_v1.0_pmc_tok.wordp_lc_sw.none_cf.biosses_ner.none	0.579	0.543	0.560	0.656	0.571	0.610	0.587	0.613	0.600	0.590
SW.PubMedBERT.abs+full_tok.wordp_lc_sw.none_cf.def_ner.none	0.502	0.530	0.515	0.711	0.594	0.647	0.580	0.635	0.606	0.590
CF.PubMedBERT.abs+full_tok.wordp_lc_sw.none_cf.def_ner.none	0.502	0.530	0.515	0.711	0.594	0.647	0.580	0.635	0.606	0.590
LC.PubMedBERT.abs+full_tok.wordp_lc_sw.none_cf.def_ner.none	0.502	0.530	0.515	0.711	0.594	0.647	0.580	0.635	0.606	0.590
LC.PubMedBERT.abs+full_tok.wordp_notlc_sw.none_cf.def_ner.none	0.502	0.530	0.515	0.711	0.594	0.647	0.580	0.635	0.606	0.590
CF.biob_v1.1_pubmed_tok.wordp_lc_sw.none_cf.blagec_ner.none	0.552	0.548	0.550	0.669	0.577	0.619	0.583	0.614	0.598	0.589
CF.ncbi_pubmed_l.24_tok.wordp_lc_sw.none_cf.biosses_ner.none	0.475	0.527	0.500	0.627	0.580	0.603	0.630	0.697	0.662	0.588
CF.ncbi_pubmed_l.24_tok.wordp_lc_sw.none_cf.blagec_ner.none	0.465	0.518	0.490	0.637	0.586	0.610	0.613	0.681	0.645	0.582
CF.biob_large_v1.1_pubmed_tok.wordp_lc_sw.none_cf.none_ner.none	0.527	0.532	0.529	0.686	0.621	0.652	0.553	0.573	0.563	0.581
SW.ncbi_pubmed_l.12_tok.wordp_lc_sw.none_cf.def_ner.none	0.548	0.527	0.538	0.659	0.551	0.600	0.588	0.615	0.601	0.580
CF.ncbi_pubmed_l.12_tok.wordp_lc_sw.none_cf.def_ner.none	0.548	0.527	0.538	0.659	0.551	0.600	0.588	0.615	0.601	0.580
LC.ncbi_pubmed_l.12_tok.wordp_lc_sw.none_cf.def_ner.none	0.548	0.527	0.538	0.659	0.551	0.600	0.588	0.615	0.601	0.580
LC.ncbi_pubmed_l.12_tok.wordp_notlc_sw.none_cf.def_ner.none	0.548	0.527	0.538	0.659	0.551	0.600	0.588	0.615	0.601	0.580
SW.scibert_scivocab_tok.wordp_lc_sw.none_cf.def_ner.none	0.603	0.597	0.600	0.597	0.555	0.575	0.531	0.594	0.560	0.579
CF.scibert_scivocab_tok.wordp_lc_sw.none_cf.def_ner.none	0.603	0.597	0.600	0.597	0.555	0.575	0.531	0.594	0.560	0.579
LC.scibert_scivocab_tok.wordp_lc_sw.none_cf.def_ner.none	0.603	0.597	0.600	0.597	0.555	0.575	0.531	0.594	0.560	0.579
LC.scibert_scivocab_tok.wordp_notlc_sw.none_cf.def_ner.none	0.603	0.597	0.600	0.597	0.555	0.575	0.531	0.594	0.560	0.579
CF.biob_v1.0_pubm+pmc_tok.wordp_lc_sw.none_cf.blagec_ner.none	0.538	0.526	0.532	0.636	0.550	0.590	0.595	0.619	0.607	0.576
CF.biob_v1.0_pubm+pmc_tok.wordp_lc_sw.none_cf.biosses_ner.none	0.533	0.511	0.521	0.635	0.552	0.590	0.598	0.627	0.612	0.575
CF.scibert_scivocab_tok.wordp_lc_sw.none_cf.blagec_ner.none	0.609	0.611	0.610	0.598	0.554	0.575	0.506	0.549	0.526	0.571
CF.biob_v1.0_pubmed_tok.wordp_lc_sw.none_cf.biosses_ner.none	0.462	0.475	0.468	0.662	0.576	0.616	0.583	0.627	0.604	0.563
CF.oubiob.base.uncased_tok.wordp_lc_sw.none_cf.none_ner.none	0.495	0.479	0.487	0.679	0.551	0.608	0.585	0.598	0.591	0.562
CF.ncbi_pubmed_mimic_l.24_tok.wordp_lc_sw.none_cf.blagec_ner.none	0.475	0.532	0.502	0.667	0.619	0.642	0.512	0.551	0.531	0.558
SW.biob_v1.1_pubmed_tok.wordp_lc_sw.none_cf.def_ner.none	0.528	0.521	0.524	0.633	0.573	0.601	0.542	0.552	0.547	0.558
CF.biob_v1.1_pubmed_tok.wordp_lc_sw.none_cf.def_ner.none	0.528	0.521	0.524	0.633	0.573	0.601	0.542	0.552	0.547	0.558
LC.biob_v1.1_pubmed_tok.wordp_lc_sw.none_cf.def_ner.none	0.528	0.521	0.524	0.633	0.573	0.601	0.542	0.552	0.547	0.558
LC.biob_v1.1_pubmed_tok.wordp_notlc_sw.none_cf.def_ner.none	0.528	0.521	0.524	0.633	0.573	0.601	0.542	0.552	0.547	0.558
CF.biob_v1.0_pubmed_tok.wordp_lc_sw.none_cf.blagec_ner.none	0.470	0.486	0.478	0.660	0.574	0.614	0.565	0.596	0.580	0.557
CF.ncbi_pubmed_mimic_l.24_tok.wordp_lc_sw.none_cf.biosses_ner.none	0.464	0.516	0.488	0.668	0.620	0.643	0.520	0.562	0.540	0.557
CF.biob_v1.1_pubmed_tok.wordp_lc_sw.none_cf.none_ner.none	0.509	0.498	0.504	0.637	0.548	0.589	0.560	0.578	0.569	0.554
CF.ncbi_pubmed_l.24_tok.wordp_lc_sw.none_cf.none_ner.none	0.444	0.468	0.456	0.599	0.554	0.576	0.613	0.643	0.628	0.553
SW.biob_v1.0_pubm+pmc_tok.wordp_lc_sw.none_cf.def_ner.none	0.586	0.554	0.569	0.615	0.546	0.578	0.509	0.512	0.511	0.553
CF.biob_v1.0_pubm+pmc_tok.wordp_lc_sw.none_cf.def_ner.none	0.586	0.554	0.569	0.615	0.546	0.578	0.509	0.512	0.511	0.553
LC.biob_v1.0_pubm+pmc_tok.wordp_lc_sw.none_cf.def_ner.none	0.586	0.554	0.569	0.615	0.546	0.578	0.509	0.512	0.511	0.553
LC.biob_v1.0_pubm+pmc_tok.wordp_notlc_sw.none_cf.def_ner.none	0.586	0.554	0.569	0.615	0.546	0.578	0.509	0.512	0.511	0.553
CF.ncbi_pubmed_mimic_l.12_tok.wordp_lc_sw.none_cf.biosses_ner.none	0.410	0.423	0.416	0.702	0.596	0.645	0.574	0.615	0.593	0.551
SW.biob_large_v1.1_pubmed_tok.wordp_lc_sw.none_cf.def_ner.none	0.514	0.476	0.494	0.688	0.598	0.640	0.518	0.518	0.518	0.551
CF.biob_large_v1.1_pubmed_tok.wordp_lc_sw.none_cf.def_ner.none	0.514	0.476	0.494	0.688	0.598	0.640	0.518	0.518	0.518	0.551
LC.biob_large_v1.1_pubmed_tok.wordp_lc_sw.none_cf.def_ner.none	0.514	0.476	0.494	0.688	0.598	0.640	0.518	0.518	0.518	0.551
LC.biob_large_v1.1_pubmed_tok.wordp_notlc_sw.none_cf.def_ner.none	0.514	0.476	0.494	0.688	0.598	0.640	0.518	0.518	0.518	0.551
CF.ncbi_pubmed_mimic_l.12_tok.wordp_lc_sw.none_cf.blagec_ner.none	0.428	0.452	0.440	0.705	0.599	0.647	0.547	0.577	0.562	0.550
SW.biob_v1.0_pubmed_tok.wordp_lc_sw.none_cf.def_ner.none	0.509	0.484	0.497	0.628	0.562	0.593	0.546	0.570	0.558	0.549
CF.biob_v1.0_pubmed_tok.wordp_lc_sw.none_cf.def_ner.none	0.509	0.484	0.497	0.628	0.562	0.593	0.546	0.570	0.558	0.549
LC.biob_v1.0_pubmed_tok.wordp_lc_sw.none_cf.def_ner.none	0.509	0.484	0.497	0.628	0.562	0.593	0.546	0.570	0.558	0.549
LC.biob_v1.0_pubmed_tok.wordp_notlc_sw.none_cf.def_ner.none	0.509	0.484	0.497	0.628	0.562	0.593	0.546	0.570	0.558	0.549
CF.oubiob.base.uncased_tok.wordp_lc_sw.none_cf.biosses_ner.none	0.520	0.499	0.509	0.658	0.544	0.595	0.523	0.555	0.538	0.548
CF.oubiob.base.uncased_tok.wordp_lc_sw.none_cf.blagec_ner.none	0.512	0.480	0.495	0.657	0.541	0.593	0.534	0.564	0.549	0.546
CF.biob_v1.0_pubmed_tok.wordp_lc_sw.none_cf.none_ner.none	0.451	0.457	0.454	0.636	0.543	0.586	0.585	0.609	0.597	0.546
SW.ncbi_pubmed_mimic_l.12_tok.wordp_lc_sw.none_cf.def_ner.none	0.459	0.478	0.468	0.688	0.586	0.633	0.518	0.543	0.530	0.544
CF.ncbi_pubmed_mimic_l.12_tok.wordp_lc_sw.none_cf.def_ner.none	0.459	0.478	0.468	0.688	0.586	0.633	0.518	0.543	0.530	0.544
LC.ncbi_pubmed_mimic_l.12_tok.wordp_lc_sw.none_cf.def_ner.none	0.459	0.478	0.468	0.688	0.586	0.633	0.518	0.543	0.530	0.544
LC.ncbi_pubmed_mimic_l.12_tok.wordp_notlc_sw.none_cf.def_ner.none	0.459	0.478	0.468	0.688	0.586	0.633	0.518	0.543	0.530	0.544
CF.biob_v1.0_pmc_tok.wordp_lc_sw.none_cf.none_ner.none	0.498	0.481	0.490	0.610	0.505	0.553	0.575	0.600	0.587	0.543
SW.PubMedBERT.abstract_tok.wordp_lc_sw.biosses_cf.def_ner.none	0.519	0.559	0.538	0.595	0.524	0.557	0.492	0.583	0.534	0.543
SW.PubMedBERT.abstract_tok.wordp_lc_sw.nltk_cf.def_ner.none	0.517	0.556	0.536	0.600	0.526	0.561	0.477	0.580	0.523	0.540
SW.biob_large_v1.1_pubmed_tok.wordp_lc_sw.nltk_cf.def_ner.none	0.429	0.423	0.426	0.676	0.589	0.629	0.567	0.557	0.562	0.539
CF.ncbi_pubmed_mimic_l.24_tok.wordp_lc_sw.none_cf.none_ner.none	0.488	0.528	0.507	0.622	0.590	0.605	0.504	0.498	0.501	0.538
SW.ncbi_pubmed_mimic_l.24_tok.wordp_lc_sw.none_cf.def_ner.none	0.456	0.524	0.488	0.659	0.617	0.637	0.469	0.489	0.479	0.534
CF.ncbi_pubmed_mimic_l.24_tok.wordp_lc_sw.none_cf.def_ner.none	0.456	0.524	0.488	0.659	0.617	0.637	0.469	0.489	0.479	0.534
LC.ncbi_pubmed_mimic_l.24_tok.wordp_lc_sw.none_cf.def_ner.none	0.456	0.524	0.488	0.659	0.617	0.637	0.469	0.489	0.479	0.534
LC.ncbi_pubmed_mimic_l.24_tok.wordp_notlc_sw.none_cf.def_ner.none	0.456	0.524	0.488	0.659	0.617	0.637	0.469	0.489	0.479	0.534
CF.biob_v1.0_pubm+pmc_tok.wordp_lc_sw.none_cf.none_ner.none	0.473	0.463	0.468	0.565	0.467	0.511	0.592	0.644	0.617	0.532
CF.ncbi_pubmed_mimic_l.12_tok.wordp_lc_sw.none_cf.none_ner.none	0.404	0.427	0.415	0.669	0.544	0.600	0.539	0.558	0.548	0.521
SW.ncbi_pubmed_mimic_l.12_tok.wordp_lc_sw.nltk_cf.def_ner.none	0.324	0.445	0.375	0.690	0.605	0.645	0.523	0.559	0.540	0.520
SW.biob_v1.0_pmc_tok.wordp_lc_sw.biosses_cf.def_ner.none	0.480	0.397	0.435	0.638	0.569	0.602	0.508	0.526	0.517	0.518
SW.biob_large_v1.1_pubmed_tok.wordp_lc_sw.biosses_cf.def_ner.none	0.388	0.361	0.374	0.671	0.585	0.625	0.546	0.545	0.546	0.515
SW.ncbi_pubmed_mimic_l.12_tok.wordp_lc_sw.biosses_cf.def_ner.none	0.298	0.404	0.343	0.691	0.607	0.646	0.533	0.574	0.553	0.514
SW.ncbi_pubmed_l.12_tok.wordp_lc_sw.nltk_cf.def_ner.none	0.249	0.312	0.277	0.680	0.601	0.638	0.612	0.640	0.626	0.514
SW.ncbi_pubmed_l.24_tok.wordp_lc_sw.none_cf.def_ner.none	0.333	0.417	0.370	0.587	0.571	0.579	0.577	0.600	0.588	0.512
CF.ncbi_pubmed_l.24_tok.wordp_lc_sw.none_cf.def_ner.none	0.333	0.417	0.370	0.587	0.571	0.579	0.577	0.600	0.588	0.512
LC.ncbi_pubmed_l.24_tok.wordp_lc_sw.none_cf.def_ner.none	0.333	0.417	0.370	0.587	0.571	0.579	0.577	0.600	0.588	0.512
LC.ncbi_pubmed_l.24_tok.wordp_notlc_sw.none_cf.def_ner.none	0.333	0.417	0.370	0.587	0.571	0.579	0.577	0.600	0.588	0.512
SW.biob_v1.0_pmc_tok.wordp_lc_sw.nltk_cf.def_ner.none	0.476	0.404	0.437	0.639	0.572	0.604	0.486	0.488	0.487	0.509
CF.PubMedBERT.abs+full_tok.wordp_lc_sw.none_cf.none_ner.none	0.363	0.393	0.377	0.640	0.541	0.587	0.519	0.586	0.551	0.505
CF.scibert_scivocab_tok.wordp_lc_sw.none_cf.none_ner.none	0.411	0.451	0.430	0.554	0.525	0.539	0.520	0.560	0.539	0.503
SW.ncbi_pubmed_l.24_tok.wordp_lc_sw.nltk_cf.def_ner.none	0.244	0.314	0.275	0.635	0.544	0.586	0.622	0.662	0.642	0.501
SW.ncbi_pubmed_l.12_tok.wordp_lc_sw.biosses_cf.def_ner.none	0.204	0.279	0.236	0.680	0.604	0.640	0.598	0.634	0.616	0.497
SW.biob_v1.0_pubmed_tok.wordp_lc_sw.biosses_cf.def_ner.none	0.382	0.380	0.381	0.624	0.576	0.599	0.498	0.508	0.503	0.494
SW.ncbi_pubmed_mimic_l.24_tok.wordp_lc_sw.biosses_cf.def_ner.none	0.350	0.414	0.379	0.693	0.576	0.629	0.460	0.478	0.469	0.492
CF.PubMedBERT.abs+full_tok.wordp_lc_sw.none_cf.biosses_ner.none	0.352	0.387	0.369	0.621	0.521	0.567	0.502	0.576	0.536	0.491
SW.biob_v1.0_pubmed_tok.wordp_lc_sw.nltk_cf.def_ner.none	0.367	0.359	0.363	0.626	0.576	0.600	0.497	0.517	0.507	0.490
CF.PubMedBERT.abs+full_tok.wordp_lc_sw.none_cf.blagec_ner.none	0.333	0.366	0.349	0.622	0.519	0.566	0.501	0.575	0.536	0.483
SW.biob_v1.0_pubm+pmc_tok.wordp_lc_sw.biosses_cf.def_ner.none	0.410	0.339	0.371	0.625	0.561	0.591	0.484	0.488	0.486	0.483
SW.ncbi_pubmed_mimic_l.24_tok.wordp_lc_sw.nltk_cf.def_ner.none	0.348	0.411	0.377	0.685	0.574	0.625	0.437	0.457	0.447	0.483

Table A.15: Part 2 of 2: Pearson (r), Spearman (*ho*) and Harmonic score (h) obtained by the BERT similarity methods evaluated herein.

	BIOSSES			MedSTS			CTR			Avg
	r	ρ	h	r	ρ	h	r	ρ	h	Avg
SW.biob_v1.1_pubmed_tok.wordp_lc_sw.nltk_cf.def_ner.none	0.361	0.316	0.337	0.630	0.591	0.610	0.453	0.476	0.464	0.470
SW.ncbi_pubmed_l.24_tok.wordp_lc_sw.biosses_cf.def_ner.none	0.174	0.233	0.200	0.636	0.536	0.582	0.597	0.644	0.620	0.467
CF.clinicalb_tok.wordp_lc_sw.none_cf.blagec_ner.none	0.452	0.541	0.492	0.537	0.527	0.532	0.329	0.425	0.371	0.465
SW.biob_v1.1_pubmed_tok.wordp_lc_sw.biosses_cf.def_ner.none	0.322	0.313	0.317	0.635	0.590	0.612	0.451	0.477	0.464	0.464
SW.PubMedBERT.abstract_tok.wordp_lc_sw.none_cf.def_ner.none	0.449	0.487	0.467	0.552	0.450	0.496	0.365	0.488	0.418	0.460
CF.PubMedBERT.abstract_tok.wordp_lc_sw.none_cf.def_ner.none	0.449	0.487	0.467	0.552	0.450	0.496	0.365	0.488	0.418	0.460
LC.PubMedBERT.abstract_tok.wordp_lc_sw.none_cf.def_ner.none	0.449	0.487	0.467	0.552	0.450	0.496	0.365	0.488	0.418	0.460
LC.PubMedBERT.abstract_tok.wordp_notlc_sw.none_cf.def_ner.none	0.449	0.487	0.467	0.552	0.450	0.496	0.365	0.488	0.418	0.460
CF.dischsum_tok.wordp_lc_sw.none_cf.blagec_ner.none	0.393	0.496	0.438	0.552	0.533	0.543	0.354	0.452	0.397	0.459
CF.bio.dischsum_tok.wordp_lc_sw.none_cf.biosses_ner.none	0.430	0.505	0.464	0.530	0.525	0.528	0.343	0.419	0.377	0.456
CF.dischsum_tok.wordp_lc_sw.none_cf.biosses_ner.none	0.385	0.495	0.433	0.533	0.533	0.533	0.348	0.458	0.395	0.454
CF.clinicalb_tok.wordp_lc_sw.none_cf.none_ner.none	0.407	0.488	0.444	0.546	0.520	0.533	0.353	0.406	0.378	0.451
CF.bio.clinicalb_tok.wordp_lc_sw.none_cf.biosses_ner.none	0.387	0.521	0.444	0.537	0.530	0.534	0.336	0.408	0.368	0.449
CF.clinicalb_tok.wordp_lc_sw.none_cf.biosses_ner.none	0.382	0.524	0.442	0.524	0.524	0.524	0.328	0.410	0.364	0.443
CF.bio.clinicalb_tok.wordp_lc_sw.none_cf.none_ner.none	0.368	0.444	0.402	0.536	0.509	0.522	0.372	0.442	0.404	0.443
CF.dischsum_tok.wordp_lc_sw.none_cf.none_ner.none	0.375	0.465	0.415	0.543	0.512	0.527	0.358	0.411	0.383	0.442
CF.bio.dischsum_tok.wordp_lc_sw.none_cf.blagec_ner.none	0.410	0.480	0.443	0.525	0.520	0.522	0.311	0.416	0.356	0.440
CF.bio.dischsum_tok.wordp_lc_sw.none_cf.none_ner.none	0.379	0.462	0.416	0.539	0.511	0.525	0.350	0.410	0.378	0.439
SW.clinicalb_tok.wordp_lc_sw.nltk_cf.def_ner.none	0.379	0.374	0.376	0.619	0.545	0.580	0.349	0.364	0.356	0.437
CF.bio.clinicalb_tok.wordp_lc_sw.none_cf.blagec_ner.none	0.379	0.406	0.392	0.530	0.514	0.522	0.365	0.418	0.389	0.435
LC.bio.dischsum_tok.wordp_lc_sw.none_cf.def_ner.none	0.411	0.457	0.432	0.494	0.529	0.511	0.329	0.397	0.360	0.434
SW.bio.clinicalb_tok.wordp_lc_sw.none_cf.def_ner.none	0.393	0.432	0.412	0.499	0.523	0.511	0.353	0.393	0.372	0.432
CF.clinicalb_tok.wordp_lc_sw.none_cf.def_ner.none	0.365	0.425	0.392	0.502	0.530	0.516	0.346	0.428	0.383	0.430
CF.dischsum_tok.wordp_lc_sw.none_cf.def_ner.none	0.369	0.408	0.388	0.507	0.528	0.517	0.348	0.418	0.380	0.428
CF.bio.clinicalb_tok.wordp_lc_sw.none_cf.def_ner.none	0.402	0.446	0.422	0.503	0.522	0.512	0.317	0.389	0.349	0.428
LC.bio.clinicalb_tok.wordp_lc_sw.none_cf.def_ner.none	0.360	0.393	0.376	0.487	0.517	0.502	0.383	0.432	0.406	0.428
SW.bio.dischsum_tok.wordp_lc_sw.biosses_cf.def_ner.none	0.313	0.346	0.329	0.615	0.550	0.581	0.366	0.373	0.369	0.426
SW.bio.clinicalb_tok.wordp_lc_sw.nltk_cf.def_ner.none	0.355	0.347	0.351	0.619	0.559	0.587	0.331	0.345	0.338	0.425
SW.bio.dischsum_tok.wordp_lc_sw.nltk_cf.def_ner.none	0.340	0.353	0.346	0.618	0.545	0.579	0.335	0.366	0.350	0.425
SW.bio.clinicalb_tok.wordp_lc_sw.biosses_cf.def_ner.none	0.331	0.345	0.337	0.624	0.545	0.582	0.343	0.361	0.352	0.424
LC.clinicalb_tok.wordp_lc_sw.none_cf.def_ner.none	0.399	0.425	0.412	0.507	0.518	0.512	0.314	0.383	0.345	0.423
SW.bio.dischsum_tok.wordp_lc_sw.none_cf.def_ner.none	0.413	0.456	0.433	0.500	0.507	0.504	0.309	0.360	0.332	0.423
LC.bio.dischsum_tok.wordp_notlc_sw.none_cf.def_ner.none	0.361	0.394	0.376	0.501	0.522	0.511	0.343	0.417	0.376	0.421
SW.dischsum_tok.wordp_lc_sw.biosses_cf.def_ner.none	0.326	0.317	0.321	0.619	0.545	0.579	0.357	0.358	0.357	0.419
LC.dischsum_tok.wordp_notlc_sw.none_cf.def_ner.none	0.379	0.437	0.406	0.496	0.515	0.505	0.323	0.374	0.346	0.419
SW.dischsum_tok.wordp_lc_sw.none_cf.def_ner.none	0.395	0.453	0.422	0.499	0.522	0.510	0.298	0.359	0.326	0.419
LC.clinicalb_tok.wordp_notlc_sw.none_cf.def_ner.none	0.328	0.405	0.363	0.507	0.524	0.515	0.347	0.413	0.377	0.418
SW.dischsum_tok.wordp_lc_sw.nltk_cf.def_ner.none	0.333	0.356	0.344	0.596	0.514	0.552	0.345	0.368	0.356	0.418
LC.bio.clinicalb_tok.wordp_notlc_sw.none_cf.def_ner.none	0.357	0.407	0.380	0.488	0.512	0.500	0.337	0.410	0.370	0.417
SW.clinicalb_tok.wordp_lc_sw.biosses_cf.def_ner.none	0.311	0.324	0.317	0.614	0.553	0.582	0.342	0.357	0.349	0.416
CF.bio.dischsum_tok.wordp_lc_sw.none_cf.def_ner.none	0.364	0.410	0.386	0.511	0.537	0.524	0.295	0.373	0.329	0.413
SW.clinicalb_tok.wordp_lc_sw.none_cf.def_ner.none	0.367	0.403	0.384	0.506	0.519	0.513	0.299	0.383	0.336	0.411
LC.dischsum_tok.wordp_lc_sw.none_cf.def_ner.none	0.357	0.386	0.371	0.507	0.525	0.516	0.312	0.372	0.339	0.409
CF.PubMedBERT.abstract_tok.wordp_lc_sw.none_cf.blagec_ner.none	0.374	0.398	0.385	0.491	0.391	0.435	0.318	0.443	0.370	0.397
CF.PubMedBERT.abstract_tok.wordp_lc_sw.none_cf.biosses_ner.none	0.376	0.406	0.390	0.491	0.391	0.435	0.291	0.427	0.346	0.390
CF.PubMedBERT.abstract_tok.wordp_lc_sw.none_cf.none_ner.none	0.348	0.377	0.362	0.467	0.363	0.409	0.310	0.425	0.359	0.377

Table A.16: Pearson (r), Spearman (*ho*) and Harmonic score (h) obtained by the NER experiments similarity methods evaluated herein.

	BIOSSES			MedSTS			CTR			Avg
	r	ρ	h	r	ρ	h	r	ρ	h	Avg
COM_WBSM_UBSM_Ctakes_Rada	0.793	0.809	0.801	0.773	0.708	0.739	0.789	0.783	0.786	0.776
UBSM_Ctakes_Rada	0.792	0.809	0.800	0.763	0.700	0.730	0.776	0.794	0.785	0.772
COM_WBSM_UBSM_MetamapLite_Rada	0.777	0.800	0.788	0.770	0.706	0.737	0.793	0.782	0.787	0.771
COM_WBSM_UBSM_Metamap_Rada	0.782	0.802	0.792	0.758	0.692	0.724	0.778	0.759	0.768	0.761
UBSM_MetamapLite_Rada	0.730	0.759	0.744	0.753	0.689	0.720	0.780	0.791	0.785	0.750
UBSM_Metamap_Rada	0.729	0.755	0.742	0.711	0.652	0.680	0.725	0.722	0.723	0.715
UBSM_Ctakes_CosNWJC	0.730	0.769	0.749	0.697	0.625	0.659	0.713	0.673	0.693	0.700
UBSM_MetamapLite_CosNWJC	0.667	0.691	0.678	0.722	0.659	0.689	0.753	0.713	0.732	0.700
UBSM_MetamapLite_WJC	0.615	0.648	0.631	0.699	0.638	0.667	0.708	0.642	0.674	0.657
UBSM_Ctakes_WJC	0.660	0.704	0.681	0.659	0.581	0.617	0.653	0.601	0.626	0.641
UBSM_Metamap_CosNWJC	0.647	0.665	0.656	0.675	0.608	0.640	0.575	0.530	0.551	0.616
UBSM_MetamapLite_Cai	0.545	0.579	0.562	0.686	0.628	0.656	0.642	0.576	0.607	0.608
UBSM_MetamapLite_JC	0.529	0.573	0.550	0.683	0.621	0.650	0.620	0.585	0.602	0.601
UBSM_Ctakes_Cai	0.600	0.633	0.616	0.635	0.552	0.591	0.597	0.548	0.571	0.593
UBSM_Ctakes_JC	0.579	0.611	0.595	0.634	0.549	0.588	0.567	0.539	0.552	0.578
UBSM_Metamap_WJC	0.544	0.531	0.537	0.637	0.575	0.605	0.449	0.420	0.434	0.525
UBSM_Metamap_Cai	0.418	0.420	0.419	0.606	0.555	0.580	0.314	0.322	0.318	0.439
UBSM_Metamap_JC	0.308	0.325	0.316	0.576	0.547	0.561	0.237	0.231	0.234	0.370
