# Supplementary material for: A reproducible experimental survey on biomedical sentence similarity: A string-based method sets the state of the art
Source: PLoS One. 2022 Nov 21;17(11):e0276539. doi: 10.1371/journal.pone.0276539 (PMC9678326; doi:10.1371/journal.pone.0276539)
Supplement: S3 Appendix — We provide the reproducibility protocol published at protocols.io [44] as supplementary material to allow the exact replication of all our experiments, methods, and results. (PDF) [file pone.0276539.s003.pdf]

OPEN ACCESS

A reproducibility protocol and dataset on the biomedical sentence similarity V.3

Alicia Lara Clares[1], Juan J. Lastra-Díaz[1], Ana Garcia-Serrano[1]

[1]UNED

3

Feb 20, 2022

1

protocol .

Alicia Lara Clares

This protocol introduces a set of reproducibility resources with the aim of allowing the exact replication of the experiments introduced by our main paper [1], which introduces the largest and for the first time reproducible experimental survey on biomedical sentence similarity. HESML V2R1 [2] is the sixth release of our Half-Edge Semantic Measures Library (HESML), which is a linearly scalable and efficient Java software library of ontology-based semantic similarity measures and Information Content (IC) models for ontologies like WordNet, SNOMED-CT, MeSH and GO.
This protocol sets a self-contained reproducibility platform which contains the Java source code and binaries of our main benchmark program, as well as a Docker image which allows the exact replication of our experiments in any software platform supported by Docker, such as all Linux-based operating systems, Windows or MacOS. All the necessary resources for executing the experiments are published in the permanent repository [3]

Our benchmark program is distributed with the UMLS SNOMED-CT and MeSH ontologies by courtesy of the US National Library of Medicine (NLM), as well as all needed software components with the aim of making the setup process easier. Our Docker image provides an exact virtual replica of the machine in which we ran our experiments, thus removing the need to carry-out any tedious setup process, such as the setup of the Named Entity Recognizer tools and other software components. (2022-02-20)

[1] Lara-Clares A, Lastra-Díaz JJ, Garcia-Serrano A. A reproducible experimental survey on biomedical sentence similarity: a string-based method sets the state of the art. Submitted to PLoS One. 2022.

[2] Lara-Clares A, Lastra-Díaz JJ, Garcia-Serrano A. HESML V2R1 Java software library of semantic similarity measures for the biomedical domain. e-cienciaDatos; 2022. doi:10.21950/DOI

[3] Lara-Clares, Alicia; Lastra-Díaz, Juan J.; Garcia-Serrano, Ana, 2021, "Reproducible experiments on word and sentence similarity measures for the biomedical domain",https://doi.org/10.21950/EPNXTR, e-cienciaDatos, V2

This is an open access protocol distributed under the terms of the **Creative Commons Attribution License** (https://creativecommons.org/licenses/by/4.0/), which permits unrestricted use, distribution, and reproduction in any medium, provided the original author and source are credited

http://hesml.lsi.uned.es/

Alicia Lara Clares, Juan J. Lastra-Díaz, Ana Garcia-Serrano 2022. A reproducibility protocol and dataset on the biomedical sentence similarity. **protocols.io** https://protocols.io/view/a-reproducibility-protocol-and-dataset-on-the-biom-b5ckq2uw
Alicia Lara Clares

protocol

Lara-Clares A, Lastra-Díaz JJ, Garcia-Serrano A. A reproducible experimental survey on biomedical sentence similarity: a string-based method sets the state of the art. Submitted to PLoS One. 2022.

hesml, sentence similarity, semantic similarity, ontologies, bert

protocol ,

Feb 19, 2022

Feb 20, 2022

58476

The Docker image provides all software pre-installed, which means that it is not necessary to install them to reproduce the results of this paper.

All the required materials to reproduce the experiments in this protocol are published in our reproducibility dataset

Lara-Clares, Alicia; Lastra-Díaz, Juan J.; Garcia-Serrano, Ana (2022). Reproducible experiments on word and sentence similarity measures for the biomedical domain. e-cienciaDatos, V2. https://doi.org/10.21950/EPNXTR

Our benchmarks can be reproduced in any Docker-complaint platform, such as Windows, MacOS or any Linux-based system by following a similar setup to that introduced herein.

This is an open access protocol distributed under the terms of the **Creative Commons Attribution License** (https://creativecommons.org/licenses/by/4.0/), which permits unrestricted use, distribution, and reproduction in any medium, provided the original author and source are credited

In order to obtain a decrypt password for downloading the required files, you should sign and obtain a license for the National Library of Medicine (NLM) of the United States to use the UMLS Metathesaurus databases, as well as SNOMED-CT and MeSH ontologies included in this Docker image. For this purpose, you should go top the NLM license page, https://uts.nlm.nih.gov//license.html. After that, you could write to eciencia@consorciomadrono.es to obtain the password to decrypt the file. Likewise, you should obtain and sign a Data User Agreement from the Mayo Clinic to use the MedSTS dataset by sending the authors the Data User Agreement form, https://n2c2.dbmi.hms.harvard.edu/data-use-agreement.

Installing Docker on Ubuntu

1 If Docker is not installed in your machine, instructions below install latest version of Docker CE. 5m
For further details, we refer the reader to the official Docker setup page https://docs.docker.com/install/linux/docker-ce/ubuntu/

First, we update the system:

```
sudo apt-get update
```

Ubuntu 20.04

We install the dependencies:

```
sudo apt-get install ca-certificates curl gnupg lsb-release && curl -fsSL https://download.docker.com/linux/ubuntu/gpg | sudo gpg --dearmor -o /usr/share/keyrings/docker-archive-keyring.gpg
```

Ubuntu 20.04

We set stable Docker release

```
echo  "deb [arch=$(dpkg --print-architecture) signed-by=/usr/share/keyrings/docker-archive-keyring.gpg] https://download.docker.com/linux/ubuntu $(lsb_release -cs) stable" | sudo tee /etc/apt/sources.list.d/docker.list > /dev/null
```

Ubuntu 20.04

This is an open access protocol distributed under the terms of the **Creative Commons Attribution License** (https://creativecommons.org/licenses/by/4.0/), which permits unrestricted use, distribution, and reproduction in any medium, provided the original author and source are credited

We install Docker engine

```
sudo apt-get update && sudo apt-get install docker-ce docker-ce-cli containerd.io
```

Ubuntu 20.04

If the installation detailed below fails, you can install Docker for Ubuntu:

```
sudo apt  install docker.io
```

Ubuntu 20.04

Downloading resources from the repository

2 Now, we download and decrypt the external resources such as pre-trained models and dependencies. 10h

First, we create a data directory which will contain all the datasets, pre-trained models and dependencies for executing the experiments

```
cd /home/[user]/Desktop && mkdir HESML_DATA && cd HESML_DATA
```

Ubuntu 20.04

Now, we download extract the BERT pretrained models compressed file (20,2 GB) to the HESML_DATA

```
wget https://doi.org/10.21950/BERTExperiments.tar.gz && tar xvf BERTExperiments.tar.gz
```

Ubuntu 20.04

This is an open access protocol distributed under the terms of the **Creative Commons Attribution License** (https://creativecommons.org/licenses/by/4.0/), which permits unrestricted use, distribution, and reproduction in any medium, provided the original author and source are credited

We also download and extract the pre-trained character and sentence embeddings models (20GB) in the same directory

```
wget
https://doi.org/10.21950/CharacterAndSentenceEmbeddings.tar.gz
&& tar xvf CharacterAndSentenceEmbeddings.tar.gz
```

Ubuntu 20.04

We download and extract the pre-trained word embedding models (40GB) in the same directory

```
wget https://doi.org/10.21950/WordEmbeddings.tar.gz && tar xvf
WordEmbeddings.tar.gz
```

Ubuntu 20.04

And finally, we download, decrypt and extract the rest of dependencies (10GB), such as datasets, UMLS, Java libraries, cTAKES, Metamap and MetamapLite.

```
sudo apt install -y ccrypt && wget
https://doi.org/10.21950/Dependencies.tar.gz.cpt && ccrypt -d
Dependencies.tar.gz.cpt
```

Ubuntu 20.04

In order to obtain a decrypt password for the Dependencies.tar.gz file, you should sign and obtain a license for the National Library of Medicine (NLM) of the United States to use the UMLS Metathesaurus databases, as well as SNOMED-CT and MeSH ontologies included in this Docker image. For this purpose, you should go top the NLM license page, https://uts.nlm.nih.gov//license.html. After that, you could write to eciencia@consorciomadrono.es to obtain the password to decrypt the file. Likewise, you should obtain and sign a Data User Agreement from the Mayo Clinic to use the MedSTS dataset by sending the authors the Data User Agreement form, https://n2c2.dbmi.hms.harvard.edu/data-use-agreement

This is an open access protocol distributed under the terms of the **Creative Commons Attribution License** (https://creativecommons.org/licenses/by/4.0/), which permits unrestricted use, distribution, and reproduction in any medium, provided the original author and source are credited

```
tar xvf Dependencies.tar.gz
```

Ubuntu 20.04

Now, we can remove all the compressed files

```
rm -r *.tar.gz
```

Ubuntu 20.04

At the end of this section, you should have a directory named HESML_DATA on your local machine with this file structure:

.
./ImportedLibs
./WordEmbeddings
./UMLS
./SentenceEmbeddings
./ReproducibleResults
./SentenceSimDatasets
./FlairEmbeddings
./public_mm_lite
./apache-ctakes-4.0.0.1-src
./BERTExperiments
./dist
./public_mm

Create and run a Docker container with HESML and dependencies

3 ⚠ 15m

In this step, we create and run a Docker container which have pre-installed all the necessary software for executing the experiments.

This is an open access protocol distributed under the terms of the **Creative Commons Attribution License** (https://creativecommons.org/licenses/by/4.0/), which permits unrestricted use, distribution, and reproduction in any medium, provided the original author and source are credited

We get the docker image from DockerHub

docker pull alicialara/hesml_v2r1:latest

We pull the HESML docker image from DockerHub, which contains all the pre-installed software for executing the experiments.

NOTE: Alternatively, the docker image can also be downloaded and extracted from our permanent repository:

Lara-Clares, Alicia; Lastra-Díaz, Juan J.; Garcia-Serrano, Ana (2022). Reproducible experiments on word and sentence similarity measures for the biomedical domain. e-cienciaDatos, V2. https://doi.org/10.21950/EPNXTR

In this case, you can import the Docker file by following the next command

wget https://doi.org/10.21950/hesml_STS_dockerRelease.tar.gz && tar xvf hesml_STS_dockerRelease.tar.gz && docker load --input hesml_STS_dockerRelease.tar.gz

Now, we create, run and attach to the Docker container named "HESMLV2R1" which will share a volume with the HESML_DATA directory.

NOTE: you have to modify the variable [PATH_TO_HESML_DATA_DIRECTORY] using the path from your local machine.

This is an open access protocol distributed under the terms of the **Creative Commons Attribution License** (https://creativecommons.org/licenses/by/4.0/), which permits unrestricted use, distribution, and reproduction in any medium, provided the original author and source are credited

```
docker run --name=HESMLV2R1 -it -v
[PATH_TO_HESML_DATA_DIRECTORY]/HESML_DATA/:/home/user/HESML_
alicialara/hesml_v2r1:latest /bin/bash
```

Ubuntu 20.04

In the following, we will be working on the Docker container, which has been attached in the previous step.

Now, we clone the HESML repository from Github

```
cd /home/user && git clone --branch HESML-STS_master_dev
https://github.com/jjlastra/HESML.git
```

Docker version 20.10.12

And we copy the external libraries and dependencies into the HESMLSTSclient directory and we copy the last HESML core jar file into the client directory

```
cd /home/user/HESML_DATA/ && cp -r dist/lib
/home/user/HESML/HESML_Library/HESMLSTSclient/dist && cd
/home/user/HESML/HESML_Library && cp HESML/dist/HESML-
V2R1.0.1.jar HESMLSTSclient/dist/lib
```

Docker version 20.10.12

At the end of this section, you should have the following directories in the /home/user directory of the Docker container:

.
./HESML
./HESML_DATA

- The HESML directory contains the sources from Github with all the necessary dependencies and libraries for executing the experiments.
- The HESML_DATA directory contains the pre-trained models, python virtual environments and the NER tools for executing the experiments

This is an open access protocol distributed under the terms of the **Creative Commons Attribution License** (https://creativecommons.org/licenses/by/4.0/), which permits unrestricted use, distribution, and reproduction in any medium, provided the original author and source are credited

Launch the Metamap and cTAKES services

4 The experiments evaluated herein use the Metamap [4], MetamapLite [5] and cTAKES [6] external NER tools to annotate CUI codes on the sentences. Thus, we have to launch the NER tools services following the next steps. 5m

[4] Aronson AR, Lang F-M. An overview of MetaMap: historical perspective and recent advances. J Am Med Inform Assoc. 2010;17: 229–236. doi:10.1136/jamia.2009.002733

[5] Demner-Fushman D, Rogers WJ, Aronson AR. MetaMap Lite: an evaluation of a new Java implementation of MetaMap. J Am Med Inform Assoc. 2017;24: 841–844. doi:10.1093/jamia/ocw177

[6] Savova GK, Masanz JJ, Ogren PV, Zheng J, Sohn S, Kipper-Schuler KC, et al. Mayo clinical Text Analysis and Knowledge Extraction System (cTAKES): architecture, component evaluation and applications. J Am Med Inform Assoc. 2010;17: 507–513. doi:10.1136/jamia.2009.001560

First, we open the Metamap directory

```
cd /home/user/HESML_DATA/public_mm
```

Docker version 20.10.12

We start the Metamap dependency services

```
./bin/skrmedpostctl start && ./bin/wsdserverctl start
```

We start the Metamap services.

Docker version 20.10.12

Note: Before executing the next step, wait until the following message appears (2-3 minutes): "WSD Server databases and disambiguation methods have been initialized." and press the "Enter" key.

Now, we start the Metamap service

This is an open access protocol distributed under the terms of the **Creative Commons Attribution License** (https://creativecommons.org/licenses/by/4.0/), which permits unrestricted use, distribution, and reproduction in any medium, provided the original author and source are credited

```
./bin/mmserver &
```

Docker version 20.10.12

Then, press "Enter" key and execute the next step using your UMLS KEY.

```
export ctakes_umls_apikey=[ENTER YOUR UMLS API KEY]
```

Docker version 20.10.12

In order to obtain a UMLS KEY, you should sign and obtain a license for the National Library of Medicine (NLM) of the United States to use the UMLS Metathesaurus databases, as well as SNOMED-CT and MeSH ontologies included in this Docker image. For this purpose, you should go top the NLM license page, https://uts.nlm.nih.gov//license.html.

At the end of this section, you should have initialized the NER tools services, and you can execute all the experiments evaluated in our primary paper:

Lara-Clares A, Lastra-Díaz JJ, Garcia-Serrano A. (2022). A reproducible experimental survey on biomedical sentence similarity: a string-based method sets the state of the art. Submitted to PLoS One.

UBUNTU-based instructions to run our benchmarks on a Docker container

5 The final step is the execution of the experiments evaluated in out primary paper. 1d

This is an open access protocol distributed under the terms of the **Creative Commons Attribution License** (https://creativecommons.org/licenses/by/4.0/), which permits unrestricted use, distribution, and reproduction in any medium, provided the original author and source are credited

Lara-Clares A, Lastra-Díaz JJ, Garcia-Serrano A. (2022). A reproducible experimental survey on biomedical sentence similarity: a string-based method sets the state of the art. Submitted to PLoS One.

To run the experiments, first step into the HESMLSTSclient directory

```
cd /home/user/HESML/HESML_Library/HESMLSTSclient/
```

Docker version 20.10.12

Before running the experiments, remove previous results and temporal files:

```
rm -r ../ReproducibleExperiments/BioSentenceSimilarity_paper/BioSente
&& rm -r
../ReproducibleExperiments/BioSentenceSimilarity_paper/BioSentenceS
&& rm Execution_times_* && rm -r tmp* && rm -r /tmp/tmp*
```

Docker version 20.10.12

Now, execute the HESMLSTSclient with the default options

```
java -jar -Xms30g dist/HESMLSTSclient.jar
```

Docker version 20.10.12

Note that this experiment take more than 24 hours of execution time in a desktop computer with an AMD Ryzen 7 5800x CPU (16 cores) with 64 Gb RAM and 2TB Gb SSD disk

This is an open access protocol distributed under the terms of the **Creative Commons Attribution License** (https://creativecommons.org/licenses/by/4.0/), which permits unrestricted use, distribution, and reproduction in any medium, provided the original author and source are credited

At the end of this section, you should find all the raw output files in your HESML_DATA directory

[PATH_TO_HESML_DATA_DIRECTORY]/HESML_DATA/ReproducibleResults/BioSentenceSimilarity_paper/BioSentenceSimFinalRawOutputFiles

```
.
├── raw_similarity_BIOSSES_BESTCOMBS.csv
├── raw_similarity_BIOSSES_COMBestWorst.csv
├── raw_similarity_BIOSSES_LiBlockNER.csv
├── raw_similarity_BIOSSES_NERexperiment.csv
├── raw_similarity_CTR_BESTCOMBS.csv
├── raw_similarity_CTR_COMBestWorst.csv
├── raw_similarity_CTR_LiBlockNER.csv
├── raw_similarity_CTR_NERexperiment.csv
├── raw_similarity_MedSTSFull_BESTCOMBS.csv
├── raw_similarity_MedSTSFull_COMBestWorst.csv
├── raw_similarity_MedSTSFull_LiBlockNER.csv
└── raw_similarity_MedSTSFull_NERexperiment.csv
```

These raw output files will be used in the post-processing stage to create the tables 8, 10-17, figure 5 and appendix A detailed in our primary paper [1].

5.1 [OPTIONAL] Running the pre-processing experiments

2w

In our primary paper [1], we also evaluate the pre-processing configurations of each method, which are detailed in tables 7 and 9, as well as the appendix B of the same paper. This pre-processing experiments are evaluated using the HESMLSTSImpactEvaluationclient software included in the HESML V2R1 software release [6].

[6] Lara-Clares A, Lastra-Díaz JJ, Garcia-Serrano A. HESML V2R1 Java software library of semantic similarity measures for the biomedical domain. e-cienciaDatos; 2022. doi:10.21950/DOI

It is important to note that the execution of the pre-processing experiments requires high computational requirements and running times (more than 2 weeks), since they perform more than 1100 pre-processing combinations in total.

To execute the pre-processing experiments, run the following commands

This is an open access protocol distributed under the terms of the **Creative Commons Attribution License** (https://creativecommons.org/licenses/by/4.0/), which permits unrestricted use, distribution, and reproduction in any medium, provided the original author and source are credited

```
cd /home/user/HESML_DATA/ && cp -r dist/lib
/home/user/HESML/HESML_Library/HESMLSTSImpactEvaluat
&& cd /home/user/HESML/HESML_Library && cp HESML/dist
V2R1.0.1.jar HESMLSTSImpactEvaluationclient/dist/lib
```

```
cd
/home/user/HESML/HESML_Library/HESMLSTSImpactEvaluat
&& java -jar -Xms30g dist/HESMLSTSImpactEvaluationclient
```

Docker version 20.10.12

Post-processing the experiments 20m

6 The post-processing stage use the RStudio software installed in the local machine to create the final latex tables and CSV files. 20m

NOTE: Now, the post-processing experiments are evaluated in the local machine, under the HESML_DATA directory. You can detach the HESMLV2R1 docker container by clicking the key sequence: CTRL+p, CTRL+q

In our experiments, we use the last release of RStudio software (Version 1.4) with R version 4.1.2 (2021-11-01). We also install the following packages for executing the post-processing scripts:

- collections
- kableExtra
- knitr
- readr
- stringr
- xtable
- dplyr
- ggpubr

This is an open access protocol distributed under the terms of the **Creative Commons Attribution License** (https://creativecommons.org/licenses/by/4.0/), which permits unrestricted use, distribution, and reproduction in any medium, provided the original author and source are credited

- ggqqplot
- ggpubr
- ggplot2

After executing the experiments, the raw output files, as well as the R post-processing scripts are automatically copied into the HESML_DATA directory, in a new directory named "ReproducibleResults". Before executing the post-processing scripts, it is necessary to modify the file permissions following the next step:

```
cd [PATH_TO_HESML_DATA_DIRECTORY]/HESML_DATA && sudo chmod -R 777 ReproducibleResults/
```

Ubuntu 20.04

The tables 8, 10-17, figure 5 and appendices A and B are created executing the following R scripts marked in **bold** as follows:

.[PATH_TO_HESML_DATA_DIRECTORY]/HESML_DATA/ReproducibleResults/Post-scripts
├── **bio_sentence_sim_tables.R**
├── **bio_analytics_biosses.R**
├── **bio_analytics_ctr.R**
├── **bio_analytics_medsts.R**
├── **bio_sentence_sim_allExperiments_analyzingtablesPreprocessing.R**
├── **bio_sentence_sim_pvaluesLiBlock.R**
├── **bio_sentence_sim_pvaluesNER.R**
├── **bio_sentence_sim_pvalues.R**
├── bio_sentence_sim_scripts
│ ├── readBERT.R
│ ├── readBESTCOMBS.R
│ ├── readFlair.R
│ ├── readLiBlockNERexperiment.R
│ ├── readNERexperiment.R
│ ├── readOurWE.R
│ ├── readSent2Vec.R
│ ├── readString.R
│ ├── readSWEM.R
│ ├── readTest.R
│ ├── readUBSM.R
│ ├── readUSE.R
│ └── readWBSM.R

This is an open access protocol distributed under the terms of the **Creative Commons Attribution License** (https://creativecommons.org/licenses/by/4.0/), which permits unrestricted use, distribution, and reproduction in any medium, provided the original author and source are credited

- **bio_sentence_sim_tables.R** : Creates the tables 8,10,11 and 12 in our primary paper [1] as well as all the tables from appendix B. It is also used to extract the best and worst pre-processing configuration in the table 9 of the same paper
- **bio_sentence_sim_pvalues.R** : Creates the tables of the appendix A in our primary paper [1].
- **bio_sentence_sim_allExperiments_analyzingtablesPreprocessing.R** : Creates the tables with all the p-values of the pre-processing experiments using the HESMLSTSImpactEvaluationclient, which are used in the table 9 of our main paper.
- **bio_sentence_sim_pvaluesLiBlock.R** : Creates a table with the LiBlock NER experiments which is used to detail the p-values in table 12 of the main paper [1].
- **bio_sentence_sim_pvaluesNER.R** : Creates a table with the NER experiments which is used to detail the p-values in table 11 of the main paper [1].
- **bio_analytics_biosses.R, bio_analytics_medsts.R and bio_analytics_ctr.R**: Creates the figure 5 and is used to create the tables 13-17 of our primary paper [1].

The "bio_sentence_sim_scripts" directory contains a set of R scripts to parse the output raw files created by the execution of HESMLSTSclient and HESMLSTSImpactEvaluationclient.

The execution of all the R scripts listed below produces a ser of TXT and CSV files containing all the post-processed results, which are used to create tables 8, 10-17, figure 5 and appendices A and B of our primary paper [1].

This is an open access protocol distributed under the terms of the **Creative Commons Attribution License** (https://creativecommons.org/licenses/by/4.0/), which permits unrestricted use, distribution, and reproduction in any medium, provided the original author and source are credited
