# Supplementary material for: A reproducible experimental survey on biomedical sentence similarity: A string-based method sets the state of the art
Source: PLoS One. 2022 Nov 21;17(11):e0276539. doi: 10.1371/journal.pone.0276539 (PMC9678326; doi:10.1371/journal.pone.0276539)
Supplement: Attachment — Submitted filename: Response to Reviewers.pdf [file pone.0276539.s004.pdf]

Madrid, 27 September 2022

Dear editor and reviewers,

We are very grateful for your significant effort to review our manuscript, as well as your kind remarks and suggestions to improve the quality of the paper.

We have accepted and followed [all] suggestions made by the reviewers. Likewise, the use of English has been revised by a native English-speaker linguist, as suggested by the Editor.

Finally, we provide below our detailed answer for each suggestion made by the reviewers.

Most sincerely,

The authors

—————-

- Editor reports:

The manuscript should be refined for English grammatical structure and phraseology. The manuscript should be polished by an English linguist or language service (note in marked-up copy text where changes are made). 'Grammarly' is poor quality and not acceptable.

[**Authors**] We apologize for any errors made in the use of English grammatical structure and phraseology. To solve this problem, this manuscript has been reviewed by a native English linguist, and we have marked-up all the changes.

- Reviewer reports:

The authors present a very extensive and detailed analysis of algorithms for computing semantic similarity of sentences from the biomedical literature. The work is relevant and well-executed.

[**Authors**] Thank you so much for your kind remarks.

My main source of confusion are the methods used for calculating statistical significance. First, the authors split a dataset into multiple artificially created sub-datasets to enable statistical significance testing. I wonder what the implications of this are, since the datasets so created come from the same data distribution. Please elucidate this a bit more, or point to references that discuss such a procedure from a statistical perspective (or overhaul this part of the analysis if it turns out that statistical significance cannot be calculated as orignally planned.)

[**Authors**] To clarify the issue mentioned above, we have included a detailed explanation in lines 384-413 on page 16 supported by the data and statistical tests shown in the new figure 5. In short, we detail and explain the statistical reasons that encouraged us to split MedSTS into smaller datasets for our statistical significance analysis. We show that the

harmonic score used as an evaluation metric is a random variable with different normal distributions depending on the size of the dataset used for its computation.

Second, it seems like many comparisons are made and no corrections for multiple testing are made. Please elucidate this and point out why this is not needed (or if it is needed, add corrections for multiple testing).

[**Authors**] We thank the reviewer for pointing out this flaw in our statistical significance analysis. For this reason, we have defined a Bonferroni correction to evaluate the statistical significance of multiple hypothesis tests involved in those conclusions in which one baseline sentence similarity measure is compared with a family of similarity measures, as detailed in lines 414-428 on page 16 of the revised manuscript. All the pairwise p-values keep the same values reported in our previous manuscript. However, we define a Bonferroni corrected null-hypothesis rejection threshold α_c for the old p-values, such that the null-hypothesis is now rejected only if "$p - value <= \alpha_c = 0.05/m$" for each conclusion, where 'm' is the number of comparisons.

Minor comments: Position 33-35: "cannot be reproduced because of the lack of source code and data" [...]: -¿ Please check if this strong statement is correct. For example, all code and data used in the study of Blagec et al. is available at [1] (and this link is provided in the manuscript)

[**Authors**] Done. We have included a proper explanation in section Introduction as shown in new lines 37-51. We have reviewed and refined the statement, providing more detailed information and links to the resources provided by the authors.

[1] https://github.com/kathrinblagec/neural-sentence-embedding-models-for-biomedical-applications
